# The ancestral activation promiscuity of ADP-glucose pyrophosphorylases from oxygenic photosynthetic organisms

**DOI:** 10.1186/1471-2148-13-51

**Published:** 2013-02-21

**Authors:** Misty L Kuhn, Carlos M Figueroa, Alberto A Iglesias, Miguel A Ballicora

**Affiliations:** 1 Department of Chemistry and Biochemistry, Loyola University Chicago, 1032 W. Sheridan Rd, Chicago, IL, 60660, USA; 2 Instituto de Agrobiotecnología del Litoral (UNL-CONICET), Facultad de Bioquímica y Ciencias Biológicas (UNL), Ciudad Universitaria, Santa Fe, S3000ZAA, Argentina; 3 Present address: Department of Molecular Pharmacology and Biological Chemistry, Northwestern University Feinberg School of Medicine, 303 E. Chicago Avenue, Chicago, IL, 60611, USA

## Abstract

**Background:**

ADP-glucose pyrophosphorylase (ADP-Glc PPase) catalyzes the first committed step in the synthesis of glycogen in bacteria and starch in algae and plants. In oxygenic photosynthetic organisms, ADP-Glc PPase is mainly activated by 3-phosphoglycerate (3-PGA) and to a lesser extent by other metabolites. In this work, we analyzed the activation promiscuity of ADP-Glc PPase subunits from the cyanobacterium *Anabaena* PCC 7120, the green alga *Ostreococcus tauri*, and potato (*Solanum tuberosum*) tuber by comparing a specificity constant for 3-PGA, fructose-1,6-bisphosphate (FBP), fructose-6-phosphate, and glucose-6-phosphate.

**Results:**

The 3-PGA specificity constant for the enzymes from *Anabaena* (homotetramer), *O. tauri*, and potato tuber was considerably higher than for other activators. *O. tauri* and potato tuber enzymes were heterotetramers comprising homologous small and large subunits. Conversely, the *O. tauri* small subunit (OtaS) homotetramer was more promiscuous because its FBP specificity constant was similar to that for 3-PGA. To explore the role of both OtaS and OtaL (*O. tauri* large subunit) in determining the specificity of the heterotetramer, we knocked out the catalytic activity of each subunit individually by site-directed mutagenesis. Interestingly, the mutants OtaS_D148A_/OtaL and OtaS/OtaL_D171A_ had higher specificity constants for 3-PGA than for FBP.

**Conclusions:**

After gene duplication, OtaS seemed to have lost specificity for 3-PGA compared to FBP. This was physiologically and evolutionarily feasible because co-expression of both subunits restored the specificity for 3-PGA of the resulting heterotetrameric wild type enzyme. This widespread promiscuity seems to be ancestral and intrinsic to the enzyme family. Its presence could constitute an efficient evolutionary mechanism to accommodate the ADP-Glc PPase regulation to different metabolic needs.

## Background

Many organisms accumulate carbon and energy as polyglucans. Glycogen is the main storage polysaccharide found in bacteria, fungi, and mammals. Similarly, green algae and plants accumulate starch, which is composed of two different molecules: amylose and amylopectin [1,2]. The biosynthesis of these polyglucans occurs by the usage of “activated” glucose molecules: UDP-glucose serves as glycosyl donor for the synthesis of glycogen in fungi and mammals, while ADP-glucose (ADP-Glc) is the initial substrate for the synthesis of glycogen in bacteria and starch in green algae and plants [1,2].

ADP-Glc pyrophosphorylase (ADP-Glc PPase, EC 2.7.7.27) catalyzes the synthesis of ADP-Glc from ATP and glucose-1-phosphate (Glc1P), in presence of a divalent cation (Mg^2+^) [1,2]. The enzyme from cyanobacteria is a tetramer composed of four identical subunits, whereas the enzyme from unicellular algae and plants is a heterotetramer composed of two small (S) and two large (L) subunits [1,2]. The homotetrameric enzyme from cyanobacteria shares a higher identity with plant S subunits than with other heterotrophic bacteria. In eukaryotes, the L subunit appeared later in evolution, most likely through gene duplication. Even though both subunits seem to have evolved from the same ancestor, the higher homology among the S subunits suggests stronger evolutionary constraints for them than for the L subunits [2,3].

The S and L subunits have undergone a subfunctionalization process; i.e. each subunit retained and/or lost particular properties that were already present in the common ancestor [4]. The S subunit from the potato tuber ADP-Glc PPase has catalytic activity and a low affinity (mM range) for the allosteric activator (3-phosphoglycerate, 3-PGA), whereas the L subunit is non-catalytic but its co-expression with the S subunit increases the affinity (μM range) for the activator of the resulting heterotetramer [5]. Similarly, co-expression of the S subunit (APS1) with different L subunit isoforms (APL1-4) of the *Arabidopsis thaliana* ADP-Glc PPase rendered heterotetrameric enzymes with distinctive kinetic and regulatory properties [6]. It was later demonstrated that the APL1 and APL2 subunits from the *A. thaliana* enzyme are also catalytic [7], which confers a higher degree of complexity to the synthesis of ADP-Glc in plants.

In most organisms, the activity of ADP-Glc PPase is modulated by key metabolites of the main carbon assimilatory pathway. ADP-Glc PPases characterized to date are usually activated by molecules that represent a “high energy status”, such as 3-PGA, fructose-1,6-bisphosphate (FBP), fructose-6-phosphate (Fru6P), and pyruvate. Conversely, these enzymes are typically inhibited by metabolites that indicate a “low energy status”, like inorganic orthophosphate (Pi), AMP, and ADP [1,2]. In addition, the reaction catalyzed by ADP-Glc PPase uses ATP as a substrate. Thus, it is widely accepted that accumulation of glycogen and starch will occur under conditions where carbon and energy are available, whereas their synthesis will be restricted in the opposite situations [1,2].

ADP-Glc PPases have been classified into nine different groups based in their quaternary structure and main allosteric effectors [1,2]. Group I contains enzymes from enteric bacteria (like *Escherichia coli*) utilizing the Embden-Meyerhof (glycolysis) pathway, which are activated by FBP and inhibited by AMP. Group IV is composed by ADP-Glc PPases from heterotrophic bacteria (such as *Agrobacterium tumefaciens*) utilizing the Entner-Doudoroff pathway, which are activated by Fru6P and pyruvate, and inhibited by AMP and ADP [1,2]. There is great diversity of activators among different bacterial ADP-Glc PPases, and secondary activators exist within some of the classes [8-11]. Recently, we have found that the enzyme from *Streptomyces coelicor* has a very different specificity for activators and may fall into a new class. It is activated mainly by glucose-6-phosphate (Glc6P) and can also be activated by mannose-6-phosphate, phosphoenolpyruvate, and Fru6P [12]. The enzyme from *Nitrosomonas europaea* can be activated mainly by pyruvate, but also by oxaloacetate and phosphoenolpyruvate [13]. The rationale for this diversity of regulatory molecules in different bacteria is that synthesis of ADP-Glc is strictly regulated by the main metabolic pathways operating in the respective organism [1].

Group VIII includes enzymes from cyanobacteria, green algae, and plants, which are activated by 3-PGA and inhibited by inorganic orthophosphate (Pi). The activator plays a key role in enzyme regulation, but it is the 3-PGA/Pi ratio that controls the activity of ADP-Glc PPase [14]. Group IX is composed of ADP-Glc PPases from plant non-photosynthetic tissues (such as maize, barley and wheat endosperm), which are inhibited by Pi, ADP, and FBP. This inhibitory effect can be reversed by 3-PGA and Fru6P [1,2]. It is well documented that 3-PGA is the main activator of ADP-Glc PPases from oxygenic photosynthetic organisms and that these two classes have been the most specific ones within the family. There have been some hints in the literature, however, that indicate that under certain circumstances some of the enzymes activated by 3-PGA could use other metabolites, but inefficiently. The enzymes from *Synechocystis* PCC 6803 and *Anabaena* PCC 7120 (cyanobacteria) [15], the green alga *Chlamydomonas reinhardtii*[16], spinach [17] and wheat [18] leaves, and maize endosperm [19] showed enhanced activities at high concentrations of other metabolites, including FBP, Fru6P, and Glc6P. This apparent omnipresent promiscuity in divergent organisms suggests that this behavior has a very distant common ancestry. Nevertheless, a thorough analysis in photosynthetic organism has not been performed. In this work, we analyzed the activation specificity of the enzymes from *Anabaena* PCC 7120, *Ostreococcus tauri*, and potato tuber. Our data support the idea that ADP-Glc PPases evolved to accommodate the regulatory properties for particular metabolic scenarios operating in different organisms.

## Results and discussion

### Phylogenetic analysis and activator promiscuity of ADP-Glc PPases

The sequence similarity between bacterial and the S and L subunits of eukaryotic ADP-Glc PPases suggests a common origin. It is believed that the ancestral enzyme may have been a bacterial subunit with both catalytic and regulatory functions [2]. Based on the roles of different S and L subunits from plants, it seems that after gene duplication ADP-Glc PPase has undergone a subfunctionalization process in which each copy adopted complementary roles [4]. The interaction between the product of these gene copies is now necessary to maintain the same overall set of functions of the common ancestor [4]. This duplication and divergence increased the sequence landscape and enabled ADP-Glc PPase to evolve towards more complex regulation. For instance, several isoforms of the L subunit are differentially expressed based on the type of tissue in the plant. This provides a variety of allosteric properties and seems to be the underlying mechanism for how starch synthesis is regulated [6,20].

**Figure 1:**
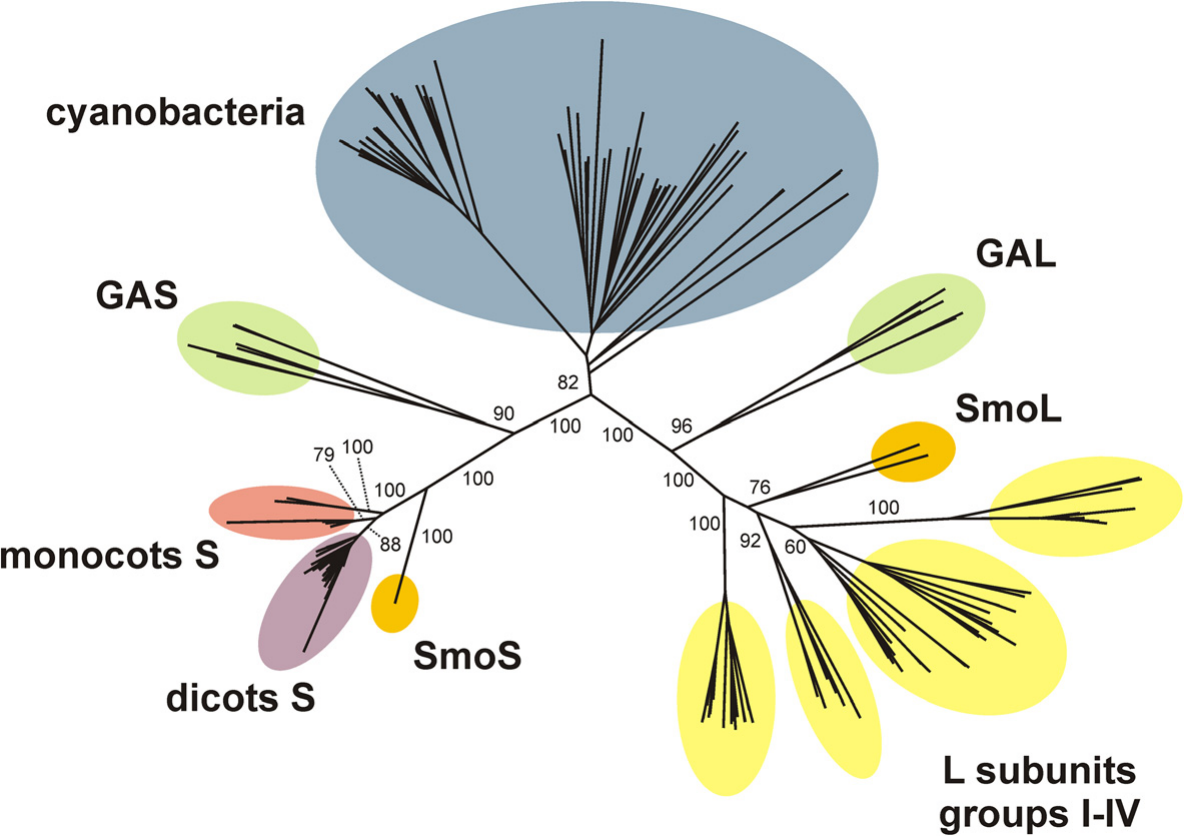
**Phylogenetic tree of ADP-Glc PPases from oxygenic photosynthetic organisms.** The tree is clearly divided into three major branches corresponding to enzymes from cyanobacteria (blue) and small and large subunits from green algae and plants. The small subunits are further divided into five branches: green algae (GAS, green), *S. moellendorffii* (SmoS, orange); dicots (violet); and monocots (two branches, red). The large subunits are divided into six branches: green algae (GAL, green); *S. moellendorffii* (SmoL, orange); and four groups (I-IV) from dicots and monocots (yellow). Numbers correspond to the bootstrap values for the indicated branches.

To follow function throughout sequence divergence, we aligned ADP-Glc PPase sequences (Additional file 1 Figure S1 and Additional file 2 Table S1) and constructed a neighbor-joining phylogenetic tree (Figure 1) using 68, 12, and 119 ADP-Glc PPase sequences from cyanobacteria, green algae, and plants (including the moss *Selaginella moellendorffii*), respectively. Figure 1 shows that the tree contains three major branches: cyanobacteria, small subunits, and large subunits from green algae and plants. The small subunits are grouped in five branches: green algae (GAS), *S. moellendorffii* (SmoS), dicots, and monocots (two groups). Large subunits are grouped in six branches: green algae (GAL), *S. moellendorffii* (SmoL), and four groups (I-IV) from dicots and monocots (Figure 1). The shape of the tree is similar to those previously obtained by Ballicora et al. [21] and Georgelis et al. [3].

Although ADP-Glc PPases from cyanobacteria, green algae, and plants are grouped in different branches of the phylogenetic tree (Figure 1), they share the same main heterotropic activator (3-PGA). In addition, all of them are activated by other metabolites. To study the evolution and promiscuity of the allosteric regulation in ADP-Glc PPases from divergent oxygenic photosynthetic organisms, we expressed and purified evolutionarily distant enzyme representatives from *Anabaena*, *O. tauri*, and potato tuber. Highly purified enzymes were used to determine the *V*_max_ in the presence and the absence of activator (to calculate the “net activation fold”) and the respective *A*_0.5_ values for the activation kinetics by 3-PGA, FBP, Fru6P, or Glc6P of each enzyme (see Table 1). With these values, we calculated the “specificity constant” as the ratio between the respective net activation fold over *A*_0.5_. This parameter is analogous to the catalytic efficiency, which is the parameter of choice to compare the substrate specificity of enzymes with simple hyperbolic kinetics [22].

**Table 1:** **Activation kinetics of ADP-Glc PPases from*Anabaena*,*O. tauri*, and potato tuber**

**Enzyme**	***v***_**0**_**(U mg**^**-1**^**)**	**Activator**	***A***_**0.5**_**(mM)**	**Activation fold**	**Net activation fold**	**Specificity constant (mM**^**-1**^**)**
*Anabaena*	0.77	3-PGA	0.075	5.5	4.5	60
		FBP	0.30	1.2	0.2	0.7
		Fru6P	1.6	2.5	1.5	1.0
		Glc6P	0.40	1.4	0.4	1.0
OtaS/OtaL	0.89	3-PGA	0.54	33	32	59
		FBP	1.1	7.0	6.0	5.5
		Fru6P	15	2.7	1.7	0.1
		Glc6P	75	17	16	0.2
StuS/StuL	0.27	3-PGA	0.054	68	67	1241
		FBP	0.84	6.0	5.0	6.0
		Fru6P	1.5	57	56	36
		Glc6P	1.8	29	28	15
OtaS	0.25	3-PGA	1.2	10	9.0	8.0
		FBP	1.1	9.3	8.3	7.6
		Fru6P	1.9	3.9	2.9	1.6
		Glc6P	10	5.5	4.5	0.4
OtaS_D148A_/OtaL	0.57	3-PGA	0.003	7.0	6.0	2000
		FBP	8.2	5.0	4.0	0.5
		Fru6P	0.74	5.0	4.0	5.4
		Glc6P	65	26	25	0.4
OtaS/OtaL_D171A_	0.12	3-PGA	0.067	5.1	4.1	61
		FBP	1.5	5.9	4.9	3.3
		Fru6P	2.9	5.0	4.0	1.4
		Glc6P	3.1	3.4	2.4	0.8

Activity of the purified enzymes was assayed in the ADP-Glc synthesis direction. Kinetic parameters were calculated as described under “Methods”. For each enzyme and each activator the *V*_max_ can be calculated as *v*_0_ times the Activation Fold. StuS and StuL are the S and L subunits of potato tuber, and OtaS and OtaL the S and L subunits of *O. tauri*, respectively.

**Figure 2:**
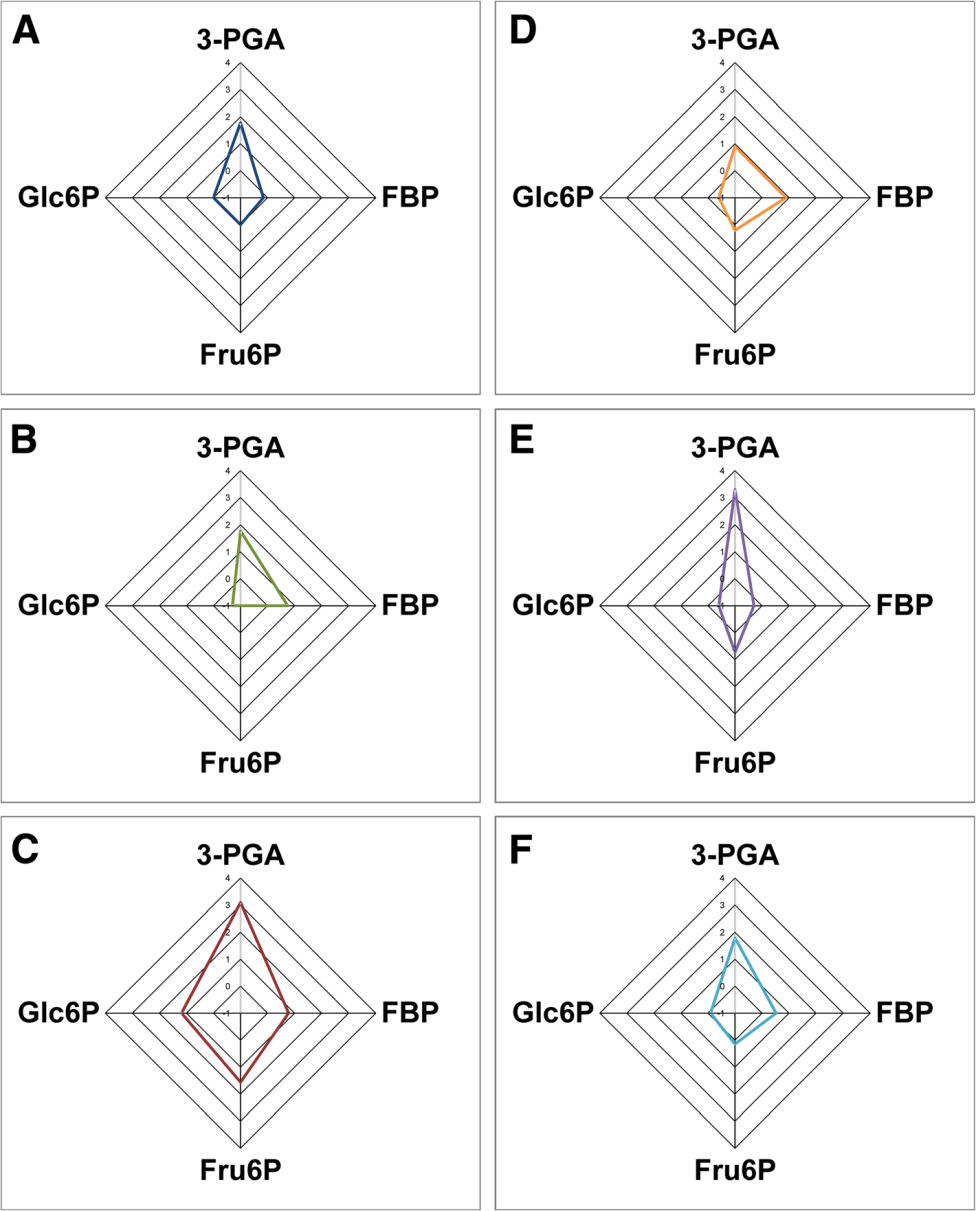
**Radial plots of the specificity constants for the activators.** Plots were constructed using the log_10_ of the specificity constants for 3-PGA, FBP, Fru6P, and Glc6P obtained with different ADP-Glc PPases (see Table 1). (**A**) *Anabaena*, (**B**) OtaS/OtaL, (**C**) StuS/StuL, (**D**) OtaS, (**E**) OtaS_D148A_/OtaL, and (**F**) OtaS/OtaL_D171A_.

The tested effectors activated all enzymes to different extents (Table 1); however, the specificity constants clearly show that 3-PGA is the most efficient activator for all three wild type enzymes. To analyze and compare this promiscuous behavior toward different effectors, we plotted the specificity constant for each activator of all enzymes. For the sake of clarity, we calculated the log_10_ of the specificity constants and constructed radial plots (see Figure 2). Plots in Figures 2A-C show that the activation promiscuity profile is similar for the enzymes from *Anabaena*, *O. tauri*, and potato tuber (their 3-PGA specificity constants are higher than the ones for FBP, Fru6P, and Glc6P).

Interestingly, when we analyzed the homotetrameric *O. tauri* ADP-Glc PPase (i.e. OtaS without OtaL) we found that the specificity constant for 3-PGA and FBP were similar (Table 1 and Figure 2D). This is a markedly different behavior compared to that observed for the native heterotetrameric form (OtaS/OtaL) and the ones from *Anabaena* and potato tuber (StuS/StuL). As previously reported [4], both OtaS and OtaL are catalytic but no activity was detected when OtaL was expressed in the absence of the S subunit. To better understand the contribution of OtaS and OtaL to the activation promiscuity of the heterotetrameric enzyme, we co-expressed the non-catalytic mutants OtaS_D148A_ and OtaL_D171A_ with the wild type counterparts, as previously described [4].

**Figure 3:**
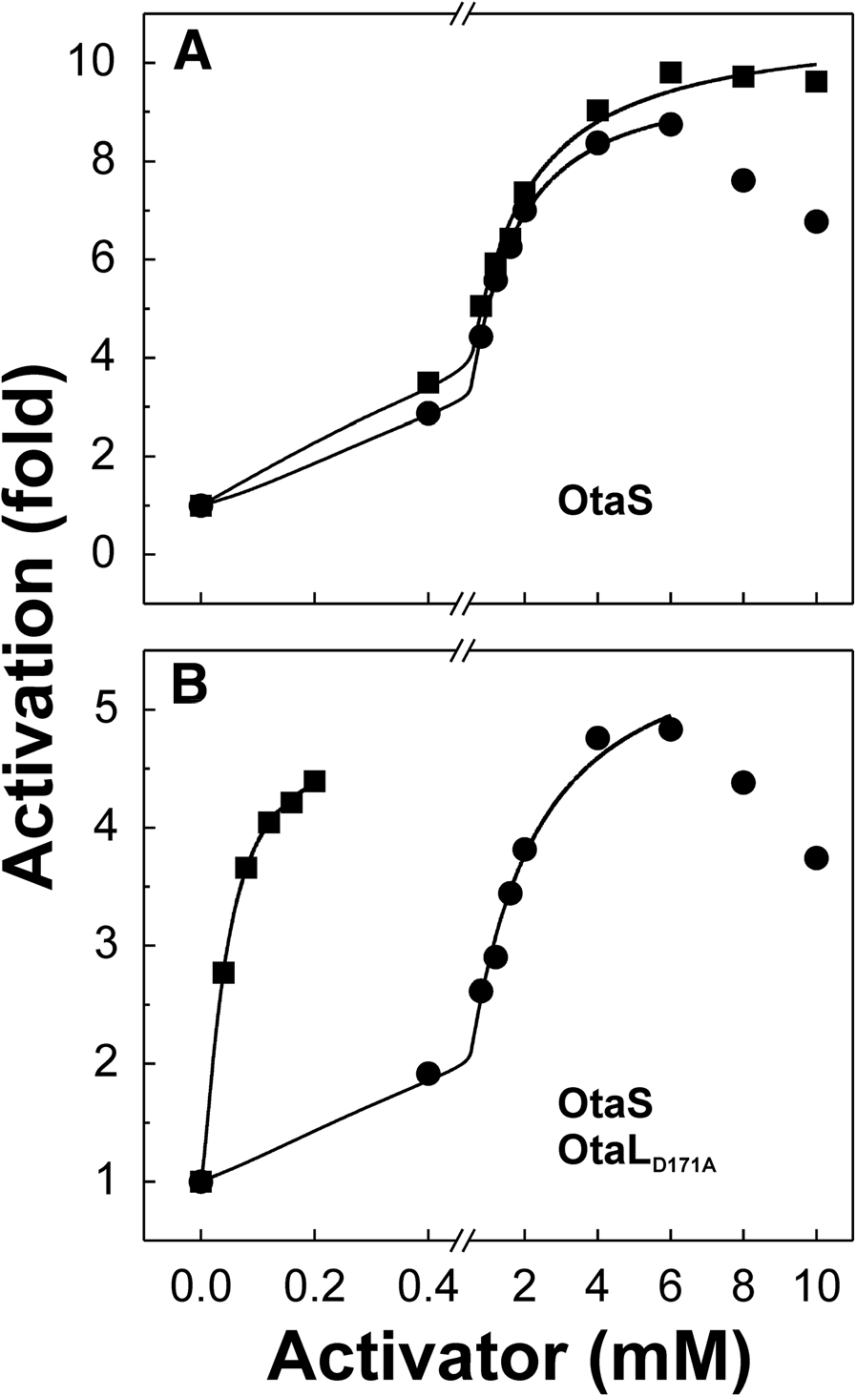
**Effect of OtaL on the affinity of OtaS for 3-PGA.** The OtaS homotetramer is similarly activated by 3-PGA and FBP, whereas the mutant OtaS/OtaL_D171A_ heterotetramer has a higher affinity for 3-PGA than FBP. The plots show the activation of OtaS (**A**) and OtaS/OtaL_D171A_ (**B**) by 3-PGA (▪) and FBP (·).

Contrary to what we observed in the homotetrameric OtaS enzyme, the mutant OtaS_D148A_/OtaL had a specificity constant for 3-PGA three orders of magnitude higher than for FBP (2000 and 0.5 mM^-1^, respectively) (Table 1 and Figure 2E). It is worth noting that in this mutant the second activator was Fru6P, with a specificity constant of 5.4 mM^-1^, a different trend compared to OtaS/OtaL and OtaS where the second activator was FBP (Table 1). A similar result was observed for the mutant OtaS/OtaL_D171A_, which had a specificity constant for 3-PGA that was higher than that for FBP (61 and 3.3 mM^-1^, respectively) (Table 1 and Figure 2F). Results obtained with this mutant resemble those for OtaS/OtaL, where FBP is a more efficient activator than Fru6P (Table 1). The effect of the addition of OtaL_D171A_ to OtaS on the specificity for activators is particularly striking at lower concentrations (Figure 3). Between 0.1-0.2 mM OtaS/OtaL_D171A_ is almost fully activated by 3-PGA but only slightly by FBP. On the other hand, OtaS is poorly activated by both.

### OtaL is an activator specifier

We analyzed the activation specificity constants for 3-PGA, FBP, Fru6P, and Glc6P in very divergent ADP-Glc PPases from oxygenic photosynthetic organisms. As expected, the highest values were observed for 3-PGA. However, the OtaS homotetramer has similar specificity constants for 3-PGA and FBP, indicating a loss of specificity. We determined that the specificity constants for 3-PGA in the heterotetrameric mutants OtaS_D148A_/OtaL and OtaS/OtaL_D171A_ are higher than for FBP, a trend similar to that observed for the wild type enzyme (OtaS/OtaL). Our results suggest that after gene duplication OtaS lost specificity for 3-PGA (similar specificity constants for 3-PGA and FBP). This was physiologically possible because the co-expression with OtaL restored the specificity for 3-PGA of the resulting heterotetrameric enzyme. We could consider the OtaL subunit as a “specifier”, because its presence in the heterotetramer (OtaS/OtaL) only increases the specificity for 3-PGA. This scenario is similar to that observed for β-1,4-galactosyltransferase, which changes its substrate specificity when it interacts with the protein α-lactalbumin. The enzyme transfers galactose from UDP-galactose to N-acetylglucosamine, which constitutes its normal galactosyltransferase activity. In the presence of α-lactalbumin, it transfers galactose to glucose, which is its lactose synthase activity [23]. In this particular case the preference for substrate is being changed, while in the OtaS/OtaL enzyme the change is at the allosteric activator level. In plants, ADP-Glc PPase is expressed in both autotrophic (leaves) and heterotrophic (tuber, roots, and others) tissues [20]; thus, it is exposed to diverse metabolic scenarios. In certain situations, it could be an evolutionary advantage to have some degree of promiscuity for metabolites that are typically found in heterotrophic environments (hexose-P or hexose-bisP).

### Subfunctionalization as an evolutionary process in ADP-Glc PPases

The subfunctionalization of genetic processes has been described in detail [24]; however, biochemical examples of such process are limited [4,7,25,26]. We recently described that the evolution of ADP-Glc PPase subunits may have undergone a subfunctionalization process [21], and that their divergent functional paths could have been different within eukaryotes [4]. Subfunctions in different subunits (catalysis, regulation, and protein solubility) co-evolved in heterotetramers to maintain physiologically functional enzymes. We postulated that the gene duplication that generated these two types of subunits caused a likely evolutionarily unstable overlap of roles. This resulted in a defective catalysis in the L subunits, a defective regulation in the S subunit, and in some cases, insolubility of L subunits [4]. Previously, we proposed that organisms with ADP-Glc PPases that lie on different branches of the phylogenetic tree might not retain identical catalytic properties of the enzyme. This hypothesis could also be extended to include the divergence of specificity for activators, which has been presented in this study. We observed that the specificity for the activator of one of the subunits is defective (i.e. not specific for 3-PGA), but the overall specific role of the heterotetramer is conserved because of the presence of the other subunit.

## Conclusions

We observed a widespread promiscuity for activators of ADP-Glc PPase of oxygenic photosynthetic organisms that seems to be ancestral and intrinsic to the enzyme family. In addition, we found that the OtaS homotetramer has lost specificity for 3-PGA, probably after gene duplication in eukaryotes. However, the specificity is maintained in the heterotetramer because of the L subunit. For this reason, we could consider the OtaL subunit as a “specifier” for activators. To the best of our knowledge, this is the first time this type of mechanism has been observed. The described promiscuity and the functional complementation of subunits would be very effective to accommodate the regulatory properties of ADP-Glc PPases to a variety of metabolic scenarios operating in different organisms and tissues.

## Methods

### Materials

α-D-[U-^14^C]Glc1P was from PerkinElmer (Waltham, MA). Chemicals for enzyme activity assays were from Sigma (St. Louis, MO). All the other reagents were from the highest quality available.

### Enzyme expression and purification

*Escherichia coli* AC70R1-504 cells, which are defective in endogenous ADP-Glc PPase activity [27], were transformed or co-transformed with the appropriate plasmids to obtain the different constructs. The ADP-Glc PPase from *Anabaena* was expressed using the plasmid pAna3a [28], whereas the enzyme from potato tuber was obtained using the plasmids pML10 and pMON17336 [5]. The OtaS homotetramer, the wild type OtaS/OtaL, and the mutants OtaS_D148A_/OtaL and OtaS/OtaL_D171A_ heterotetramers were expressed using the plasmids pMAB5 and pMAB6 [4]. Transformed cells were grown at 37°C in LB medium supplemented with the proper antibiotic until OD_600_ ~1.2, and induced for 16 h at 25°C with 0.4 mM isopropyl-β-D-1-thiogalactopyranoside and 5 μg/ml nalidixic acid (when expressing the heterotetramers). Cells were harvested by centrifugation for 10 min at 5000 x *g* and 4°C, and stored at −20°C until use.

The cell paste was resuspended with *Buffer A* (50 mM HEPES pH 8.0, 5 mM MgCl_2_, 0.1 mM EDTA, 10% (w/v) sucrose) and disrupted by sonication. The resulting suspension was centrifuged 15 min at 30000 x *g* and 4°C, and the supernatant (crude extract) was loaded onto a 10 ml DEAE-Sepharose column (GE Healthcare, Piscataway, NJ). Proteins were eluted with a linear NaCl gradient (20 column volumes, 0–0.5 M) and fractions containing ADP-Glc PPase activity were pooled and precipitated with ammonium sulfate at 70% saturation. After centrifuging, the pellet was resuspended in *Buffer H* (*Buffer A* plus 1 M ammonium sulfate) and loaded onto two 1 ml Resource PHE columns (GE Healthcare) connected in tandem. Protein was eluted with a linear ammonium sulfate gradient (50 column volumes, 1–0 M) and fractions containing ADP-Glc PPase activity were pooled, washed with *Buffer A*, and concentrated using Amicon Ultra-15 centrifugal filter units (Millipore, Billerica, MA). Enzymes stored at −80°C under these conditions remained stable for at least 3 months.

### Enzyme activity assay

Enzyme activity was determined by measuring the production of ADP-^14^C]Glc from ^14^C]Glc1P, as previously described by Yep et al. [29]. Unless otherwise stated, the standard reaction mixture contained 50 mM HEPPS pH 8.0, 7 mM MgCl_2_, 1 mM ^14^C]Glc1P, 1 mM ATP, 0.2 mg/ml BSA, 0.5 U/ml inorganic pyrophosphatase, enzyme in a proper dilution, and varying amounts of activator. When activity of the potato tuber enzyme was assayed, 2 mM DTT was added to the reaction media. One unit of enzyme activity is defined as the amount of enzyme producing 1 μmol of ADP-^14^C]Glc in 1 min at 37°C.

### Kinetic characterization

Data of enzyme activity was plotted versus the concentration of activator using the program Origin 7.0 (OriginLab Corporation) and fitted to a modified Hill equation: *v*=*v*_0_+(*V*_max_-*v*_0_)*A^*n*H^/(*A*_0.5_^*n*H^+A^*n*H^), where *v* is the velocity at a certain concentration of activator (A), *v*_0_ is the velocity in absence of activator, *A*_0.5_ is the concentration of activator that produces 50% of the maximal velocity (*V*_max_), and *n*_H_ is the Hill coefficient. The activation fold was calculated as the ratio *V*_max_/*v*_0_, whereas the net activation fold, i.e. the activation that is over one, was calculated as (*V*_max_-*v*_0_)/*v*_0_. To evaluate the activation promiscuity of ADP-Glc PPases we defined the “specificity constant” as the ratio between the net activation fold and the *A*_0.5_.

### Protein methods

Protein concentration after purification was determined by UV absorbance at 280 nm using a NanoDrop 1000 spectrophotometer (Thermo Fisher Scientific, Wilmington, DE) and an extinction coefficient of 1 ml cm^-1^ mg^-1^.

### Phylogenetic analysis

Sequences coding for ADP-Glc PPases from cyanobacteria, green algae, and plants were downloaded from the NCBI database (http://www.ncbi.nlm.nih.gov/). They were classified into different groups using taxonomic data provided by the NCBI. Duplicates and incomplete sequences were manually discarded. The N-terminal sequence of enzymes from green algae and plants, including a chloroplast transit peptide, were manually trimmed. A preliminary alignment was constructed using the ClustalW multiple sequence alignment server (http://www.genome.jp/tools/clustalw/) [30]. The BioEdit 7.0 program (http://www.mbio.ncsu.edu/bioedit/bioedit.html) [31] was used to manually refine the alignment, considering structural data from the potato tuber ADP-Glc PPase [32] (i.e. insertions and deletions were preferably placed in loop regions). An unrooted neighbor-joining tree based on the refined alignment was constructed using the accessory application in the SeaView 4.3 program (http://pbil.univ-lyon1.fr/software/seaview.html) [33] with a bootstrap of 1000. Finally, the tree was prepared with the FigTree 1.3 program (http://tree.bio.ed.ac.uk/).

## Abbreviations

ADP-Glc: ADP-glucose; ADP-Glc PPase: ADP-glucose pyrophosphorylase; FBP: Fructose-1,6-bisphosphate; Fru6P: Fructose-6-phosphate; Glc1P: Glucose-1-phosphate; Glc6P: Glucose-6-phosphate; Pi: Inorganic orthophosphate.

## Competing interests

The authors of this work declare that they have no competing interests.

## Authors’ contributions

MLK and CMF designed and performed the experimental procedures. AAI and MAB conceived the study. All the authors participated in data analysis. The final manuscript was revised and approved by all the authors.

## Supplementary Material

Additional file 1: Figure S1 Sequence alignment used for the phylogenetic tree. The alignment was initially performed with the ClustalW server and manually refined to introduce insertions and deletions in loop regions (based in the crystal structure of the potato tuber ADP-Glc PPase), as described under “Methods”. During the manual refinement of the alignment, 10 sequences were removed prior to tree reconstruction. Sequences 83, 85, 86, 93, 202, and 205 were removed due to their similarity with the *A. thaliana* aps2, which has no detectable activity *in vitro*[6]. Sequences 65, 78, and 79 were removed because they have an insertion in a region predicted as a β-sheet and the sequences have not been subjected to the NCBI final revision. Sequence 40 was removed because its C-terminal region was incomplete. Residues are colored based on their chemical properties. Click here for file

Additional file 2: Table S1 Data of sequences used for the phylogenetic tree. The table contains the number used for each sequence, the corresponding NCBI accession number and annotation, the name of the organism, and the taxonomic group. Colors used are the same as in the phylogenetic tree (Figure 1). Click here for file
