# Supplementary material for: The ancestral activation promiscuity of ADP-glucose pyrophosphorylases from oxygenic photosynthetic organisms
Source: BMC Evol Biol. 2013 Feb 21;13:51. doi: 10.1186/1471-2148-13-51 (PMC3585822; doi:10.1186/1471-2148-13-51)
Supplement: Additional file 1: Figure S1 — Sequence alignment used for the phylogenetic tree. The alignment was initially performed with the ClustalW server and manually refined to introduce insertions and deletions in loop regions (based in the crystal structure of the potato tuber ADP-Glc PPase), as described under “Methods”. During the manual refinement of the alignment, 10 sequences were removed prior to tree reconstruction. Sequences 83, 85, 86, 93, 202, and 205 were removed due to their similarity with the A. thaliana aps2, which has no detectable activity in vitro[6]. Sequences 65, 78, and 79 were removed because they have an insertion in a region predicted as a β-sheet and the sequences have not been subjected to the NCBI final revision. Sequence 40 was removed because its C-terminal region was incomplete. Residues are colored based on their chemical properties. [file 1471-2148-13-51-S1.pdf]

Figure S1

```
             10        20        30        40        50        60        70        80        90       100
    ....|....|....|....|....|....|....|....|....|....|....|....|....|....|....|....|....|....|....|....|
1   SRS--V--LGIILG--GGAGTRLYPLTKKRAKPAVPLGANYRL--IDIPVSNCLN--SNI--SKIYVLT--QFNS--ASLNRHLSRA--YASNMGGYKNE
2   SRS--V--LGIILG--GGAGTRLYPLTKKRAKPAVPLGANYRL--IDIPVSNCLN--SNI--SKIYVLT--QFNS--ASLNRHLSRA--YASNMGGYKNE
3   SRS--V--LGIILG--GGAGTRLYPLTKKRAKPAVPLGANYRL--IDIPVSNCLN--SNI--SKIYVLT--QFNS--ASLNRHLSRA--YASNMGGYKNE
4   SRS--V--LGIILG--GGAGTRLYPLTKKRAKPAVPLGANYRL--IDIPVSNCLN--SNI--SKIYVLT--QFNS--ASLNRHLSRA--YASNMGGYKNE
5   SSS--V--LGIILG--GGAGTRLYPLTKKRAKPAVPLGANYRL--IDIPVSNCLN--SNI--SKIYVLT--QFNS--ASLNRHLSRA--YASNMGGYKNE
6   SSS--V--LGIILG--GGAGTRLYPLTKKRAKPAVPLGANYRL--IDIPVSNCLN--SNI--SKIYVLT--QFNS--ASLNRHLSRA--YASNMGGYKNE
7   SRS--V--LGIILG--GGAGTRLYPLTKKRAKPAVPLGANYRL--IDIPVSNCLN--SNI--SKIYVLT--QFNS--ASLNRHLSRA--YASNLGGYKNE
8   SRS--V--LGIILG--GGAGTRLYPLTKKRAKPAVPLGANYRL--IDIPVSNCLN--SNI--SKIYVLT--QFNS--ASLNRHLSRA--YASNMGGYKNE
9   SRS--V--LGIILG--GGAGTRLYPLTKKRAKPAVPLGANYRL--IDIPVSNCLN--SNV--SKIYVLT--QFNS--ASLNRHLSRA--YASNMGGYKNE
10  SRS--V--LGIILG--GGAGTRLYPLTKKRAKPAVPLGANYRL--IDIPVSNCLN--SNV--SKIYVLT--QFNS--ASLNRHLSRA--YASNMGGYKNE
11  SRS--V--LGIILG--GGAGTRLYPLTKKRAKPAVPLGANYRL--IDIPVSNCLN--SNI--SKIYVLT--QFNS--ASLNRHLSRA--YASNMGGYKNE
12  SRS--V--LGIILG--GGAGTRLYPLTKKRAKPAVPLGANYRL--IDIPVSNCLN--SNI--SKIYVLT--QFNS--ASLNRHLSRA--YASNMGGYKNE
13  SRS--V--LGIILG--GGAGTRLYPLTKKRAKPAVPLGANYRL--IDIPVSNCLN--SNI--SKIYVLT--QFNS--ASLNRHLSRA--YASNMGGYKNE
14  SRS--V--LGIILG--GGAGTRLYPLTKKRANRAVPLGANYRL--IDIPVSNCLN--SNI--SKIYVLT--QFNS--ASLNRHLSRA--YASNMGGYKNE
15  SRS--V--LGIILG--GGAGTRLYPLTKKRAKPAVPLGANYRL--IDIPVSNCLN--SNI--SKIYVLT--QFNS--ASLNRHLSRA--YASNMGGYKNE
16  SRS--V--LGIILG--GGAGTRLYPLTKKRAKPAVPLGANYRL--IDIPVSNCLN--SNI--SKIYVLT--QFNS--ASLNRHLSRA--YASNLGGYKNE
17  SRS--V--LGIILG--GGAGTRLYPLTKKRAKPAVPLGANYRL--IDIPVSNCLN--SNI--SKIYVLT--QFNS--ASLNRHLSRA--YASNLGGYKNE
18  SRS--V--LGIILG--GGAGTRLYPLTKKRAKPAVPLGANYRL--IDIPVSNCLN--SNV--SKIYVLT--QFNS--ASLNRHLSRA--YASNMGGYKNE
19  SRS--V--LGIILG--GGAGTRLYPLTKKRAKPAVPLGANYRL--IDIPVSNCLN--SNI--SKIYVLT--QFNS--ASLNRHLSRA--YASNLGGYKNE
20  SRS--V--LGIILG--GGAGTRLYPLTKKRAKPAVPLGANYRL--IDIPVSNCLN--SNI--SKIYVLT--QFNS--ASLNRHLSRA--YASNMGGYKNE
21  SRS--V--LGIILG--GGAGTRLYPLTKKRAKAAVPLGANYRK--IDIPVSNCLN--SNI--SKIYVLT--QFNS--ASLNRHLSRA--YA-VIWWLQNE
22  SRR--V--LGIILG--GGAGTRLYPLTKKRAKPAVPLGANYRL--IDIPVSNCLN--SNI--SKIYVLT--QFNS--ASLNRHLSRA--YASNMGGYKNE
23  SRS--V--LGIILG--GGAGTRLYPLTKKRAKPAVPLGANYRL--IDIPVSNCLN--SNI--SKIYVLT--QFNS--ASLNRHLSRA--YASNMGGYKNE
24  SRS--V--LGIILG--GDGRTRLYPLTKKRAKPAVPLGANYRL--IDIPVSNCLN--SNV--SKIYVLT--QFNS--ASLNRHLSRA--YASNMGGYKNE
25  SRS--V--LGIILG--GGAGTRLYPLTKKRAKPAVPLGANYRL--IDIPVSNCLN--SNI--SKIYVLT--QFNS--ASLIRHLSRA--YASNMGGYKNE
26  SRS--V--LGIILG--GGAGTRLYPLTKKRAKPAVPLGANYRL--IDIPVSNCLN--SNV--SKIYVLT--QFNS--ASLIRHLSRA--YASNMGGYKNE
27  NFS--E--LAFFLE---WSGTRLYLLRKKRAKPAVPIGANYRL--NDIPVSNCLN--SNV--SKIYVLT--QFNS--AFLNRHLSRA--YASNMGGYKNE
28  SRS--V--LGIILG--SGAGTKLYPLTKKRAKPAVPFGANYRL--IDIPVSNCLN--SNI--SKIYVLT--QFNS--ASLNRHLSRA--YASNMGGYKNE
29  SKS--V--LGIILG--GGAGTRLYPLTKKRAKPAVPLGANYRL--IDIPVSNCLN--SNV--SKIYVLT--QFNS--ASLNRHLSRA--YASNMGGYKNE
30  SRS--V--LGIILG--GGAGTRLYPLTKKRAKPAVPLGANYRL--IDIPVSNCLN--SNI--SKIYVLT--QFNS--ASLNRHLSRA--YASNLGGYKNE
31  SRS--V--LGIILG--GGAGTRLYPLTKKRAKPAVPLGANYRL--IDIPVSNCLN--SNI--SKIYVLT--QFNS--ASLNRHLSRA--YASNLGGYKNE
32  STS--V--LGIILG--GGAGTRLYPLTKKRAKPAVPLGANYRL--IDIPVSNCLN--SNV--SKIYVLT--QFNS--ASLNRHLSRA--YGNNIAGYKNE
33  HDS--V--LGIILG--GGAGTRLYPLTKKRAKPAVPLGANYRL--IDIPVSNCLN--SNI--SKIYVLT--QFNS--ASLNRHLSRA--YGSNIGGYKNE
34  STS--V--LGIILG--GGAGTRLYPLTKKRAKPAVPLGANYRL--IDIPVSNCLN--SNI--SKIYVLT--QFNS--ASLNRHLSRA--YGSNIGGYKNE
35  HDS--V--LGIILG--GGAGTRLYPLTKKRAKPAVPLGANYRL--IDIPVSNCLN--SNI--SKIYVLT--QFNS--ASLNRHLSRA--YGSNIGGYKNE
36  STS--V--LGIILG--GGAGTRLYPLTKKRAKPAVPLGANYRL--IDIPVSNCLN--SNI--SKIYVLT--QFNS--ASLNRHLSRA--YGNNIGGYKND
37  DES--V--LGIILG--GGAGTRLYPLTKKRAKPAVPLGANYRL--IDIPVSNCLN--SNI--SKIYVLT--QFNS--ASLNRHLSRA--YGNNIGGYKNE
38  STS--V--LGIILG--GGAGTRLYPLTKKRAKPAVPLGANYRL--IDIPVSNCLN--SNV--SKIYVLT--QFNS--ASLNRHLSRA--YGNNIGGYKNE
39  STS--V--LGIILG--GGAGTRLYPLTKKRAKPAVPLGANYRL--IDIPVSNCLN--SNI--SKIYVLT--QFNS--ASLNRHLSRA--YGNNIGGYKNE
41  NDS--V--LGIILG--GGAGTRLYPLTKKRAKPAVPLGANYRL--IDIPVSNCLN--SNI--SKIYVLT--QFNS--ASLNRHLSRA--YGSNIGGYKNE
42  STS--V--LGIILG--GGAGTRLYPLTKKRAKPAVPLGANYRL--IDIPVSNCLN--SNI--SKIYVLT--QFNS--ASLNRHLSRA--YGSNIGGYKNE
43  STS--V--LGIILG--GGAGTRLYPLTKKRAKPAVPLRANYRL--IDIPVSNCLN--SNV--SKIYVLT--QFNS--ASLNRHLSRA--YGNNIAGYKNE
44  STS--V--LGIILG--GGAGTRLYPLTKKRAKPAVPLGANYRL--IDIPVSNCLN--SNV--SKIYVLT--QFNS--ASLNRHLSRA--YGNNIGGYKND
45  IDS--V--LGIILG--GGAGTRLYPLTKKRAKPAVPLGANYRL--IDIPVSNCLN--SNI--SKIYVLT--QFNS--ASLNRHLSRA--YGSNIGGYXNE
46  STS--V--LGIILG--GGAGTRLYPLTKKRAKPAVPLGANYRL--IDIPVSNCLN--SNI--SKIYVLT--QFNS--ASLNRHLSRA--YGSNIGGYKNE
47  STS--V--LGIILG--GGAGTRLYPLTKKRAKPAVPLGANYRL--IDIPVSNCLN--SNV--SKIYVLT--QFNS--ASLNRHLSRA--YGNNIGGYKND
48  DDS--V--LGIILG--GGAGTRLYPLTKKRAKPAVPLGANYRL--IDIPVSNCLN--SNI--SKIYVRT--QFNS--ASLNRHLSRA--YGSNIGGYKNE
49  PKD--V--AAVILG--GGEGTKLFPLTSRTATPAVPVGGCYRL--IDIPMSNCIN--SAI--NKIFVLT--QYNS--APLNRHIART--Y-FGNGVSFGD
50  PKA--V--ASVILG--GGVGTRLFPLTSRRAKPAVPIGGCYRL--IDVPMSNCIN--SGI--RKIFILT--QFNS--FSLNRHLA-T--YNFGNGVGFGD
51  ART--V--VAIILG--GGAGTRLFPLTKRRAKPAVPMGGAYRL--IDVPMSNCIN--SGI--NKVYILT--QFNS--ASLNRHIARA--YNFGNGVTFES
52  PRT--V--ASIILG--GGAGTRLFPLTKRRAKPAVPIGGAYRL--IDVPMSNCIN--SGI--NKVYILT--QYNS--ASLNRHLARA--YNS-NGLGFGD
53  PKN--V--ASIILG--GGAGTRLFPLTSKRAKPAVPIGGCYRL--IDIPMSNCIN--SGI--RKIFILT--QFNS--FSLNRHLSRT--YNFGNGVNFGD
54  PKN--V--AAIILG--GGDGAKLFPLTKRAATPAVPVGGCYRM--IDIPMSNCIN--SCI--NKIFVLT--QFNS--ASLNRHLART--Y-FGNGINFGD
55  PQN--V--AAIILG--GGNGAKLFPLTMRAATPAVPVGGCYRL--IDIPMSNCIN--SCI--NKIFVLT--QFNS--ASLNRHLART--Y-FGNGINFGG
56  PKD--V--AAVILG--GGEGTKLFPLTSRTATPAVPVGGCYRL--IDIPMSNCIN--SAI--NKIFVLT--QYNS--AALNRHIART--Y-FGNGVSFGD
57  PKA--V--ASVILG--GGVGTRLFPLTSRRAKPAVPIGGCYRL--IDVPMSNCIN--SGI--RKIFILT--QFNS--FSLNRHLART--YNFGNGVGFGD
58  ART--V--VAIILG--GGGGTRLFPLTKRRAKPAVPIGGAYRL--IDVPMSNCIN--SGI--NKVYILT--QFNS--ASLNRHIARA--YNFGNGVTFGD
59  PKD--V--AAVILG--GGEGTKLFPLTSRTATPAVPVGGCYRL--IDIPMSNCIN--SAI--NKIFVLT--QYNS--AALNRHIART--Y-FGNGVSFGD
60  PKA--V--ASVILG--GGVGTRLFPLTSRRAKPAVPIGGCYRV--IDVPMSNCIN--SGI--RKIFILT--QFNS--FSLNRHLART--YNFGNGVGFGD
61  SRT--V--ASIILG--GGAGTRLFPLTKRRAKPAVPIGGAYRL--IDVPMSNCIN--SGI--NKVYILT--QYNS--ASLNRHLARA--YNS-NGVGFGD
62  PKN--V--ASIILG--GGAGTRLFPLTSKRAKPAVPIGGCYRL--IDIPMSNCIN--SGI--RKIFILT--QFNS--FSLNRHLSCT--YNFGNGVNFGD
63  PKN--V--AAIILG--GGDGAKLFPLTKRAATPAVPVGGCYRM--IDIPMSNCIN--SSI--NKIFVLT--QFNS--ASLNRHLART--Y-FGNGINFGD
64  PQN--V--AAIILG--GGNGAKLFPLTMRAATPAVPVGGCYRL--IDIPMSNCIN--SCI--NKIFVLT--QFNS--ASLNRHLART--Y-FGNGINFGG
66  PKT--V--ASIILG--GGAGTRLFPLTQRRAKPAVPFGGCYRL--VDIPMSNCIN--SGI--NKIYVLT--QFNS--QSLNRHIART--YNWGGCINFGG
67  PRT--V--LAVILG--GGAGTRLFPLTKRRAKPAVPIGGAYRL--IDVPMSNCIN--SGI--NKVYILT--QFNS--ASLNRHIARA--YNSGNGVTFGD
68  PEN--V--AAIILG--GGAGTRLFPLTSTRAKQAVPIAGCYRL--IDIPMSNCIN--SGI--RKVYVLT--QFNS--FSLNGHLSRT--YNFGNGVNFGG
69  PEN--V--VAIILG--GGAGTRLFPLTSTRAKQAVPIAGCYRL--IDIPMSNCIN--SGI--RKVYVLT--QFNS--FSLNGHLSRT--YNFGNGVNFGG
70  PKN--V--VSIILG--GGPGIQLFPLTKRAATPAVPVGGCYRL--IDIPMSNCIN--SGL--NKIFVLT--QFNS--ASLNRHISRT--Y-FGNGINFGD
71  PKN--V--VSVILG--GGPGIQLFPLTKRAATPAVPVGGCYRL--IDIPMSNCIN--SGL--NKIFVLT--QFNS--ASLNRHISRT--Y-FGNGINFGD
72  PRT--V--LAVILG--GGAGTRLFPLTKRRAKPAVPIGGAYRL--IDVPMSNCIN--SGI--NKVYILT--QFNS--ASLNRHIARA--YNSGNGVTFGD
```

```
73   PKS--V--ASIILG--GGAGTRLFPLTGRRAKPAVPIGGCYRL--IDIPMSNCIN--SGI--RKIFILT--QFNS--FSLNRHLSRA--YSFGNGMTFGD
74   PKN--V--VSIILG--GGPGTQLFPLTKRAATPAVPVGGCYRL--IDIPMSNCLN--SGI--NKIFVLT--QFNS--ASLNRHIART--Y-FGNGINFGD
75   PKS--V--ASIILG--GGAGTRLFPLTGRRAKPAVPIGGCYRL--IDIPMSNCIN--SGI--RKIFILT--QFNS--FSLNRHLSRA--YSFGNGITFGD
76   PKN--V--ISIILG--GGPGTQLFPLTKRAATPAVPVGGCYRL--IDIPMSNCLN--SGI--NKIFVLT--QFNS--ASLNRHIART--Y-FGNGINFGD
77   PKT--V--ASIILG--GGAGTRLFPLTQRRAKPAVPFGGCYRL--VDIPMSNCIN--SGI--NKIYVLT--QFNS--QSLNRHIAQT--YNLGGCINFGG
80   PKN--V--ASIVLG--GGPGVQLFPLTKRAATPAVPVGGCYRL--IDIPMSNCIN--SGI--NKIFVLT--QFNS--ASLNRHIART--Y-FGNGINFGD
81   PKT--V--AAIILG--GGAGTRLFPLTRRRAKPAVPIGGCYRL--IDVPMSNCIN--SGI--NKIYILT--QFNS--QSLNRHIART--YNQGNGVDFGD
82   PKN--V--ASIILG--GGAGTQLFPLTRRAATPAVPLGGCYRL--IDIPMSNCIN--SGI--NKIFVLT--QFNS--TSLNRHLART--Y-FGNGIIFGD
84   PRT--V--VAVILG--GGAGTRLFPLTKRRAKPAVPIGGSYRL--IDVPMSNCIN--SGI--NKVYILT--QYNS--ASLNRHLARA--YNLGNGVSFGD
87   PRT--V--VAIILG--GGAGTRLFPLTKRRAKPAVPIGGAYRL--IDVPMSNCIN--SGI--NKVYILT--QFNS--ASLNRHLARA--YNFGNGINFGD
88   PKT--V--ASIILG--GGAGTRLFPLTRTRAKPAVPIGGCYRL--IDVPMSNCIN--SGI--NKIYILT--QFNS--QSLNRHIART--YNSGNGVNFGD
89   PKN--V--ASIILG--GGAGTQLFPLTRRAATPAVPVGGCYKL--IDIPMSNCIN--SGI--NKIFVLT--QFNS--ASLNRHLART--Y-FGNGINFGD
90   PKK--V--ASIILG--GGAGTRLFPLTSKRAKPAVPIGGCYRL--IDIPMSNCIN--SGI--RKIFIMT--QFNS--FSLNRHLART--YNFGNGVNFGD
91   PSS--V--AAIILG--GGAGTRLFPLTSRRAKPAVPIGGCYRL--IDVPMSNCIN--SGI--RKIFILT--QFNS--ASLNRHIARI--YNFGNGVNFGD
92   PKT--V--ASIILG--GGAGTRLFPLTKSRAKPAVPIGGCYRL--IDVPMSNCIN--SGI--NKIYILT--QFNS--QSLNRHIART--YNLGSGVNFGD
94   PRT--V--VAVILG--GGAGTRLFPLTKRRAKPAVPIGGSYRL--IDVPMSNCIN--SGI--NKVYILT--QFNS--ASLNRHLARA--YNFGHGVNFGD
95   PKN--V--ASIILG--GGAGTQLFPLTIRQATPAVPVGGCYRL--IDIPMSNCIN--SNI--NKIFILT--QFNS--ASLNRHIART--Y-FGNGVNFGD
96   PGN--V--ASIILG--GGAGTRLFPLTSKRAKPAVPIGGCYRL--IDIPMSNCIN--SGI--KKIFILT--QFNS--FSLNRHIART--YNLGNGVSFGD
97   PKN--V--ASIILG--GGAGTQLFPLTRRAATPAVPVGGCYRL--IDIPMSNCIN--SGI--NKIFVLT--QFNS--ASLNRHLAHT--Y-FGNGINFGD
98   PSN--V--ASIILG--GGAGTRLFPLTSRRAKPAVPIGGCYRL--IDIPMSNCIN--SGI--KKIFILT--QFNS--FSLNRHLART--YNFGNGVSFGD
99   SKT--V--VAVILG--GGAGTRLFPLTRRRAKPAVPIGGAYRL--IDVPMSNCIN--SGI--NKVYILT--QFNS--QSLNRHLSRA--YDFSNGVAIGD
100  ANR--V--SAIILG--GGTGSQLFPLTSTRATPAVPVGGCYRL--IDIPMSNCFN--SGI--NKIFVMS--QFNS--TSLNRHIHRT--YLE-GGINFAD
101  PNE--V--AAVILG--GGTGTQLFPLTSTRATPAVPIGGCYRL--IDIPMSNCFN--SGI--NKIFVMT--QFNS--ASLNRHIHRT--YLG-GGINFTD
102  PDT--V--ASIILG--GGAGTRLFPLTRTRAKPAVPVGGCYRL--IDIPMSNCIN--SKI--NKIYVLT--QFNS--QSLNRHIART--YNFGEGVGFSG
103  PNE--V--AAVILG--GGTGTQLFPLTSTRATPAVPIGGCYRL--IDIPMSNCFN--SGI--NKIFVMT--QFNS--ASLNRHIHRT--YLG-GGINFTD
104  PDT--V--ASIILG--GGAGTRLFPLTRTRAKPAVPVGGCYRL--IDIPMSNCIN--SKI--NKIYVLT--QFNS--QSLNRHIART--YNFGEGVGFGN
105  ART--V--VAVILG--GGAGTRLFPLTKRRAKPAVPIGGAYRL--IDVPMSNCIN--SGI--NKVYVLT--QFNS--ASLNRHLSRA--YNFSNGVGFGD
106  SKT--V--VAVILG--GGAGTRLFPLTKRRAKPAVPIGGAYRL--IDVPMSNCIN--SGI--NKVYILT--QFNS--ASLNRHLSRA--YNFSNGVAFGD
107  PNE--V--AAVILG--GGTGTQLFPLTSTRATPAVPIGGCYRL--IDIPMSNCFN--SGI--NKIFIMT--QFNS--ASLNRHIHRT--YLG-GGINFTD
108  PDT--V--ASIILG--GGAGTRLFPLTRTRAKPAVPVGGCYRL--IDIPMSNCIN--SKI--NKIYVLT--QFNS--QSLNRHIART--YNIGEGVGFGD
109  ASH--V--SAVILG--GGTGVQLFPLTSTRATPAVPVGGCYRL--IDIPMSNCFN--SGI--NKIFVMT--QFNS--ASLNRHIHHT--YLG-GGINFTD
110  PNE--V--AAVILG--GGTGTQLFPLTSTRATPAVPIGGCYRL--IDIPMSNCFN--SGI--NKIFVMT--QFNS--ASLNRHIHRT--YLG-GGINFTD
111  ANH--V--SAIILG--GGTGSQLFPLTSTRATPAVPVGGCYRL--IDIPMSNCFN--SGI--NKIFVMT--QFNS--TSLNRHIHRT--YLG-GEINFAD
112  SRT--V--VAVILG--GGAGTRLFPLTKRRAKPAVPIGGAYRL--IDVPMSNCIN--SGI--NKVYILT--QFNS--QSLNRHLSRA--YDCTNGVAFGD
113  PNE--V--AAVILG--GGTGTQLFPLTSTRATPAVPIGGCYRL--IDIPMSNCFN--SGI--NKIFVMT--QFNS--ASLNRHIHRT--YLG-GGINFTD
114  PRT--V--VAVILG--GGAGTRLFPLTKRRAKPAVPIGGAYRL--IDVPMSNCIN--SGI--NKVYVLT--QFNS--ASLNRHLFRA--YNFSNGVGFGD
115  RPS--V--AAVILG--GGTGTQLFPLTSTRATPAVPIGGCYRL--IDIPMSNCFN--SGI--NKIFVMT--QFNS--ASLNRHIHRT--YLG-GGINFTD
116  PRT--V--VAVILG--GGAGTRLFPLTRQRAKPAVPIGGAYRL--IDVPMSNCIN--SGI--NKVYILT--QFNS--ASLNRHLLRA--YNFSNGIGFGD
117  PNE--V--AAVILG--GGTGTQLFPLTSTRATPAVPIGGCYRL--IDIPMSNCFN--SGI--NKIFVMT--QFNS--ASLNRHIHRT--YLG-GGINFTD
118  PRT--V--VAVILG--GGAGTRLFPLTKRRAKPAVPIGGAYRL--IDVPMSNCIN--SGI--NKVYVLT--QFNS--ASLNRHLSRA--YNFSNGVGFGD
119  PNE--V--AAVILG--GGTGTQLFPLTSTRATPAVPIGGCYRL--IDIPMSNCFN--SGI--NKIFVMT--QFNS--ASLNRHIHRT--YLG-GGINFTD
120  PNE--V--AAVILG--GGTGTQLFPLTSTRATPAVPIGGCYRL--IDIPMSNCFN--SGI--NKIFVMT--QFNS--ASLNRHIHRT--YLG-GGINFTD
121  MKR--V--LAIILG--GGAGTRLYPLTKMRAKPAVPLAGKYRL--IDIPISNCIN--SNI--TKMYVLT--QFNS--ASLNRHLSQT--YDLSA--GFGQ
122  MKQ--V--LAVILG--GGAGTRLYPLTKMRAKPAVPLAGKYRL--IDIPISNCIN--SEI--LKIYILT--QFNS--ASLNRHIART--YNFS---GFTD
123  MKK--V--LAIILG--GGAGTRLYPLTKMRAKPAVPVAGKYRL--IDIPVSNCIN--SEI--FKIYVLT--QFNS--ASLNRHIART--YNFT---GFNE
124  MKK--V--LAIILG--GGAGTRLYPLTKLRAKPAVPLAGKYRL--IDIPVSNCIN--AEI--LKIYVLT--QFNS--ASLNRHLTRT--YNFT---GFHD
125  MNN--V--LSIILG--GGAGTRLYPLTKTRAKPAVPLAGKHRL--IDIPISNCIN--SSI--HKIYVLT--QFNS--ASLNQHISRS--YNFS---GFQQ
126  MKR--V--LAIILG--GGAGTRLYPLTKMRAKPAVPLAGKYRL--IDIPISNCIN--SGI--NKMYVLT--QFNS--ASLNRHLTQS--YNLSS--GFGQ
127  MKR--V--LAIILG--GGAGTRLYPLTKMRAKPAVPLAGKYRL--IDIPISNCIN--SDI--NKMYVLT--QFNS--ASLNRHLTQS--YNLSS--GFGQ
128  MKR--V--LAIILG--GGAGTRLYPLTKMRAKPAVPLAGKYRL--IDIPISNCIN--SGI--NKIYVLT--QFNS--ASLNRHLTQS--YNLSA--GFGQ
129  MKK--V--LSIILG--GGAGTRLYPLTKLRAKPAVPLAGKYRL--IDIPISNCIN--SEI--LKIYVLT--QFNS--ASLNRHISRA--YNFS---GFTD
130  MKR--V--LAIILG--GGAGTRLYPLTKMRAKPAVPLAGKYRL--IDIPISNCIN--SSI--TKMYVLT--QFNS--ASLNRHLSQT--YDLSA--GFGQ
131  MKR--V--LAIILG--GGAGTRLYPLTKMRAKPAVPLAGKYRL--IDIPISNCIN--SDI--HKMYVMT--QFNS--ASLNRHLSQT--FNLSN--SFGG
132  MKR--V--LAIILG--GGAGTRLYPLTKMRAKPAVPLAGKYRL--IDIPISNCIN--SNI--NKMYVLT--QFNS--ASLNRHLSQT--YNLSA--GFGQ
133  MRQ--V--TAIILG--GGRGTRLYPLTKRRAKPAVPIGGKYRL--IDIPVSNCIN--SGI--QHIYILT--QFNS--ASLNRHVSQT--YQFS---RFSD
134  MKR--V--LAIILG--GGKGSRLYPLTKMRAKPAVPLAGKYRL--IDIPISNCIN--SGI--NKMYVLT--QFNS--ASLNRHIGRT--YNLSA--PFGQ
135  MKR--V--LAIILG--GGKGSRLYPLTKMRAKPAVPLAGKYRL--IDIPISNCIN--SNI--TKMYVLT--QFNS--ASLNRHLAQT--YNLSS--PFAQ
136  MKR--V--LAIILG--GGAGTRLYPLTKRRAKPAVPLAGKYRL--IDIPVSNCIN--SEI--HNIYVLT--QFNS--ASLNRHIART--YTFP---GLTG
137  MKK--V--LAIILG--GGAGTRLYPLTKLRAKPAVPLAGKYRL--IDIPLSNCIN--SEI--LKIYVLT--QFNS--ASLNRHLTRT--YNFT---GFSD
138  MKK--V--LSIILG--GGAGTRLYPLTKLRAKPAVPVAGKYRL--IDIPVSNCIN--SEI--FKIYVLT--QFNS--ASLNRHIART--YNFT---GFNE
139  MKR--V--LAIILG--GGAGTRLYPLTKMRAKPAVPLAGKYRL--IDIPISNCIN--SNI--NKMYVLT--QFNS--ASLNRHLGQS--YNLSA--AFGQ
140  MRD--V--LAIILG--GGRGTRLYPLTKRRAKPAVPLAGKYRL--IDIPVSNCIN--SDI--EKIYVLT--QFNS--ASLNRHIVNT--YRLS---PFTG
141  MRD--V--LAIILG--GGRGTRLYPLTKRRAKPAVPLAGKYRL--IDIPVSNCIN--SDI--DKIYVLT--QFNS--ASLNRHIINT--YRMS---PFTG
142  MKR--V--LAIILG--GGAGTRLYPLTKMRAKPAVPLAGKYRL--IDIPISNCIN--SSI--NKMYVLT--QFNS--ASLNRHLGQS--YNLSA--AFGQ
143  MKR--V--LAIILG--GGAGTRLYPLTKMRAKPAVPLAGKYRL--IDIPISNCIN--SNI--NKMYVMT--QFNS--ASLNRHLSQT--FNLSA--SFGQ
144  MKK--V--LAIILG--GGAGTRLYPLTKLRAKPAVPVAGKYRL--IDIPVSNCIN--SEI--FKIYVLT--QFNS--ASLNRHIART--YNFS---GFSE
145  MKR--V--LAIILG--GGKGSRLYPLTKMRAKPAVPLAGKYRL--IDIPISNCIN--SNI--HKMYVLT--QFNS--ASLNRHISQT--YNLSS--PFAQ
146  MKR--V--LAIILG--GGKGSRLYPLTKMRAKPAVPLAGKYRL--IDIPISNCIN--SGI--KKMYVLT--QFNS--ASLNRHIGRT--YNLNG--PFGQ
147  MKR--V--LAIILG--GGKGSRLYPLTKMRAKPAVPLAGKYRL--IDIPISNCIN--SDI--SKMYVLT--QFNS--ASLNRHIAQT--YNLSG--PFGQ
148  MKR--V--LAIILG--GGKGSRLYPLTKMRAKPAVPLAGKYRL--IDIPISNCIN--SGI--EKMYVLT--QFNS--ASLNRHIGRT--YNLNG--PFGQ
149  MKR--V--LAIILG--GGKGSRLYPLTKMRAKPAVPLAGKYRL--IDIPISNCIN--SGI--NKMYVLT--QFNS--ASLNRHIGRT--YNLSA--PFGQ
150  MKR--V--LAIILG--GGAGTRLQPLTKMRAKPAVPLAGKYRL--IDIPISNCIN--SSI--NKMYVLT--QFNS--ASLNRHLSQT--YNLNA--GFGQ
151  MKR--V--LAIILG--GGAGTRLYPLTKLRAKPAVPLAGKYRL--IDIPVSNCIN--SEI--TKIYVLT--QFNS--ASLNRHLSRT--YNFT---GFND
152  MKR--V--LAIILG--GGKGSRLYPLTKMRAKPAVPLAGKYRL--IDIPISNCIN--SGI--EKMYVLT--QFNS--ASLNRHIGRT--YNLNG--PFGQ
153  VKR--V--LAIILG--GGAGTRLYPLTKLRAKPAVPLAGKYRL--IDIPVSNCIN--SEI--VKIYVLT--QFNS--ASLNRHISRA--YNFS---GFQE
154  MKK--V--LAIILG--GGAGTRLYPLTKLRAKPAVPLAGKYRL--IDIPLSNCIN--SEI--LKIYVLT--QFNS--ASLNRHLTRT--YNFT---GFSD
155  MKN--V--LAIILG--GGAGSRLYPLTKQRAKPAVPLAGKYRL--IDIPVSNCIN--ADI--NKIYVLT--QFNS--ASLNRHLSQT--YNLSS--GFGN
```

```
156  MKK--V--LAIILG--GGAGTRLYPLTKQRAKPAVPLAGKYRL--IDIPVSNCIN--SEI--THVYVLT--QFNS--ASLNRHIART--YNFS---GFSD
157  MKN--V--LAIILG--GGAGSRLYPLTKQRAKPAVPLAGKYRL--IDIPVSNCIN--ADI--NKIYVLT--QFNS--ASLNRHLSQT--YNLSS--GFGN
158  MKR--V--LAIILG--GGAGTRLYPLTKMRAKPAVPLAGKYRL--IDIPISNCIN--SDI--NKMYVMT--QFNS--ASLNRHLSQT--YNLSN--SFGG
159  MKR--V--LAIILG--GGAGTRLYPLTKMRAKPAVPLAGKYRL--IDIPISNCIN--SDI--HKMYVMT--QFNS--ASLNRHLSQT--FNLSN--SFGG
160  MKR--V--LSIILG--GGAGTRLYPLTKLRAKPAVPLAGKYRL--IDIPVSNCIN--SDI--NKIYVLT--QFNS--ASLNRHLSRG--YNFS---NFTE
161  MNR--V--LAIVLG--GGAGTRLYPLTKQRAKPAVSLAGKYRL--IDIPMSNCIN--SEI--NKIYVMT--QFNS--ASLNRHISQT--YHFS---SFSD
162  MKN--V--LSIILG--GGAGTRLYPLTKLRAKPAVPLAGKYRL--IDIPISNCIN--SEI--QKIYVLT--QFNS--ASLNRHITRT--YNFS---GFSD
163  MKK--V--LAIILG--GGAGTRLYPLTKLRAKPAVPVAGKYRL--IDIPVSNCIN--SEI--FKIYVLT--QFNS--ASLNRHIART--YNFS---GFSE
164  MNN--V--LSIILG--GGAGTRLYPLTKTRAKPAVPLAGKHRL--IDIPISNCIN--SNL--LKIYVLT--QFNS--ASLNQHISRS--YNFS---GFQQ
165  MKK--V--LAIILG--GGAGTRLYPLTKLRAKPAVPLAGKYRL--IDIPVSNCIN--AEI--LKIYVLT--QFNS--ASLNRHLTRT--YNFT---GFHD
166  MKR--V--LGIILG--GGAGTRLYPLTKLRAKPAVPLAGKYRL--IDIPVSNCIN--SEI--HKIYILT--QFNS--ASLNRHISRT--YNFT---GFTE
167  MKR--V--LAIILG--GGKGSRLYPLTKMRAKPAVPLAGKYRL--IDIPISNCIN--SGI--EKMYVLT--QFNS--ASLNRHIGRT--YNLNG--PFGQ
168  MKR--V--LAIILG--GGKGSRLYPLTKMRAKPAVPLAGKYRL--IDIPISNCIN--SDI--SKMYVLT--QFNS--ASLNRHIAQT--YNLSG--PFGQ
169  MKR--V--LAIILG--GGAGTRLYPLTKMRAKPAVPLAGKYRL--IDIPVSNCIN--SGI--NKIYVLT--QFNS--ASLNRHIAQT--FNLSS--GFDQ
170  MKR--V--LAIILG--GGAGTRLYPLTKMRAKPAVPLAGKYRL--IDIPISNCIN--SNI--NKMYVLT--QFNS--ASLNRHLSQT--YNLSA--GFGQ
171  MKK--V--LAIILG--GGAGTRLYPLTKLRAKPAVPLAGKYRL--IDIPVSNCIN--SQI--DKIYVLT--QFNS--ASLNRHLNRT--YNFT---GFSD
172  MKR--V--LAIILG--GGKGSRLYPLTKMRAKPAVPLAGKYRL--IDIPISNCIN--SGI--EKMYVLT--QFNS--ASLNRHIGRT--YNLNG--PFGQ
173  MKR--V--LAIILG--GGAGTRLYPLTKMRAKPAVPLAGKYRL--IDIPISNCIN--SEI--NKIYVLT--QFNS--ASLNRHLSMS--YNLSA--GFGQ
174  MKK--V--LSIILG--GGAGTRLYPLTKLRAKPAVPLAGKYRL--IDIPVSNCIN--SEI--TKIYVLT--QFNS--ASLNRHLSRT--YNFN---GFND
175  MKK--V--LAIILG--GGAGTRLYPLTKLRAKPAVPVAGKYRL--IDIPVSNCIN--SEI--FKIYVLT--QFNS--ASLNRHIARA--YNFS---GFSD
176  MKK--V--LGIILG--GGAGSRLYPLTKPRAKPAVSLAGKYRL--IDIPVSNCIN--SEI--YKIYVLT--QFNS--ASLNRHITRA--YNFS---GFTE
177  MKK--V--LAIILG--GGAGTRLYPLTKLRAKPAVPLAGKYRL--IDIPVSNCIN--SEI--FKIYVLT--QFNS--ASLNRHIART--YSFA---GFTE
178  MKK--V--LAIILG--GGAGTRLYPLTKLRAKPAVPLAGKYRL--IDIPVSNCIN--AEI--LKIYVLT--QFNS--ASLNRHLTRT--YNFT---GFHD
179  MNN--V--LSIILG--GGAGTRLYPLTKTRAKPAVPLAGKHRL--IDIPISNCIN--SNL--LKIYVLT--QFNS--ASLNQHISRS--YNFS---GFQQ
180  MKR--V--LAIILG--GGAGTRLQPLTKMRAKPAVPLAGKYRL--IDIPISNCIN--SSI--NKMYVLT--QFNS--ASLNRHLSQT--YNLNA--GFGQ
181  MKK--V--LAIILG--GGAGTRLYPLTKLRAKPAVPLAGKYRL--IDIPISNCIN--AKI--QKIYVLT--QFNS--ASLNHHLTHT--YNFG---PFSG
182  MKK--V--LAIILG--GGVGTRLYPLTKLRAKPAVPLAGKYRL--IDIPVSNCIN--SEI--VKIYVLT--QFNS--ASLNRHISRT--YQFS---GFTE
183  MKK--V--LAIILG--GGVGTRLYPLTKLRAKPAVPLAGKYRL--IDIPVSNCIN--SEI--LKIYVLT--QFNS--ASLNRHISRA--YNFS---GFSD
184  MKR--V--LAIILG--GGAGTRLYPLTKLRAKPAVPVAGKYRL--IDIPVSNCIN--SEI--FKIYVLT--QFNS--ASLNRHIARA--YNFS---GFSD
185  MKR--V--LAIILG--GGAGTRLYPLTKLRAKPAVPVAGKYRL--IDIPVSNCIN--SEI--FKIYVLT--QFNS--ASLNRHIARA--YNFS---GFSD
186  VKQ--V--LAVILG--GGAGTRLYPLTKMRAKPAVPLAGKYRL--IDIPISNCIN--SEI--LKIYILT--QFNS--ASLNRHIART--YNFS---GFTD
187  MKK--V--LAIILG--GGAGTRLYPLTKLRAKPAVPLAGKYRL--IDIPVSNCIN--AEI--LKIYVLT--QFNS--ASLNRHLTRT--YNFT---GFSD
188  MKR--V--LAIILG--GGAGTRLYPLTKMRAKPAVPLAGKYRL--IDIPISNCIN--SDI--NKMYVMT--QFNS--ASLNRHLSQT--YNLSN--SFGG
189  SKS--V--AAVILG--GGAGTRLYPLTKSRAKPAVPIGGAYRL--IDVPMSNCLN--SGI--SKMYILT--QFNS--VSLNRHLART--YNFGNGIMYGG
190  TDN--V--LAIILG--GGAGTRLYPLTKKRAKPAVPLGANYRL--IDIPVSNCIN--SDI--NKMYCLT--QFNS--ASLNRHLSQA--YNSNVGSGLRQ
191  SKS--V--AAVILG--GGAGTRLYPLTKSRAKPAVPIGGAYRL--IDVPMSNCLN--SGI--SKMYILT--QFNS--VSLNRHLART--YNFGNGIMYGG
192  TDN--V--LGIILG--GGAGTRLYPLTKTRAKPAVPLGANYRL--IDLPVSNCIN--SDI--NKMYCLT--QFNS--ASLNRHLSQA--YNNNVGSYNRQ
193  GEV--C--SSIILG--GGAGTRLFPLTKSRAKPAVPIGGAYRL--IDVPMSNCIN--SGI--SKIYILT--QFNS--TSLNRHLGRA--YNMGSGVRFGG
194  SKT--V--LGIILG--GGAGTRLYPLTKKRAKPAVPLGANYRL--IDIPVSNCLN--SNV--TKIYCLT--QFNS--ASLNRHLSQA--YNSSVGGYNSR
195  TKT--V--AAVILG--GGAGTRLYPLTKSRAKPAVPIGGAYRL--IDVPMSNCLN--SGI--SKVYILT--QFNS--ASLNRHLART--YNFGNGIMYGG
196  MDN--V--LSIILG--GGAGTRLYPLTKKRAKPAVPLGANYRL--IDIPVSNCIN--SDI--NKVYCLT--QFNS--ASLNRHLAQA--YNTNIGTHTRQ
197  SKT--V--LGIILG--GGAGTRLYPLTKKRAKPAVPLGANYRL--IDIPVSNCLN--SNV--TKIYCLT--QFNS--ASLNRHLSQA--YNSSVGGYNTR
198  TNT--V--LSIILG--GGAGTRLFPLTKQRAKPAVPIGGAYRL--IDVPMSNCIN--SGI--SKIYILT--QFNS--TSLNRHLARA--YNMGSGVRFGG
199  SKT--V--AAVILG--GGAGTRLYPLTKSRAKPAVPIGGAYRL--IDVPMSNCLN--SGI--SKVYILT--QFNS--ASLNRHLART--YNFGNGIMYGG
200  MDN--V--LSIILG--GGAGTRLYPLTKKRAKPAVPLGANYRL--IDIPVSNCIN--SDI--NKVYCLT--QFNS--ASLNRHLSQA--YNTNIGTYTRQ
201  PRT--V--VSLILG--GGAGTRLFPLTNRRAKPAVPIGGAYRL--IDVPMSNCIN--SGI--NKIFILT--QFNS--ASLNRHLART--YNFGNGVNFGD
203  GVS--V--LGIILG--GGAGTRLYPLTKKRAKPAVPLGANYRL--IDIPVSNCIN--SNI--RKIYVLT--QFNS--ASLNRHLSRA--YSSNMGNYKNE
204  GVS--V--LGIILG--GGAGTRLYPLTKKRAKPAVPLGANYRL--IDIPVSNCIN--SNI--RKIYVLT--QFNS--ASLNRHLSRA--YSSNMGNYKNE
206  PKS--V--VSIILG--GGVGTRLFPLTKQRAKPAVPIGGGYRL--IDVPMSNCIN--SGI--NRVFVLT--QFNS--ASLNRHLART--YNF---INAGD
207  PKS--V--VSIILG--GGVGTRLFPLTKQRAKPAVPIGGGYRL--IDVPMSNCIN--SGI--NRVFVLT--QFNS--ASLNRHLART--YNF---INAGE
208  PRT--V--VSLILG--GGAGTRLFPLTNRRAKPAVPIGGAYRL--IDVPMSNCIN--SGI--NKIFILT--QFNS--ASLNRHLART--YNFGNGVNFGD
209  PRT--V--VSLILG--GGAGTRLFPLTNRRAKPAVPIGGAYRL--IDVPMSNCIN--SGI--NKIFILT--QFNS--ASLNRHLART--YNFGNGVNFGD

              110       120       130       140       150       160       170       180       190       200
     ....|....|....|....|....|....|....|....|....|....|....|....|....|....|....|....|....|....|....|....|
1    G---FVEVLAA--QQS--PENPDWFQG--TADAVR--QYL--WLFE--EH----TV--LEYLILAGDHL--YRMDY--EKFIQAHRE--TDA--DITVAA
2    G---LVEVLAA--QQS--PENPDWFQG--TADAVR--QYL--WLFE--EH----TV--LEYLILAGDHL--YRMDY--EKFIQAHRE--TDA--DITVAA
3    G---FVEVLAA--QQS--PENPNWFQG--TADAVR--QYL--WLFE--EH----NV--LEYLILAGDHL--YRMDY--EKFIQAHRE--TDA--DITVAA
4    G---FVEVLAA--QQS--PENPDWFQG--TADAVR--QYL--WLFE--EH----TV--LEYLILAGDHL--YRMDY--EKFIQAHRE--TDA--DITVAA
5    G---FVEVLAA--QQS--PENPNWFQG--TADAVR--QYL--WLFE--EH----NV--LEYLILAGDHL--YRMDY--EKFIQAHRE--TDA--DITVAA
6    G---FVEVLAA--QQS--PENPNWFQV--TADAVR--QYL--WLFE--EH----NV--LEYLILAGDHL--YRMDY--EKFIQAHRE--TDA--DITVAA
7    G---FVEVLAA--QQS--PENPNWFQG--TADAVR--QYL--WLFE--EH----NV--LEYLILAGDHL--YRMDY--EKFIQAHRE--TDA--DITVAA
8    G---FVEVLAA--QQS--PENPNWFQG--TADAVR--QYL--WLFE--EH----NV--LEYLVLAGDHL--YRMDY--ERFIQAHRE--SDA--DITVAA
9    G---FVEVLAA--QQS--PENPNWFQG--TADAVR--QYL--WLFE--EH----NV--LEFLVLAGDHL--YRMDY--EKFIQAHRE--TDA--DITVAA
10   G---FVEVLAA--QQS--PENPNWFQG--TADAVR--QYL--WLFE--EH----NV--LEFLVLAGDHL--YRMDY--EKFIQAHRE--TDA--DITVAA
11   G---FVEVLAA--QQS--PENPNWFQG--TADAVR--QYL--WLFE--EH----NV--LEFLVLAGDHL--YRMDY--ERFIQAHRE--TDA--DITVAA
12   G---FVEVLAA--QQS--PENPNWFQG--TADAVR--QYL--WLFE--EH----NV--LEFLILAGDHL--YRMDY--ERFIQAHRE--TDA--DITVAA
13   G---FVEVLAA--QQS--PENPNWFQG--TADAVR--QYL--WLFE--EH----NV--LEFLVLAGDHL--YRMDY--ERFIQAHRE--TDA--DITVAA
14   G---FVEVLAA--QQS--PENPNWFQG--TADAVR--QYL--WLFE--EH----NV--LEFLVLAGDHL--YRMDY--EKFIQAHRE--TDA--DITVAA
15   G---FVEVLAA--QQS--PENPNWFQG--TADAVR--QYL--WLFE--EH----NV--LEYLILAGDHL--YRMDY--ERFVQAHRE--TDA--DITVAA
16   G---FVEVLAA--QQS--PENPNWFQG--TADAVR--QYL--WLFE--EH----NV--LEYLVLAGDHL--YRMDY--ERFIQAHRE--SDA--DITVAS
17   G---FVEVLAA--QQS--PENPNWFQG--TADAVR--QYL--WLFE--EH----NV--LEYLILAGDHL--YRMDY--EKFIQAHRE--SDA--DITVAA
18   G---FVEGLAA--QQS--PENPNWFQG--TADAVR--QYL--WLFE--EH----NV--LEYLVLAGDHL--YRMDY--ERFIQAHRE--SDA--DITVAA
19   G---FVEVLAA--QQS--PENPNWFQG--TADAVR--QYL--WLFE--EH----NV--LEFLILAGDHL--YRMDY--EKFIQAHRE--TDA--DITVAA
20   G---FVEVLAA--QQS--PENPNWFQG--TADAVR--QYL--WLFE--EQ----NV--LEYLVLAGDHL--YRMDY--ERFIQAHRE--TDA--DITVAA
21   G---FVEVLAA--QQS--PENPNWFQG--TADAVR--QYL--WLFE--EQ----NV--LEYLVLAGDHL--YRMDY--ERFIQAHRE--TDA--DITVAA
22   G---FVEVLAA--QQS--PENPNWFQG--TADAVR--QYL--WLFE--EH----NV--LEFLVLAGDHL--YRMDY--ERFIQAHRE--TDA--DITVAA
```

```
23   G---FVEVLAA--QQS--PENPNWFQG--TADAVR--QYL--WLFE--EH----NV--LEFLVLAGDHL--YRMDY--ERFIQAHRE--TDA--DITVAA
24   G---FVEVLAA--QQS--PENPNWFQG--TADAVR--QCL--WLFE--EH----NV--LEFLVLAGDHL--YRMDY--EKFIQAHRE--TDA--DITVAA
25   G---FVEVLAA--QQS--PENPNWFQG--TADAVR--QYL--WLFE--EH----NV--LEFLVLAGDHL--YRMDY--ERFIQAHRE--TDA--DITVAA
26   G---FVEVLAA--QQS--PENPNWFQG--TADAVR--QYL--WLFE--EH----NV--LEFLVLAGDHL--YRMDY--ERFIQAHRE--TDA--DITVAA
27   G---FVEVFAA--QQS--PENPNWFQG--TADAVR--QYL--WLFE--EH----DV--LEYLVLAGDHL--YRMDY--EKFVQSHRE--TDA--DITVAA
28   G---FVEVLAA--QQS--PENPNWFQG--TADAVR--QYL--WLFE--EH----NV--LEFLILAGDHL--YRMDY--ERFIQAHRE--TDA--DITVAA
29   G---FVEVLAA--QQS--PENPNWFQG--TADAVR--QYL--WLFE--EH----NV--LEYLVLAGDHL--YRMDY--EKFIQVHRE--SDA--DITVAA
30   G---FVEVLAA--QQS--PENPNWFQG--TADAVR--QYL--WLFE--EH----NV--LEYLVLAGDHL--YRMDY--ERFIQAHRE--SDA--DITVAA
31   G---FVEVLAA--QQS--PENPNWFQG--TADAVR--QYL--WLFE--EH----NV--LEYLILAGDHL--YRMDY--EKFIQAHRE--SDA--DITVAA
32   G---FVEVLAA--QQS--PENPNWFQG--TADAVR--QYM--WLFE--EH----NI--MEFLILAGDHL--YRMDY--QKFIQAHRE--TDA--DITVAA
33   G---FVEVLAA--QQS--PDNPNWFQG--TADAVR--QYL--WLFE--EH----NV--MEFLILAGDHL--YRMDY--EKFIQAHRE--TNA--DITVAA
34   G---FVEVLAA--QQS--PDNPNWFQG--TADAVR--QYL--WLFE--EH----NV--MEFLILAGDHL--YRMDY--EKFIQAHRE--TNA--DITVAA
35   G---FVEVLAA--QQS--PDNPNWFQG--TADAVR--QYL--WLFE--EH----NV--MEFLILAGDHL--YRMDY--EKFIQAHRE--TNA--DITVAA
36   G---FVEVLAA--QQS--PDNPNWFQG--TADAVR--QYL--WLFE--EH----NV--MEFLILAGDHL--YRMDY--EKFIQAHRE--TDA--DITVAA
37   G---FVEVLAA--QQS--PDNPNWFQG--TADAVR--QYL--WLFE--EH----NV--MEFLILAGDHL--YRMDY--EKFIQAHRE--TDS--DITVAA
38   G---FVEVLAA--QQS--PENPNWFQG--TADAVR--QYL--WLFE--EH----NV--MEFLILAGDHL--YRMDY--QKFIQAHRE--TNA--DITVAA
39   G---FVEVLAA--QQS--PDNPNWFQG--TADAVR--QYL--WLFE--EH----NV--MEFLILAGDHL--YRMDY--EKFIQAHRE--TDS--DITVAA
41   G---FVEVLAA--QQS--PDNPNWFQG--TADAVR--QYL--WLFE--EH----NV--MEYLILAGDHL--YRMDY--EKFIQAHRE--TDA--DITVAA
42   G---FVEVLAA--QQS--PDNPNWFQG--TADAVR--QYL--WLFE--EH----NV--MEYLILAGDHL--YRMDY--EKFIQAHRE--TDA--DITVAA
43   G---FVEVLAA--QQS--PENPNWFQG--TADAVR--QYM--WLFE--EH----NI--MEFLILAGDHL--YRMDY--QKFIQAHRE--TDA--DITVAA
44   G---FVEVLAA--QQS--PENPNWFQG--TADAVR--QYL--WLFE--EH----NV--MEFLILAGDHL--YRMDY--QKFIQAHRE--TDA--DITVAA
45   G---FVEVLAA--QQS--PDNPDWFQG--TADAVR--QYL--WLFE--EH----NV--MEYLILAGDHL--YRMDY--EKFIQAHRE--TDA--DITVAA
46   G---FVEVLAA--QQS--PDNPDWFQG--TADAVR--QYL--WLFE--EH----NV--MEYLILAGDHL--YRMDY--EKFIQAHRE--TDA--DITVAA
47   G---FVEVLAA--QQS--PESPDWFQG--TADAVR--QYL--WLFE--EH----NV--MEFLILAGDHL--YRMDY--QKFIQAHRE--TDA--DITVAA
48   G---FVEVLAA--QQS--PDNPDWFQG--TADAVR--QYL--WLFE--EH----NV--MEYLILAGDHL--YRMDY--EKFIQAHRE--TDA--DITVAA
49   G---FVEVLAA--TQTPGEAGKKWFQG--TADAVR--KFI--WVFE--DAK-NKNI--ENIVVLSGDHL--YRMDY--MELVQNHID--RNA--DITLSC
50   G---FVEVLAG--TQTPGDGRKMWFQ---AADAVR--EFI--WVFE--NQK-NKNV--EHIIILSGDHL--YRMNY--MDFVQKHID--TNA--DITVSC
51   G---YVEVLAA--TQTPGELGKRWFQG--TAHAVR--QFH--WLFE--DAR-SKDI--EDVLILSGDHL--YRMDY--LHFVQSHRQ--SGA--DITISS
52   G---YVEVLAA--TQTPGESGKRWFQG--TADAVR--QFH--WLFE--DAR-SKDI--EDVLILSGDHL--YRMDY--MDFIQDHRQ--SGA--DISISC
53   G---FVEVLAA--TQTSGDAGKKWFQG--TADAVR--QFI--WVFE--DAK-TKNV--EHVLILSGDHL--YRMDY--MNFVQKHIE--SNA--DITVSC
54   G---FVEVLAA--TQTPGEAGKKWFQG--TADAVR--KFL--WVFE--DAK-NRNI--ENIIILSGDHL--YRMNY--MDFVQHHVD--SKA--DITLSC
55   G---FVEVLAA--TQTPGEAGKKWFQG--TADAVR--KFL--WVFE--DAK-NRNI--ENILILSGDHL--YRMNY--MDFVQSHVD--SNA--DITLSC
56   G---FVEVLAA--TQTPGEAGKKWFQG--TADAVR--KFI--WVFE--DAK-NKNI--ENILVLSGDHL--YRMDY--MELVQNHID--RNA--DITLSC
57   G---FVEVLAA--TQTPGDAGKMWFQG--TADAVR--QFI--WVFE--NQK-NKNV--EHIIILSGDHL--YRMNY--MDFVQKHID--ANA--DITVSC
58   G---YVEVLAA--TQTPGELGKRWFQG--TADAVR--QFH--WLFE--DAR-SKDI--EDVLILSGDHL--YRMDY--LHFVQSHRQ--SGA--DITISS
59   G---FVEVLAA--TQTPGEAGKKWFQG--TADAVR--KFI--WVFE--DAK-NKNI--ENILVLSGDHL--YRMDY--MELVQNHID--RNA--DITLSC
60   G---FVEVLAA--TQTPGDAGKMWFQG--TADAVR--QFI--WVFE--NQK-NKNV--EHIIILSGDHL--YRMNY--MDFVQKHID--ANA--DITVSC
61   G---YVEVLAA--TQTPGESGKRWFQG--TADAVR--QFH--WLFE--DAR-SKDI--EDVLILSGDHL--YRMDY--MDFVQDHRQ--SGA--DISISC
62   G---FVEVLAA--TQTSGDAGKKWFQG--TADAVR--QFI--WVFE--DAK-TKNV--EHVLILSGDHL--YRMDY--MNFVQKHIE--SNA--DITVSC
63   G---FVEVLAA--TQTPGEAGKKWFQG--TADAVR--KFL--WVFE--DAK-NRNI--ENILILSGDHL--YRMNY--MDFVQYHVD--SKA--DITLSC
64   G---FVEVLAA--TQTPGEAGKMWFQG--TADAVR--KFL--WVFE--DAK-NRNI--ENILILSGDHL--YRMNY--MDFVQSHVD--SNA--DITLSC
66   G---FVEVLAA--TQTPGESGKKWFQG--TADAVR--QFL--WLFE--DAD-HKNI--ENILILCGDQL--YRMDY--MEIVQKHIN--SCA--DISVSC
67   G---YVEVLAA--TQTPGEAGKKWFQG--TADAVR--QFH--WLFE--DPR-SKDI--EDVLILSGDHL--YRMDY--MDFVQNHRE--SGA--DITLSC
68   G---FVEVLAA--TKTPGESGNKWFQG--TADAVR--RFI--WVFE--DAK-NKDI--ENILIISGDHL--CRMDY--MKLLEKHIG--TNA--DITVSC
69   G---FVEVLAA--TLTNGEAGNKWFQG--TADAVR--RFS--WVFE--DAK-NKNI--EHILIISGDHL--CRMDY--MKLVEKHIG--TNA--DITVSC
70   G---CVEVLAA--TQTQGETGKNWFQG--TADAVR--QFT--WVFE--DAK-HTNI--ENVLILAGDHL--YRMDY--MDLVQSHVD--RNA--DITVSC
71   G---CVEVLAA--TQTQGEAGNNWFQG--TADAVR--QFT--WVFE--DAK-HANI--ENVLILAGDHL--YRMNY--MDLVQSHVD--RNA--DITVSC
72   G---YVEVLAA--TQTPGEAGKKWFQG--TADAVR--QFH--WLFE--DPR-SKDI--EDVLILSGDHL--YRMDY--MDFVQNHRE--SGA--DITLSC
73   G---FVEVLAA--TQTPGEAGKKWFQG--TADAVR--QFI--WVFE--DAK-NKNV--EHILILSGDHL--YRMDY--MDFVQRHVD--TNA--DITVSC
74   G---IVEVLAA--TQTPGEAGKNWFQG--TADAVR--QFT--WVFE--DAK-NTNV--ENVLILAGDHL--YRMDY--MDLVQSHVD--RNA--DITVSC
75   G---FVEVLAA--TQTPGEAGKKWFQG--TADAVR--QFI--WVFE--DAK-NKNV--EHILILSGDHL--YRMDY--MNFVQRHVD--TNA--DITVSC
76   G---IVEVLAA--TQTPGEAGKNWFQG--TADAVR--QFT--WVFE--DAK-NTNV--ENVLILAGDHL--YRMDY--MDLVQSHVD--RNA--DITVSC
77   G---FVEVLAA--TQTPGESGKKWFQG--TADAVR--QFL--WLFE--DAD-HKNI--ENILILCGDQL--YRMDY--MEIVQKHIN--SCA--DISVSC
80   G---YVEVLAA--TQTPGEAGKNWFQG--TADAVR--QFT--WVFE--DAK-NTNI--ENVIILAGDHL--YRMDY--MDLVQSHID--RNA--DITVSC
81   G---FVEVLAA--TQTPGESGKKWFQG--TADAVR--QFI--WLFE--DAK-LRNI--ENILVLSGDHL--YRMDY--MDFLQKHIE--SGA--DICVSC
82   G---FVEVLAA--TQTPGEAGMKWFQG--TADAVR--QFT--WVFE--DAK-NRNI--ENILVLSGDHL--YRMDY--MDFVQHHID--SNA--DFTISC
84   G---FVEALAA--TQTPGEAGKKWFQG--TADAVR--QFH--WLFE--GPR-SKEI--EDVLILSGDHL--YRMDY--MDFVQNHRQ--GGA--DITLSC
87   G---FVEVLAA--TQTPGEAGKRWFQG--TADAVR--QFH--WLFE--DAR-SKDI--DDVLVLSGDHL--YRMDY--MDFVQNHRQ--SGA--DITISC
88   G---FVEVLAA--TQTPGESGKKWFQG--TADAVR--QFL--WLFE--DAK-HSHI--ENILILSGDHL--YRMDY--MDFLQKHID--SGA--DITVSC
89   G---FVEVLAA--TQTPGEAGMNWFQG--TADAVR--QFT--WVFE--DAK-NRNV--ENILILSGDHL--YRMDY--MDFVQHHVD--SNA--DITISC
90   G---FVEVLAA--TKTPGEAGNKWFQG--TADAVR--QFI--WVFE--DAK-NKNV--ENVLILSGDHL--YRMDY--MEFVQKHID--SGA--DITVSC
91   G---FVEVLAA--TQTPGEAGQKWFQG--TADAVR--QFI--WVFE--DAK-NKNV--EHILILSGDHL--YRMDY--MDFVQKHID--SNA--DITVSC
92   G---FVEVLAA--TQTSGESGKKWFQG--TADAVR--QFI--WLFE--DAR-HRHI--ENILILSGDHL--YRMDY--MEFVQKHID--SDA--DISVSC
94   G---YVEALAA--TQTPGEAGKRWFQG--TADAVR--QFH--WLFE--DQR-SKEI--EDVLILSGDHL--YRMDY--MDFVQNHRQ--SGA--DITISC
95   G---FVEVLAA--TQTPGEAGMKWFEG--TADAVR--KFI--WVFE--DAK-NKNI--ENILILSGDHL--YRMDY--MDLVQNHID--RKA--DITVSC
96   G---FVEVLAA--TQTPGETGKKWFQG--TADAVR--QFI--WVFE--DAR-NKNV--EHVLILSGDHL--YRMNY--MEFVQKHID--TNA--DVTVSC
97   G---FVEVLAA--TQTPGEAGMKWFQG--TADAVR--QFT--WVFE--DAK-NRSI--ENILILSGDHL--YRMDY--MDFVQHHVD--SNA--DITISC
98   G---FVEVLAA--TQTPGEAGKKWFQG--TADAVR--QFI--WMFE--DAR-TKNV--EHVLILSGDHL--YRMNY--MEFVQKHID--TNA--DITVSC
99   G---FVEVLAA--TQRPGTEGKRWFQG--TADAVR--QFD--WLFD--DAK-SKDI--EDVLILSGDHL--YRMDY--MDFVQSHRQ--RGA--GISICC
100  G---SVQVLAA--TQMPEEPAG-WFQG--TADSIR--KFI--WVLE--DYYSHKSI--DNIVILSGDQL--YRMNY--MELVQKHVE--DDA--DITISC
101  G---SVEVLAA--TQMPGEAAG-WFQG--TADAVR--KFI--WVLE--DYYKHKAI--EHILILSGDQL--YRMDY--MELVQKHVD--DNA--DITLSC
102  G---SVEVLAA--TQTAGESGKKWFQG--TADAVR--QFL--WLFE--DAR-LKCI--ENILILSGDHL--YRMDY--MDFVQKHVD--SGA--DISVAC
103  G---SVEVLAA--TQMPGEAAG-WFRG--TADAVR--KFI--WVLE--DYYKHKSI--EHILILSGDQL--YRMDY--MELVQKHVD--DNA--DITLSC
104  G---FVEVLAA--TQTAGESGKRWFQG--TADAVR--QFL--WLFE--DAR-LKRI--ENILILSGDHL--YRMDY--MDFVQKHVD--SGA--DISVAC
105  G---FVEVLAA--TQRPGLEGKRWFQG--TADAVR--QFD--WLFD--DAK-AKDI--EDVIILSGDHL--YRMDY--MDFVQSHRQ--RDA--GISICC
106  G---FVEVLAA--TQTPGSEGKRWFQG--TADAVR--QFD--WLFD--DAK-AKDI--DDVLILSGDHL--YRMDY--MDFVQSHRQ--RGA--DISICC
107  G---SVEVLAA--TQMPGEAAG-WFQG--TADAVR--KFI--WVLE--DYYKHKAI--EHILILSGDQL--YRMDY--MELVQKHVD--DNA--DITLSC
```

```
108  G---FVEVLAA--TQTTGESGKRWFQG--TADAVR--QFL--WLFE--DAR-LKRI--ENILILSGDHL--YRMDY--MDFVQKHVD--KGA--DISVAC
109  G---SVQVLAA--TQMPDEPAG-WFQG--TADAIR--KFM--WILE--DHYNQNNI--EHVVILCGDQL--YRMNY--MELVQKHVD--DNA--DITISC
110  G---SVEVLAA--TQMPGEAAG-WFQG--TADAVR--KFI--WVLE--DYYKHKAI--EHILILSGDQL--YRMDY--MELVQKHVD--DNA--DITLSC
111  G---SVQVLAD--TQMPEEPDG-WFQG--TADSVR--KFI--WVLE--DYYNHKSI--EHIVILSGDQL--YQMNY--MELVQKHVE--DNA--DITVSC
112  G---FVEVLAA--TQRPGSEGKRWFQG--TADAVR--QFD--WLFD--DAK-SKDI--DDVLILSGDHL--YRMDY--MDFVQSHRQ--RGA--GISICC
113  G---SVEVLAA--TQMPGEAAG-WFRG--TADAVR--KFI--WVLE--DYYKHKSI--EHILILSGDQL--YRMDY--MELVQKHVD--DNA--DITLSC
114  G---FVEVLAA--TQRPGSEGKRWFQG--TADAVR--QFA--WLFD--DAK-SKDI--EDVLILSGDHL--YRMDY--MDFVQSHRQ--RDA--GISICC
115  G---SVEVLAA--TQMPGEAAG-WFRG--TADAVR--KFI--WVLE--DYYKHKSI--EHILILSGDQL--YRMDY--MELVQKHVD--DNA--DITLSC
116  G---FVEVLAA--TQTPGEAGKKWFQG--TADAVR--QFH--WLFE--DAK-GKEI--EDVLILSGDHL--YRMDY--MDFVQSHRQ--SGA--DITISC
117  G---SVEVLAA--TQMPGEAAG-WFRG--TADAVR--KFI--WVLE--DYYKNKSI--EHILILSGDQL--YRMDY--MELVQKHVD--DNA--DITLSC
118  G---FVEVLAA--TQRPGSEGKTWFQG--TADAVR--QFA--WLFD--DAK-SKDI--EDVLILSGDHL--YRMDY--MDFVQSHRQ--RDA--GISICC
119  G---SVEVLAA--TQMPGEAAG-WFRG--TADAVR--KFI--WVLE--DYYKNKSI--EHILILSGDQL--YRMDY--MELVQKHVD--DNA--DITLSC
120  G---SVEVLAA--TQMPGEAAG-WFRG--TADAWR--KII--WVLE--DYYKNKSI--EHILILSGDQL--YRMDY--MELVQKHVD--DNA--DITLSC
121  G---FVEVLAA--QQTP-ESPS-WFEG--TADAVR--KYQ--WLFQ--EW----DV--DEYLILSGDQL--YRMDY--SLFVEHHRQ--SGA--DLTVAA
122  G---FAEVLAA--QQT-SVTNPQWFQG--TADAVR--QYL--WLME--EW----DV--EHFLILSGDHL--YRMDY--RDFVQRHID--TGA--DITLSV
123  G---FVEVLAA--QQT--PENPNWFQG--TADAVR--QYL--WLLN--EW----DA--DEYLILSGDHL--YRMDY--RQFIQRHRE--TGA--DITLSV
124  G---FVEVLAA--QQT--TENPSWFQG--TADAVR--QYG--WLFD--EW----DV--DEYLILSGDHL--YRMDY--SDFVKRHRE--TGA--DITLSV
125  G---FVEILAA--QQT--PENMNWFQG--TADAVR--QYL--WLFD--RA----EA--DEYLILSGDHL--YRMDY--RDFIQHHRE--TNA--DISLSV
126  G---FVEVLAA--QQT--PESPSWFEG--TADAVR--KYQ--WLFQ--EW----DV--DHYLILSGDQL--YRMDY--STFVDHHIA--TGA--DVSIGA
127  G---FVEVLAA--QQT--PESPSWFEG--TADAVR--KYQ--WLFQ--EW----DV--DHYLILSGDQL--YRMDY--SEFVNHHIA--TGA--DISIGA
128  G---FVEVLAA--QQT--PDSPTWFEG--TADAVR--KYQ--WLFQ--EW----DV--DQYLILSGDQL--YRMDY--SRFVDHHIQ--SGA--DLTVGA
129  G---FTEVLAA--QQT--ASNPNWFQG--TADAVR--QYI--WLFA--EW----DV--DYFLILSGDHL--YRMDY--REFVQRHID--TKA--DITLSV
130  G---FVEVLAA--QQT--PESPSWFEG--TADAVR--KYQ--WLFQ--EW----DV--DEYLILSGDQL--YRMDY--SLFVEHHRK--SGA--DLTVAA
131  G---FVEVLAA--QQT--PDSPTWFEG--TADAVR--KYQ--WLFQ--EW----DV--DEYLILSGDQL--YRMDY--SLFLEHHRR--TGA--KLTVAA
132  G---FVEVLAA--QQT--PESPSWFEG--TADAVR--KYQ--WLFQ--EW----DV--DEYLILSGDQL--YRMDY--SLFINHHRS--TGA--DLTVAA
133  G---FCEILAA--EQT--DENPNWFQG--TADAVR--QYL--WLLE--PS----GS--TEYLILSGDHL--YRMDY--SKFVRRHRE--TNA--DVTIAV
134  G---FVEVLAA--QQT--PDSPKWFEG--TADAVR--KYQ--WLFQ--EW----DV--DEYLILSGDQL--YRMDY--SLFVQHHRD--NKA--DLTVAA
135  G---FVEVLAA--QQT--PESPSWFEG--TADAVR--KYQ--WLFQ--EW----DV--DEYLILSGDQL--YRMDY--SLFVEHHRE--TGA--DLTVAA
136  G---FVEVLAA--QQT--PENPNWFQG--TADAVR--QYL--WLLA--DW----DV--DEYLILSGDHL--YRMDY--RLFVQRHRD--TGA--DVTLSV
137  G---FVEVLAA--QQT--AENPKWFQG--TADAVR--QYL--WAFQ--EW----DI--DEYLILSGDHL--YRMDY--RDFIQRHRE--TGA--DITLSV
138  G---FVEVLAA--QQT--PENPNWFQG--TADAVR--QYL--WLME--EW----DV--EEYLILSGDHL--YRMDY--RQFIQRHRD--TGA--DITLSV
139  G---FVEVLAA--QQT--PESPSWFEG--TADAVR--KYQ--WLFQ--EW----DV--DEYLILSGDQL--YRMDY--SLFVEHHRR--SGA--DLTVAA
140  G---FVDVLAA--QQT--PDNPDWFQG--TADAVR--QYL--WLMD--SW----KP--RDFLILSGDHL--YRMDY--RPFIHHHRQ--VGA--DVTLAV
141  G---FVDILAA--QQT--PDNPDWFQG--TADAVR--QYL--WLMD--SW----KP--RDFLILSGDHL--YRMDY--RPFIHYHRQ--TGA--DVTLAV
142  G---FVEVLAA--QQT--PESPSWFEG--TADAVR--KYQ--WLFQ--EW----DV--DEYLILSGDQL--YRMDY--SLFVEHHRR--SGA--DLTVAA
143  G---FVEVLAA--QQT--PDSPSWFEG--TADAVR--KYQ--WLFQ--EW----DV--DEYLILSGDQL--YRMDY--SLFVEHHRS--TGA--DLTVAA
144  G---FVEVLAA--QQT--PENPNWFQG--TADAVR--QYL--WMLQ--EW----DV--DEFLILSGDHL--YRMDY--RLFIQRHRE--TNA--DITLSV
145  G---FVEVLAA--QQT--PESPSWFEG--TADAVR--KYQ--WIFQ--EW----DV--DEYLILSGDQL--YRMDY--SQFVNHHRT--TGA--DLTVAA
146  G---FVEVLAA--QQT--PDSPKWFEG--TADAVR--KYQ--WLFQ--EW----DV--DEYLILSGDQL--YRMDY--SLFVQHHRD--NGA--DLTVAA
147  G---FVEVLAA--QQT--PETPSWFEG--TADAVR--KYQ--WLFQ--EW----DV--DEYLILSGDQL--YRMDY--SLFVEQHRK--TGA--DLTVAA
148  G---FVEVLAA--QQT--PDSPKWFEG--TADAVR--KYQ--WLFQ--EW----DV--DEYLILSGDQL--YRMDY--SLFVQHHRD--NGS--DLTVAA
149  G---FVEVLAA--QQT--PDSPKWFEG--TADAVR--KYQ--WLFQ--EW----DV--DEYLILSGDQL--YRMDY--SLFVQHHRD--NGA--DLTVAA
150  G---FVEVLAA--QQT--LDSPSWFEG--TADAVR--QYQ--TLFR--EW----DV--DEYLILSGDQL--YRMDY--SRFVEHHRS--TGA--DLTVAA
151  E---FVEVLAA--QQT--AENPSWFQG--TADAVR--QYL--WLME--EW----DV--DEYLILSGDHL--YRMDY--REYIQRHRE--TKA--DITLSV
152  G---FVEVLAA--QQT--PDSPKWFEG--TADAVR--KYQ--WLFQ--EW----DV--DEYLILSGDQL--YRMDY--SLFVQHHRD--NGA--DLTVAA
153  G---FVEVLAA--QQT--KDNPDWFQG--TADAVR--QYL--WLFR--EW----DV--DEYLILSGDHL--YRMDY--AQFVKRHRE--TNA--DITLSV
154  G---FVEVLAA--QQT--AENPKWFQG--TADAVR--QYL--WAFQ--EW----DI--DEYLILSGDHL--YRMDY--RDFIQRHRE--TGA--DITLSV
155  G---FVEVLAA--QIT--PENPNWFQG--TADAVR--QYL--WLIK--EW----DV--DEYLILSGDHL--YRMDY--SQFIQRHRD--TNA--DITLSV
156  G---FVEVLAA--QQT--PENPDWFQG--TADAVR--QYL--WLLS--DW----EV--DYYLILSGDHL--YRMDY--RLFVNRHRD--TNA--DITLSV
157  G---FVEVLAA--QIT--PENPNWFQG--TADAVR--QYL--WLIK--EW----DV--DEYLILSGDHL--YRMDY--SQFIQRHRD--TNA--DITLSV
158  G---FVEVLAA--QQT--PDSPTWFEG--TADAVR--KYQ--WLFQ--EW----DV--DEYLILSGDQL--YRMDY--SLFIGHHRR--SGA--DLTVAA
159  G---FVEVLAA--QQT--PDSPSWFEG--TADAVR--KYQ--WLFQ--EW----DV--DEYLILSGDQL--YRMDY--SLFLEHHRR--TGA--NLTVAA
160  G---FVEVLAA--QQT--PDNSGWFEG--TADAVR--QYL--QLLK--EW----DV--DEYLILSGDHL--YRMDY--SRFVQRHRD--TNA--DITISV
161  G---FAEVLAA--QQT--PENPNWFQG--TADAVR--QYM--WMFA--EQR---DV--DEILILSGDHL--YRMDY--SVFIERHRS--TNA--DITLSV
162  G---FVEVLAA--QQT--KDNPEWFQG--TADAVR--KYI--WLFK--EW----DI--DYYLILSGDHL--YRMDY--RDFVQRHID--TKA--DITLSV
163  G---FVEVLAA--QQT--PENPNWFQG--TADAVR--QYL--WMLQ--EW----DV--DEFLILSGDHL--YRMDY--RLFIQRHRE--TNA--DITLSV
164  G---FVEILAA--QQT--PENMNWFQG--TADAVR--QYL--WLFD--RA----EA--DEYLILSGDHL--YRMDY--RDFIQHHRK--TNA--DITLSV
165  G---FVEVLAA--QQT--TENPSWFQG--TADAVR--QYG--WLFD--EW----DV--DEYLILSGDHL--YRMDY--SDFVKRHRE--TGA--DITLSV
166  G---FTEVLAA--QQT--KENPDWFQG--TADAVR--QYS--WLLE--DW----DV--DEYIILSGDHL--YRMDY--REFIQRHRD--TGA--DITLSV
167  G---FVEVLAA--QQT--PDSPKWFEG--TADAVR--KYQ--WLFQ--EW----DV--DEYLILSGDQL--YRMDY--SLFVQHHRD--NEA--DLTVAA
168  G---FVEVLAA--QQT--PETPSWFEG--TADAVR--KYQ--WLFQ--EW----DV--DEYLILSGDQL--YRMDY--SLFVEQHRN--TGA--DLTVAA
169  G---FVEVLAA--QQT--PDSPSWFEG--TADAVR--KYE--WLLQ--EW----DI--DEVLILSGDQL--YRMDY--AHFVAQHRA--SGA--DLTVAA
170  G---FVEVLAA--QQT--PESPSWFEG--TADAVR--KYQ--WLFQ--EW----DV--DEYLILSGDQL--YRMDY--SLFINHHRS--TGA--DLTVAA
171  G---FVEVLAA--QQT--MENPQWFQG--TADAVR--QYI--WTMK--DW----DI--DEYLILSGDHL--YRMDY--SKFIERHRE--TNA--DITLSV
172  G---FVEVLAA--QQT--PDSPKWFEG--TADAVR--KYQ--WLFQ--EW----DV--DEYLILSGDQL--YRMDY--SLFVQHHRD--NEA--DLTVAA
173  G---FVEVLAA--QQT--PDSPSWFEG--TADAVR--KYQ--WLFQ--EW----DV--DHYLILSGDQL--YRMDY--SRFVQHHID--TGA--DLSVGA
174  E---FVEVLAA--QQT--PENPGWFQG--TADAVR--QYL--WLME--EW----DI--DEYLILSGDHL--YRMDY--RQFIQRHRE--TNA--DITLSV
175  G---FVEVLAA--QQT--PENPNWFQG--TADAVR--QYI--WMLQ--DW----DV--DEFLILSGDHL--YRMDY--RLFIQRHRE--TNA--DITLSV
176  G---FVEILPA--QKT--AENPSWFQG--TADAVR--QYL--WLFN--GW----DV--DEYLILSGDHL--YRMDY--RLFVQRHRD--TGA--DITLSV
177  G---FVEVLAA--QQT--PENLSWFQG--TADAVR--QYL--WLFE--EW----DV--DEYLILSGDHL--YRMDY--RQFIQRHRE--TGA--DITLSV
178  G---FVEVLAA--QQT--TENPSWFQG--TADAVR--QYG--WLFD--EW----DV--DEYLILSGDHL--YRMDY--SDFVKRHRE--TGA--DITLSV
179  G---FVEILAA--QQT--PENMNWFQG--TADAVR--QYL--WLFD--RA----EA--DEYLILSGDHL--YRMDY--RDFIQHHRK--TNA--DITLSV
180  G---FVEVLAA--QQT--LDSPSWFEG--TADAVR--QYQ--TLFS--EW----DV--DEYLILSGDQL--YRMDY--SRFVEHHRS--TGA--DLTVAA
181  G---FVEVLAA--QQT--KENPSWFQG--TADAVR--QYL--WLFN--EW----DV--DEYLILSGDHL--YRMDY--DDFIKQHRI--TGA--DITLAV
182  G---FVEVLAA--QQT--QENPNWFQG--TADAVR--QYL--SLLE--QW----DV--DEYLILSGDHL--YRMDY--QKFVQRHRE--TNA--DITLSV
183  G---FVEVLAA--QQT--KENPNWFQG--TADAVR--QYL--WLFE--EW----DI--DHYLILSGDHL--YRMDY--REFVQRHLD--TKA--DITLSV
184  G---FVEVLAA--QQT--PENPNWFQG--TADAVR--QYI--WMLQ--EW----DV--DEFLILSGDHL--YRMDY--RLFIQRHRE--TNA--DITLSV
```

```
185  G---FVEVLAA--QQT--PENPNWFQG--TADAVR--QYI--WMLE--EW----DV--DEFLILSGDHL--YRMDY--RLFVQRHRE--TNA--DITLSV
186  G---FAEVLAA--QQT-SVTNPQWFQG--TADAVR--QYL--WLME--EW----DV--EHFLILSGDHL--YRMDY--RDFVQRHID--TGA--DITLSV
187  G---FVEVLAA--QQT--AENPSWFQG--TADAVR--QYL--WLFD--EW----DV--DQYLILSGDHL--YRMDY--SDFVRRHQE--TGA--DITLSV
188  G---FVEVLAA--QQT--PDSPTWFEG--TADAVR--KYQ--WLFQ--EW----DV--DEYLILSGDQL--YRMDY--SLFIEHHRR--SGA--DLTVAA
189  SG--FVEVLAA--TQTPGLGGKEWFQG--TADAVR--QYS--WLFE--DVK-NKDV--QDVVILSGDHL--YRMDY--MAFVDRHRE--VNA--DITIGC
190  G---FVEVLAA--QQS--PKSKVWFQG--TADAVR--QYM--WLFN--ES----KC--EEYIILSGDHL--YRMDY--KPFILEHRK--TGA--DITVSA
191  NG--FVEVLAA--TQTPGLGGKEWFQG--TADAVR--QYS--WLFE--DIK-NKDV--QDIVILSGDHL--YRMDY--MAFVARHRE--VNA--DITIGC
192  G---FVEVLAA--QQS--PKNKDWFQG--TADAVR--QYI--WLFN--ES----KC--DEYIILSGDHL--YRMDY--KPFILKHRQ--TKA--DITVSA
193  DG--FVEVLAA--TQTP-TD-KEWFQG--TADAVR--QYS--WLLE--DTK-NRAI--EDVLILSGDHL--YRMDY--MKFVNYHRE--TNA--DITIGC
194  G---FVEVLAA--SQS--SANKSWFQG--TADAVR--QYM--WLFE--EAV-REGV--EDFLILSGDHL--YRMDY--RDFVRKHRN--SGA--AITIAA
195  NG--FVEVLAA--TQTPGQGGKEWFQG--TADAVR--QYS--WLFN--DVK-NKDV--EDIVILAGDHL--YRMDY--MKFVEAHRE--SNA--DISVGT
196  G---FVEVLAA--QQS--PVNKAWFQG--TADAVR--QYL--WLFE--ES----KC--EEYLILSGDHL--YRMDY--RPFIMKHRE--TEA--AITVAA
197  G---FVEVLAA--SQS--SANKSWFQG--TADAVR--QYM--WLFE--EAV-REGV--EDFLILSGDHL--YRMDY--RDFVRKHRE--SGA--AITIAA
198  DG--FVEVLAA--TQTP-TD-KEWFQG--TADAVR--QYS--WLLE--DTK-NRAI--EDVLILSGDHL--YRMDY--MKFVNYHRE--TNA--DITIGC
199  NG--FVEVLAA--TQTPGQGGKEWFQG--TADAVR--QYS--WLFN--DVK-NKDV--EDIVILAGDHL--YRMDY--MKFVEAHRE--SNA--DITVGT
200  G---FVEVLAA--QQS--PINKAWFQG--TADAVR--QYL--WLFA--ES----GC--EEYLILSGDHL--YRMDY--RPFIRDHRA--KNA--DITVAA
201  G---FVEVLAA--TQTPGEAGMNWFQG--TADAVR--QFT--WVFE--DTR-SKEI--ENVLVLSGDHL--YRMDY--MEFIQKHQD--TGA--DITIGC
203  G---FVEVLAA--QQS--PENPNWFQG--TADAVR--QYM--WLFE--EQ----PV--MEYLILAGDHL--YRMDY--QKFIQAHRI--TDA--DITVAA
204  G---FVEVLAA--QQS--PENPNWFQG--TADAVR--QYM--WLFE--EQ----PV--MEYLILAGDHL--YRMDY--QKFIQAHRI--TDA--DITVAA
206  G---FVEVLAA--TQTPGESGMNWFQG--TADAVR--QFT--WLFE--DVR-NKDV--DYVLVLSGDHL--YRMDY--MDFVQKHKD--SGA--DITISC
207  G---FVEVLAA--TQTPGESGMNWFQG--TADAVR--QFT--WLFE--DVR-NKDV--DYVLVLSGDHL--YRMDY--MDFVQKHKD--SGA--DITISC
208  G---FVEVLAA--TQTPGEAGMNWFQG--TADAVR--QFT--WVFE--DTR-SKEI--ENVLVLSGDHL--YRMDY--MEFIQKHQD--TGA--DITIGC
209  G---FVEVLAA--TQTPGEAGMNWFQG--TADAVR--QFT--WVFE--DTR-SKEI--ENVLVLSGDHL--YRMDY--MEFIQKHQD--TGA--DITIGC

               210       220       230       240       250       260       270       280       290       300
     ....|....|....|....|....|....|....|....|....|....|....|....|....|....|....|....|....|....|....|....|
1    LPM--D--EKRAT--A--FGLMKID--EE---GRIIEFAEK--PQG---EQLQA--MKVDTTILGLDDKR-AKEMP--FIASM--G---IYVISK--DVM
2    LPM--D--EKRAT--A--FGLMKID--EE---GRIIEFAEK--PQG---EQLQA--MKVDTTILGLDDKR-AKEMP--LVASM--G---IYVISK--DVM
3    LPM--D--EKRAT--A--FGLMKID--EE---GRIIEFAEK--PQG---EQLQA--MKVDTTILGLDDKR-AKEMP--FIASM--G---IYVISK--DVM
4    LPM--D--EKRAT--A--FGLMKID--EE---GRIIEFAEK--PQG---EQLQA--MKVDTTILGLDDKR-AKEMP--FIASM--G---IYVISK--DVM
5    LPM--D--EQRAT--A--FGLMKID--EE---GRIIEFAEK--PKG---EHLKA--MKVDTTILGLDDQR-AKEMP--FIASM--G---IYVVSR--DVM
6    LPM--D--EQRAT--A--FGLMKID--EE---GRIVEFAEK--PKG---EHLKA--MKVDTTILGLDDKR-AKEMP--YIASM--G---IYVVSR--DVM
7    LPM--D--EKRAT--A--FGLMKID--EE---GRIIEFSEK--PKG---DQLQA--MKVDTTILGLDDER-AKEMP--FIASM--G---IYVISK--NVM
8    LPM--D--EARAT--A--FGLMKID--EE---GRIIEFAEK--PKG---EQLKA--MKVDTTILGLDDER-AKEMP--YIASM--G---IYVVSK--HVM
9    LPM--D--EARAT--A--FGLMKID--EE---GRIIEFAEK--PKG---EQLKA--MKVDTTILGLDDER-AKEMP--YIASM--G---IYVVSK--NVM
10   LPM--D--EKRAT--A--FGLMKID--EE---GRIIEFAEK--PKG---EQLKA--MKVDTTILGLDDER-AKELP--YIASM--G---IYVVSK--NVM
11   LPM--D--EKRAT--A--FGLMKID--EE---GRIIEFAEK--PKG---EQLKA--MKVDTTILGLDDER-AKEMP--YIASM--G---IYVVSK--NVM
12   LPM--D--EKRAT--A--FGLMKID--EE---GRIIEFAEK--PKG---EQLKA--MKVDTTILGLDDER-AKEMP--YIASM--G---IYVVSK--NVM
13   LPM--D--EKRAT--A--FGLMKID--EE---GRIIEFAEK--PKG---EQLKA--MKVDTTILGLDDER-AKEMP--YIASM--G---IYVVSK--DVM
14   LPM--D--EKRST--A--FGLMKID--DE---GRIIEFAEK--PKG---EQLKA--MKVDTTILGLDDER-AKEIP--FIASM--G---IYVVSK--NVM
15   LPM--D--EKRAT--A--FGLMKID--EE---GRIIEFAEK--PKG---EQLKA--MKVDTTILGLDDER-AKEMP--FIASM--G---IYVISK--DVM
16   LPM--D--EARAT--A--FGLMKID--EE---GRIVEFSEK--PKG---EQLKA--MKVDTTILGLDDER-AKEMP--YIASM--G---IYVVSK--HVM
17   LPM--D--EKRAT--A--FGLMKID--EE---GRIIEFAEK--PKG---EQLKA--MKVDTTILGLDDER-AKEMP--FIASM--G---IYVISK--NVM
18   LPM--D--ELRAT--A--FGLMKID--EE---GRIIEFAEK--PKG---EQLKA--MKVDTTILGLDEER-AKEMP--YIASM--G---IYVVSK--HVM
19   LPM--D--EKRAT--A--FGLMKID--EE---GRIIEFAEK--PKG---DQLNA--MKVDTTILGLDDER-AKEMP--FIASM--G---IYVISK--NVM
20   LPM--D--EKRAT--A--FGLMKID--EE---GRIIEFAEK--PKG---EQLKT--MKVDTTILGLDDAR-GKEMP--FIASM--G---IYVISK--DVM
21   LPM--D--EKRAT--A--FGLMKID--EE---GRIIEFAEK--PKG---EQLKA--MKVDTTILGLDDER-AKEMP--YIASM--G---IYVISK--DVM
22   LPM--D--EKLAT--A--FGLMKID--EE---GRIIEFSEK--PKG---EQLKA--MKVDTTILGLDDAR-GKEMP--FIASM--G---IYVISK--DVM
23   LPM--D--EKRAT--A--FGLMKID--EE---GRIIEFSEK--PKG---EQLKA--MKVDTTILGLDDER-AKEMP--YIASM--G---IYVISK--DVM
24   LPM--D--EKRAT--A--FGLMKID--DE---GRIIEFAEK--PKG---EQLKA--MKVDTTILGLDDER-AKEMP--YIASM--G---IYVVSK--NVM
25   LPM--D--EKRAT--A--FGLMKID--EE---GRIIEFAEK--PKG---EQLKA--MKVDTTLFGLDDQR-AKEMP--YIASM--G---IYVVSK--DVM
26   LPM--D--EKRAT--A--FGLMKID--EE---GRIIEFAEK--PKG---EQLKA--MKVDTTIFGLDDQR-AKELP--FIASM--G---IYVISK--NVM
27   LPM--D--EKRAT--A--FGLMKID--DE---GRIIEFSEK--PKG---EALKA--MRVDTTILGLDDER-AKEMP--YIASM--G---IYVFSK--NAM
28   LPM--D--EKRPT--A--FGLMKID--EE---GRIIEFAEK--PKG---EQLKA--MKVDTTILGLDDKR-AKEMP--YIASM--G---IYVVSK--DVM
29   LPM--D--ENRAT--A--FGLMKID--EE---GRIIEFAEK--PKG---EQLKA--MKVDTTIFGLDDER-AKEMP--YIASM--G---IYVVSK--NVM
30   LPM--D--EARAT--A--FGLMKID--EE---GRIIEFSEN--PKG---EQLKA--MKVDTTILGLDDDR-AKEMP--YIASM--G---IYVVSK--HVM
31   LPM--D--EKRAT--A--FGLMKID--EE---GRIIEFAEK--PKG---EQLKA--MKVDTTILGLDDER-AKEMP--FIASM--G---IYVISK--NVM
32   LPM--D--EQRAT--A--FGLMKID--DE---GRIVEFAEK--PKG---EKLRS--MMVDTTILGLDPER-AKELP--YIASM--G---IYVFSK--DVM
33   LPM--D--EKRAT--A--FGLMKID--EE---GRIIEFAEK--PKG---EQLKA--MMVDTTILGLDDVR-AKEMP--YIASM--G---IYVFSK--DVM
34   LPM--D--EKRAT--A--FGLMKID--EE---GRIIEFAEK--PKG---EQLKA--MMVDTTILGLDDVR-AKEMP--YIASM--G---IYVFSK--DVM
35   LPM--D--EKRAT--A--FGLMKID--EE---GRIIEFAEK--PKG---EQLKA--MMVDTTILGLDDVR-AKEMP--YIASM--G---IYVFSK--DVM
36   LPM--D--EKRAT--A--FGLMKID--EE---GRIIEFAEK--PKG---DQLKA--MMVDTTILGLDDER-AKEMP--YIASM--G---IYVFSK--DVM
37   LPM--D--EKRAT--A--FGLMKID--EE---GRIVEFAEK--PKG---EQLKA--MMVDTTILGLDDVR-AKEMP--YIASM--G---IYVISK--NVM
38   LPM--D--EERAT--A--FGLMKID--DE---GRIIEFAEK--PKG---EKLKS--MMVDTTILGLDTER-AKELP--YIASM--G---IYVFSK--DVM
39   LPM--D--EKRAT--A--FGLMKID--EE---GRIVEFAEK--PKG---EQLKA--MMVDTTILGLDDVR-AKEMP--YIASM--G---IYVISK--NVM
41   LPM--D--EERAT--A--FGLMKID--EE---GRIIEFAEK--PKG---EQLKA--MMVDTTILGLDDVR-AKEMP--YIASM--G---IYVISK--HVM
42   LPM--D--EERAT--A--FGLMKID--EE---GRIIEFAEK--PKG---EQLKA--MMVDTTILGLDDVR-AKEMP--YIASM--G---IYVISK--HVM
43   LPM--D--EQRAT--A--FGLMKID--DE---GRIVEFAEK--PKG---EKLRS--MMVDTTILGLDPER-AKELP--YIASM--G---IYVFSK--DVM
44   LPM--D--EERAT--A--FGLMKID--DE---GRIVEFSEK--PKG---EKLKA--MMVVTTILGLDSER-AKELP--YIASM--G---IYVFSK--DAM
45   LPM--D--EERAT--A--FGLMKID--EE---GRIIEFAEK--PKG---EQLKA--MMVDTTILGLEDAR-AKEMP--YIASM--G---IYVISK--HVM
46   LPM--D--EERAT--A--FGLMKID--EE---GRIIEFAEK--PKG---EQLKA--MMVDTTILGLEDAR-AKEMP--YIASM--G---IYVISK--HVM
47   LPM--D--EERAT--A--SGLMKID--DE---GRIVEFSEK--PKG---EKLKA--MMVDTTILGLDSER-AKELP--YIASM--G---IYVFSK--DAM
48   LPM--D--EERAT--A--FGLMKID--EE---GRIIEFAEK--PKG---EQLKA--MMVDTTILGLDDAR-AKEMP--YIASM--G---IYVISK--HVM
49   APA--E--DSRAS--D--FGLVKID--SR---GRVVQFAEK--PKG---FDLKA--MQVDTTLVGLSPQD-AKKSP--YIASM--G---VYVFKT--DVL
50   VPM--D--DGRAS--D--FGLMKID--ET---GAIIQFAEK--PKG---PALKA--MQVDTSILGLSEQE-ASNFP--YIASM--G---VYVFKT--DVL
51   LPI--D--DSRAS--D--FGLMKID--DT---GRVMSFSEK--PKG---DDLKA--MAVDTTVLGLSPEE-AKEKP--YIASI--G--KVYVFKK--DIL
52   IPI--D--DRRAS--D--FGLMKID--DK---GRVISFSEK--PKG---DDLKA--MAVDTTILGLSKEE-AEKKP--YIASM--G---VYVFKK--EIL
```

```
53   LPM--D--ESRAS--D--FGLLKID--QS---GKIIQFSEK--PKG---DDLKA--MQVDTSILGLPPKE-AAESP--YIASM--G---VYVFRK--EVL
54   APV--D--ESRAS--E--YGLVNID--RS---GRVVHFSEK--PTG---IDLKS--MQTDTTMHGLSHQE-AAKSP--YIASM--G---VYCFKT--EAL
55   APV--S--ESRAS--N--FGLVKID--RG---GRVIHFSEK--PTG---VDLKS--MQTDTTMLGLSHQE-ATDSP--YIASM--G---VYCFKT--EAL
56   APA--E--DSRAS--D--FGLVKID--SR---GRVVQFAEK--PKG---FDLKA--MQVDTTLVGLSPQD-AKKSP--YIASM--G---VYVFKT--DVL
57   VPM--D--DGRAS--D--FGLMKID--ET---GRIIQFAEK--PKG---PALKV--MQVDTSILGLSEQE-ASNFP--YIASM--G---VYVFKT--DVL
58   LPI--D--DSRAS--D--FGLMKID--DT---GRVMSFSEK--PKG---DDLKA--MAVDTTVLGLSPEE-AKEKP--YIASM--G---VYVFKK--DIL
59   APA--E--DSRAS--D--FGLVKID--SR---GRVVQFAEN--QR----FELKA--MLVDTSLVGLSPQD-AKKSP--YIASM--G---VYVFKT--DVL
60   VPM--D--DGRAS--D--FGLMKID--ET---GRIIQFVEK--PKG---PALKA--MQVDTSILGLSEQE-ASNFP--YIASM--G---VYVFKT--DVL
61   IPI--D--DRRAS--D--FGLMKID--DK---GRVISFSEK--PRG---DELKA--MAVDTTILGLSKEE-AEKKP--YIASM--G---VYVFKK--EIL
62   LPM--D--ESRAS--D--FGLLKID--QS---GKIIQFSEK--PKG---DDLKA--MQVDTSILGLPPKE-AAESP--YIASM--G---VYVFRK--EVL
63   APV--D--ESRAS--D--YGLVNID--RS---GRVVHFSEK--PTG---IDLKS--MQTDTTMHGLSHQE-AAKSP--YIASM--G---VYCFKT--EAL
64   APV--S--ESRAS--N--FGLVKID--RG---GRVIHFSEK--PTG---VDLKS--MQTDTTMLGLSHQE-ATDSP--YIASM--G---VYCFKT--EAL
66   LPV--D--GSRAS--D--FGLVKVD--ER---GQICQFLEK--PKG---ELLRS--MHVDTSIFGLSAQE-ARKFP--YIASM--G---IYVFKI--DVL
67   LPM--D--DSRAS--D--FGLMRID--NK---GRILSFSEK--PKG---EELKA--MQVDTTVLGLSKDE-AQKKP--YIASM--G---VYVFKK--EIL
68   VPM--D--ESRAS--D--YELMKID--RK---GQITQFVEK--PEG---SDLQA--MHVDTTLLGLTAEE-AQTYP--YIAPM--G---VSVFRT--ETL
69   VPM--D--ESRAS--D--YELMKID--RK---GEITQFVEK--PEG---SDLKA--MHVDTTLLGLTAEE-AQTYP--YIAPM--G---VSVFRT--ETL
70   AAV--G--ESRAS--D--YGLVKAD--GR---GRIIQFSEK--PKG---ADLKA--MQVDTSVLGLPPHE-AKRSP--YIASM--G---VYVFKT--DVL
71   AAV--G--ESRAS--D--YGLVKAD--AR---GRIIQFSEK--PNG---ADLKA--MQVDTSVLGLPLHE-AKRSP--YIASM--G---VYVFKT--DVL
72   LPM--D--DSRAS--D--FGLMKID--NK---GRVLSFSEK--PKG---EELKA--MQVDTTVLGLSKDE-AQKKP--YIASM--G---VYVFKK--EIL
73   VPM--D--DSRAS--D--YGLMKID--KT---GRIIQFAEK--PKG---SDLKA--MRVDTTLLGLLPQE-AEKHP--YIASM--G---VYVFRT--ETL
74   AAV--G--DSRAS--D--YGLVKVD--DR---GRIIQFSEK--PKG---DDLKA--MQADTSLLGLSSQD-ALESP--YIASM--G---VYVFKT--DVL
75   VPM--D--DSRAS--D--YGLMKID--KT---GRIIQFAEK--PKG---SDLKA--MRVDTTLLGLSPQE-AEKYP--YIASM--G---VYVFRT--ETL
76   AAV--G--DSRAS--D--YGLVKVD--DR---GRIIQFSEK--PNG---DDLKA--MQADTSLLGLSPQD-ALKSP--YIASM--G---VYVFKT--DVL
77   LPV--D--GSRAS--D--FGLVKVD--ER---GQIRQFLEK--PKG---ELLRS--MHVDTSIFGLSAQE-ARKFP--YIASM--G---IYVFKI--DVL
80   AAV--G--DSRAS--D--YGLVKVD--SG---GRIIQFSEK--PKG---ADLKS--MQADTSLFGLSNQD-ALRSP--YIASM--G---VYVFKT--DVL
81   LPV--N--DSRAS--D--FGLVKID--ET---GQIRQFLEK--PKG---ENLKS--MKVDTTVLGLSAQE-ANKFP--YIASM--G---IYMFKT--DVL
82   AAV--G--ESRAS--D--YGLVKID--GR---GQVFQFAEK--PKG---SELRE--MRVDTTRLGLSPQD-AMKSP--YIASM--G---VYVFKT--DIL
84   LPM--D--DSRAS--D--FGLMKID--NK---GRVLSFSEK--PKG---VDLKA--MEVDTTVLGLSKEE-ALKKP--YIASM--G---VYVFKK--EIL
87   LPM--D--DSRAS--D--FGLMNID--NK---GRVLSFSEK--PKG---ADLKA--MAVDTTVLGLSKEE-AEKKP--YIASM--G---VYVFKK--EIL
88   LPV--D--ESRAS--D--FGLIKID--ET---GQIRQFLEK--PKG---ESLKS--MRVDTSTLGLSISD-ARKLP--YIASM--G---IYMFKT--DVL
89   AAV--G--ESRAS--D--YGLVKID--SR---GRIVHFAEK--PGG---AELKS--LKADTTQLGLSPQD-ALKSP--YIASM--G---VYVFRT--EIL
90   VPM--D--DSRAS--D--YGLMKID--NT---GRIIQFAEK--PKG---LDLKA--MQIDTKLLGLSKQD-ALQYP--YIASM--G---VYVFRT--EVL
91   VPM--D--DSRAS--D--YGLMKID--NT---GRIIQFSEK--PKG---PNLKA--MKVNTTLLGLSEKE-AEKCP--YIASM--G---VYVFRT--DVL
92   LPM--D--ESRAS--D--FGLIKID--EM---GQIRQFLEK--PKG---ETLKS--MRVDTTALGLSPVE-AKKFP--YIASM--G---IYLFKT--DVL
94   LPM--D--DSRAS--D--FGLMKID--NK---GRVLFFSEK--PKG---EDLKA--MEVDTKVLGLSREE-AEKKP--YIASM--G---VYVFKK--EIL
95   VPV--G--ESRAS--D--YGLLKMD--NR---GRIIQFAEK--PKG---ADLKA--MKVDTTRLGLSPQE-AMKSP--YIASM--G---VYVFKT--DIL
96   VPM--D--DSRAS--D--YGLMKID--NT---GRIVQFAEK--PKG---PDLKA--MQVDTTLLGLSRQE-AMQFP--YIASM--G---VYVFRT--DVL
97   VAV--G--ESRAS--D--YGLVKID--SK---GQIFQFTEK--PKG---SELRE--MQVDTTHLGLSPQD-ALKSS--YIASM--G---VYVFKT--DIL
98   VPM--D--DSRAS--D--YGLMKID--ST---GRIIQFAEK--PKG---TDLKA--MQVDTTLLGLSKQE-AMQFP--YIASM--G---VYVFRT--DVL
99   LPI--D--GSRAS--D--FGLMKID--DT---GRVISFSEK--PKG---DELKA--MQVDTTVLGLSKEE-AENKP--YIASM--G---IYIFKK--DIL
100  APV--D--ESRAS--K--NGLVKID--HT---GRVLQFFEK--PKG---ADLNS--MRVETNFLSYAIDD-AQKYP--YLASM--G---IYVFKK--DAL
101  APV--G--ESRAS--D--YGLVKFD--SS---GRVIQFSEK--PKG---AALEE--MKVDTSFLNFATCTLPAEYP--YIASM--G---VYVFKR--DVL
102  VPM--D--ESRAS--D--FGLMKAD--RN---GHITDFLEK--PKG---ADLES--MQVDMGLFGLSPEF-ASTYK--YMASM--G---IYVFKA--DVL
103  APV--G--ESRAS--E--YGLVKFD--SS---GRVIQFSEK--PKG---VDLEA--MKVDTSFLNFAIDD-PAKFP--YIASM--G---VYVFKR--DVL
104  VPM--D--ESRAS--D--FGLMKTD--RN---GRITDFLEK--PKG---ESLKS--MVVDMEIFGLSPEV-ANVYN--YLASM--G---IYVFKT--DVL
105  LPI--D--DSRAS--D--FGLMKID--DT---GRVISFSEK--PKG---DDLKA--MQVDTTVLGLSKEE-AEEKP--YIASM--G---VYIFKK--EIL
106  LPI--D--DSRAS--D--FGLMKID--DT---GRVIAFSEK--PKG---DDLKA--MQVDTTVLGLPQDE-AKEKP--YIASM--G---VYIFKK--EIL
107  APV--G--ESRAS--D--YGLVKFD--SS---GRVIQFSEK--PKG---TDLEA--MKVDTSFLNFAIDD-PTKFP--YIASM--G---VYVFKR--DVL
108  VPV--D--ESRAS--D--FGLMKTD--KN---GRITDFLEK--PKD---ESLKS--MQLDMGTFGLRPEV-ADTCK--YMASM--G---IYVFRT--DIL
109  API--D--GSRAS--D--YGLVKFD--DS---GRVIQFLEK--PEG---ADLES--MKVDTSFLSYAIDD-KQKYP--YIASM--G---IYVLKK--DVL
110  APV--G--ESRAS--D--YGLVKFD--SS---GRVIQFSEK--PKG---AALEE--MKVDTSFLNFAIDD-PTKYP--YIASM--G---VYVFKR--DVL
111  APV--D--ESRAS--N--NGLVKCD--HT---GRVLQFFEK--PKG---ADLNS--MRVDTNFLSYAIGD-AQKYQ--YIASM--G---IYVFKK--DAL
112  LPI--D--DSRAS--D--FGLMKID--DT---ARVISFSEK--PKG---DELKA--MQVDTTVLGLSKEE-AEKKP--YIASM--G---VYIFKK--DIL
113  APV--G--ESRAS--E--YGLVKFD--SS---GRVIQFSEK--PKG---DDLEA--MKVDTSFLNFAIDD-PAKYP--YIASM--G---VYVFKR--DVL
114  LPI--D--DSRAS--D--FGLMKID--DT---GRVISFSEK--PKG---ADLKA--MQVDTTLLGLPKEE-AEKKP--YIASM--G---VYIFKK--EIL
115  APV--G--ESRAS--E--YGLVKFD--SS---GRVIQFSEK--PKG---DDLEA--MKVDTSFLNFAIDD-PAKYP--YIASM--G---VYVFKR--DVL
116  VPM--D--VSRAS--D--FGLMKID--NN---GRVLSFSEK--PKG---QELKA--MEVDTSVLGLSREQ-AKKTP--FIASM--G---VYVFKK--EIL
117  APV--G--ESRAS--E--YGLVKFD--SS---GRVVQFSEK--PKG---DDLEA--MKVDTSFLNFAIDD-PAKYP--YIASM--G---VYVFKR--DVL
118  LPI--D--GSRAS--D--FGLMKID--DT---GRVISFSEK--PRG---ADLKA--MQVDTTLLGLPKEE-AEKKP--YIASM--G---VYIFKK--EIL
119  APV--G--ESRAS--E--YGLVKFD--SS---GRVVQFSEK--PKG---DDLEA--MKVDTSFLNFAIDD-PAKYP--YIASM--G---VYVFKR--DVL
120  APV--G--ESRAS--E--YGLVKFD--SS---GRVIQFSEQ--PKG---DDLEA--MKVDTSFLNFAIDD-PAKYP--YIASM--G---VYVFKR--DVL
121  LPV--D--PQQAE--A--FGLMRTD--AH---GTIQEFREK--PKG---DSLKE--MAVDTSRFGLSPES-AEQKP--YLASM--G---IYVFSR--KAL
122  LPV--D--EKRAS--S--FGLMKID--EST--GRIIDFSEK--PKG---EALKQ--MAVDTSTLGLSPEE-AAESP--YIASM--G---IYVFKK--DVL
123  IPI--D--KSRAS--D--FGLMKID--QS---GRVIDFSEK--PKG---DELDR--MQVDTSVLGLSPEQ-AKLQP--YIASM--G---IYVFKK--DVL
124  VPI--D--EKRAS--S--FGLMKIN--DN---GRIVDFAEK--PKG---EELKQ--MQVDTSILGLNPEQ-AKESP--YIASM--G---IYVFNK--KAL
125  LPV--D--EKQAS--S--FGLMKID--DT---GRIIDFQEK--PKG---DDLKR--MQVDTTTLGLSAEE-SKIKP--YIASM--G---IYLFKR--EVL
126  LPV--D--AAQAE--G--FGLMHTN--EH---GRIREFREK--PKG---EALKE--MWVDTSKLGLSADE-ALKRP--YLASM--G---IYVFSR--ETL
127  LPV--D--APQAE--A--FGLMHTD--EK---GKIREFREK--PKG---DALKE--MWVDTSRLGLSPEE-AEKRP--YLASM--G---IYVFSR--ETL
128  LPV--D--AEQAE--G--FGLMRTD--LD---GRIREFSEK--PKG---AALEA--MKVDTARLGLAEAE-ATRRP--YLASM--G---IYVFSR--DTL
129  LPI--D--EKRAS--D--FGLMKIN--DT---GRIIDFSEK--PKG---DALKK--MAVDTTTLGLSAEE-AKESP--YIASM--G---IYVFNR--EVL
130  LPV--D--PQQAE--A--FGLMRTD--EH---GTIQEFREK--PKG---DSLKE--MAVDTSRFGLSPES-AQSKP--YLASM--G---IYVFSR--KAL
131  LPV--D--AKQAE--S--FGLMRTD--SE---GNIQEFREK--PKG---DSLLE--MAVDTSRFGLSPES-AQERP--YLASM--G---IYVFSR--ETL
132  LPV--D--AKQAE--A--FGLMRTD--QD---GRILEFREK--PKG---DSLLE--MAVDTSRFGLSADS-AKERP--YLASM--G---IYVFSR--DTL
133  LPC--D--LERAS--D--FGLIKTD--AD---GRVVQFTEK--PKG---AELER--MRVDTTTLGLTLEE-AERRP--FVASM--G---IYVFRH--DVM
134  LPV--D--ESQAE--G--FGLMRTD--DL---GNIKEFSEK--PTG---EKLKS--MAVDTSKFGLTKES-ASEKP--YLASM--G---IYVFSR--KTL
135  LPV--D--GAQAE--G--FGLMRTD--ND---GNIREFKEK--PSG---EALKA--MAVDTSRFGLSPDS-AKERP--YLASM--G---IYVFSR--STL
136  LPV--E--EKAAS--G--FGLLKVD--GT---GRVTDFREK--PTG---DALRD--MRVDTTRYGLTIEE-AHRKP--YIASM--G---IYVFKR--QVL
```

```
137  VPI--D--EERAS--S--FGLMKID--DH---GRVVDFSEK--PKG---DELKQ--MQVDTTVLGLTPEQ-AKESP--YIASM--G---IYVFKK--EVL
138  IPI--D--ERRAS--D--FGLMKID--DS---GRIIDFSEK--PKG---EALTQ--MQVDTSVLGLTKEQ-AQKQP--YIASM--G---IYVFKK--EVL
139  LPV--D--AEQAE--G--FGLMRTD--SD---GNIQEFREK--PKG---ESLKA--MAVDTSRFGLSAES-AKNKP--YLASM--G---IYVFSR--ATL
140  LPC--E--EKVAS--G--FGLLKLG--EN---GRIVDFKEK--PTG---DLLKA--CQVDTQALGLSPEE-AKAKP--YIASM--G---IYVFKR--EAL
141  LPC--E--EKVAS--G--FGLLKID--AD---GRIVDFKEK--PQG---ELLKA--CQVDTQALGLSPEE-AKAKP--YIASM--G---IYVFRR--EAL
142  LPV--D--AEQAE--G--FGLMRTD--SD---GNIQEFREK--PKG---ESLKA--MAVDTSRFGLSAES-ARNKP--YLASM--G---IYVFSR--ATL
143  LPV--D--PKQAE--A--FGLMRTD--GD---GDIKEFREK--PKG---DSLLE--MAVDTSRFGLSANS-AKERP--YLASM--G---IYVFSR--DTL
144  IPI--D--DRRAS--D--FGLMKID--NS---GRVIDFSEK--PKG---EALTK--MRVDTTVLGLTPEQ-AASQP--YIASM--G---IYVFKK--DVL
145  LPV--D--SSQAE--A--FGLMRTD--GE---GNIKEFREK--PTG---DSLKA--MAVDTSRFGLTAQS-AKERP--YLASM--G---IYVFSR--ATL
146  LPV--D--EAQAE--G--FGLMRTD--DL---GNIKEFSEK--PTG---EKLKA--MAVDTSKFGLSKES-AAEKP--YLASM--G---IYVFSR--NTL
147  LPV--D--SAQAE--A--FGLMRTD--EA---GNIKEFREK--PTG---DSLKA--MAVDTSRFGLEANE-AKEKP--YLASM--G---IYVFSR--STL
148  LPV--D--EAQAE--G--FGLMRTD--DV---GNIKEFSEK--PSG---EKLKA--MAVDTSKFGLSKES-AAEKP--YLASM--G---IYVFSR--NTL
149  LPV--D--EAQAE--G--FGLMRTD--DL---GNIKEFSEK--PTG---EKLKS--MAVDTSKFGLTKES-ALEKP--YLASM--G---IYVFSR--KTL
150  LPV--D--AAQAE--A--FGLMRTD--EV---GNIKEFREK--PKG---DSLKA--MAVDTSRFGLSVES-SKERP--YLASM--G---IYVFSR--KTL
151  VPI--D--EKRAS--S--FGLMKID--DN---ARVVDFSEK--PKG---EALRQ--MQVDTSILGLSPDQ-ARKNP--YIASM--G---IYIFNR--EVL
152  LPV--D--EAQAE--G--FGLMRTD--DL---GNIKEFSEK--PTG---EKLKA--MAVDTSKFGLTKES-AAEKP--YLASM--G---IYVFSR--NTL
153  VPV--D--DRKAP--E--LGLMKID--AQ---GRITDFSEK--PQG---EALRA--MQVDTSVLGLSAEK-AKLNP--YIASM--G---IYVFKK--EVL
154  VPI--D--EERAS--S--FGLMKID--DH---GRVVDFSEK--PKG---DELKQ--MQVDTTVLGLTPEQ-AKESP--YIASM--G---IYVFKK--EVL
155  LPI--D--EKRAS--D--FGLMKLD--GS---GRVVEFSEK--PKG---DELRA--MQVDTTILGLDPVA-AAAQP--FIASM--G---IYVFKR--DVL
156  LPV--E--EQVAS--S--FGLLQVD--HS---GRVTAFSEK--PQG---EALTR--MRVDTTDFGLTPAE-AAHKP--YLASM--G---IYVFNR--QVL
157  LPI--D--EKRAS--D--FGLMKLD--GS---GRVVEFSEK--PKG---DELRA--MQVDTTILGLDPVA-AAAQP--FIASM--G---IYVFKR--DVL
158  LPV--D--PKQAE--A--FGLMRTD--EN---GSIKEFREK--PKG---DSLLE--MSVDTSRFGLSVES-AKERP--YLASM--G---IYVFSR--QTL
159  LPV--D--AKQAE--S--FGLMRTD--SD---GNIQEFREK--PKG---DSLRE--MAVDTSRFGLTPES-AQERP--YLASM--G---IYVFSR--DTL
160  VPM--D--ERRAS--A--FGLMKLD--ES---GRVGDFCEK--PSG---DELTQ--MQVDTTLLGLSAEQ-AREQP--YIASM--G---IYVFKK--EVL
161  LPI--D--AYRAP--A--FGLMKID--ESS--GRVVDFSEK--PQG---EELER--MKVDTTTLGLTPEE-AQEKP--FIASM--G---IYVFKK--DVL
162  LPI--D--EARAS--E--FGVMKID--NS---GRIVEFSEK--PKG---NALKA--MAVDTSILGVSPEI-ATKQP--YIASM--G---IYVFNK--DAM
163  IPI--D--DRRAS--D--FGLMKID--NS---GRVIDFSEK--PKG---EALTK--MRVDTTVLGLTPEQ-AASQP--YIASM--G---IYVFKK--DVL
164  LPV--D--EKQAS--S--FGLMKID--NT---GRIIDFQEK--PKG---DDLKR--MEVDTKTLGLSAQE-AKMKP--YIASM--G---IYLFKR--EVL
165  VPI--D--EKRAS--S--FGLMKID--DN---GRIVDFSEK--PKG---EELKQ--MQVDTSILGLNPEQ-AKESP--YIASM--G---IYVFNK--KAL
166  VPV--G--EKVAP--A--FGLMKID--AN---GRVVDFSEK--PTG---EALKA--MQVDTQSLGLDPEQ-AKEKP--YIASM--G---IYVFKK--QVL
167  LPV--D--EGQAE--G--FGLMRTD--DL---GNIKEFSEK--PTG---KKLKA--MAVDTSKFGLSKYS-AAEKP--YLASM--G---IYVFSR--NTL
168  LPV--D--PAQAE--A--FGLMRTD--EI---GNIKEFREK--PTG---DSLKA--MAVDTSRFGLEANE-AKEKP--YLASM--G---IYVFSR--STL
169  LPV--D--REQAQ--S--FGLMHTG--AE---ASITKFREK--PKG---EALDE--MSCDTASMGLSPEE-AHRRP--FLASM--G---IYVFKR--DVL
170  LPV--D--AKQAE--A--FGLMRTD--ED---GRILEFREK--PKG---DSLLE--MAVDTSRFGLSAES-AKERP--YLASM--G---IYVFSR--DTL
171  VPI--D--ERRAS--A--FGVMKIN--DS---GRIVDFYEK--PKG---AELER--MRVDTTILGLSPDQ-ARQSP--YIASM--G---IYVFKK--NVL
172  LPV--D--EGQAE--G--FGLMRTD--DL---GNIKEFSEK--PTG---EKLKA--MAVDTSKFGLSKDS-AAKKP--YLASM--G---IYVFSR--NTL
173  LPV--D--PVQAE--A--FGLMRTD--GE---GHIQEFREK--PKG---EALEA--MRVDTQSLGLSPEE-AAKRP--HLASM--G---IYVFSR--DTL
174  VPI--D--EKRAS--S--FGLMKID--DN---GRVIDFSEK--PKG---DALKQ--MQVDTTILGLSPDQ-ARKSP--YIASM--G---IYVFKK--DVL
175  IPI--D--DYRAS--D--FGLMKID--NS---GRVIDFSEK--PKG---EALAQ--MRVDTTVLGLTKEQ-AELQP--YIASM--G---IYVFKK--DVL
176  VPI--D--ETRAS--S--FGLMQIN--DR---GKVIDFREK--PTG---ELLKQ--MQVDTTVLGLTPEE-ARNSP--YIASM--G---IYVFSK--AVM
177  IPI--D--ERRAS--D--FGLMKIN--ES---GRVVDFSEK--PKG---EALKK--MRVDTTVLGLNQEQ-AEQQP--YIASM--G---IYVFKK--DVL
178  VPI--D--EKRAS--S--FGLMKID--DN---GRIVDFSEK--PKG---EELKQ--MQVDTSILGLNPEQ-AKESP--YIASM--G---IYVFNK--KAL
179  LPV--D--EKQAS--S--FGLMKID--NT---GRIIDFQEK--PKG---DDLKR--MEVDTKTLGLSAQE-AKMKP--YIASM--G---IYLFKR--EVL
180  LPV--D--AAQAE--A--FGLMRTD--ND---GNIKEFREK--PKG---DSLKE--MAVDTSRFGLSAES-SKERP--YLASM--G---IYVFSR--KTL
181  VPV--N--KTRAS--C--LGLTKIN--NQ---GKVIRFFEK--PSE---NELNQ--MQCKSSILGLSKEQ-AIKKP--YMASM--G---IYVFNK--KVL
182  LPM--D--EKRAS--D--FGLMKID--DK---GRIVSFSEK--PKG---DALKQ--MQVDTTTLGLTPQQ-AQESP--YIASM--G---IYVFKK--EVL
183  LPM--D--DKRAS--D--FGLMKTD--ED---GRIVSFSEK--PKG---EALKE--MQVDTTKLGLTAEQ-AKESP--YIASM--G---IYVFNK--DVL
184  IPI--D--GRRAS--D--FGLMKID--SA---GRVIDFSEK--PKG---EALAK--MQVDTTVLGLTSEE-ARSQP--YIASM--G---IYVFKK--DVL
185  IPI--D--GRRAS--D--FGLMKID--NG---GRVIDFSEK--PKG---EALAK--MQVDTTILGLTSEE-ARSQP--YIASM--G---IYVFKK--DVL
186  LPV--D--EKRAS--A--FGLMKID--EST--GRIIDFSEK--PKG---EALKQ--MAVDTSSLGLSPEE-AAESP--YIASM--G---IYVFKK--DVL
187  VPI--D--ERRAS--S--FGLMKID--DS---GRVVDFSEK--PKG---DALKQ--MQVDTSILGLNPEQ-AKESP--YIASM--G---IYVFNK--KAL
188  LPV--D--PKQAE--A--FGLMRTD--EN---GSIKEFREK--PKG---DSLLE--MAVDTSRFGLSADS-AKERP--YLASM--G---IYVFSR--KTL
189  LPM--D--GERAS--D--FGLMKID--KT---GRITEFAEK--PEG---NDLLA--MQVDTTVLGLSPEE-SQASP--YIASM--G---IYVFKK--SAL
190  VPM--D--AARAE--A--FGLMKID--DS---GRIIDFAEK--PKG---KELEA--MAVDTTILGLDKKL-AKEMP--YIASM--G---IYVFKA--SAM
191  LPM--D--DKRAS--D--FGLMKID--DT---GRITEFAEK--PNG---DALKA--MEVDTTILGLTAEE-ATSSP--YIASM--G---IYVFKK--SAL
192  VPM--D--EERAA--A--FGLMKID--DT---GKIIDFAEK--PTG---DALKA--MMVDTTILGLDAER-AKEMP--YIASM--G---IYVFNA--RAM
193  IAY--G--SDRAK--E--FGLMKID--EK---RRVTSFAEK--PKTQ--EALDA--MKVDTTVLGLTPEE-AAEKP--YIASM--G---IYVFKK--SVL
194  LPC--A--EKEAS--A--FGLMKID--EE---GRVIEFAEK--PKG---EALTK--MRVDTGILGVDPAT-AAAKP--YIASM--G---IYVMSA--KAL
195  LPI--D--EARAS--D--FGLMKID--ST---GRIVEFTEK--PKG---DALQA--MKVDTTVLGLTADE-AKEKP--FIASM--G---IYVFKK--SAL
196  LPC--D--EKRAS--S--FGLMKID--NT---GRVIEFAEK--PKG---AELQA--MKVDTTVLGLDADK-AQEMP--FIASM--G---IYVFDA--KKM
197  LPC--A--EKEAS--A--FGLMKID--DA---GRVIEFAEK--PKG---EALQR--MKVDTSILGVDPAT-AQSKP--FIASM--G---IYVMSA--KAL
198  IAY--G--SDRAK--E--FGLMKID--DK---RRVLSFAEK--PKTQ--EALDA--MKVDTTVLGLTPDE-AADKP--YIASM--G---IYVFKK--SVL
199  LPI--D--EERAS--D--FGLMKID--SS---GRIVEFTEK--PKG---DALQA--MKVDTTILGLTAAE-AEAKP--FIASM--G---IYVFKK--SML
200  LPT--D--EKRAS--S--FGLMKIN--EH---ATIIEFSEK--PKG---DALKA--MQCDTTILGLDAER-AKEMP--YIASM--G---IYVFNA--KAM
201  VPM--D--DSRAS--D--FGLMKID--AN---GQILYFSEK--PKG---ADLKA--MQVDTTVLGLTPEE-AIEKP--YIASM--G---IYVFKK--DIL
203  LPM--D--EKRAT--A--FGLMKID--DE---GRITEFSEK--PKG---SALKA--MEVDTTILGLDPER-AKEMP--YIASM--G---IYVVSK--DVM
204  LPM--D--EKRAT--A--FGLMKID--DE---GRITEFSEK--PKG---SALKA--MEVDTTILGLDPER-AKEMP--YIASM--G---IYVVSK--DVM
206  VPV--D--ESRAS--D--FGLVKTD--AR---GRIISFSEK--PKG---MDLKA--MQVDTTALGLSREE-AKKMP--YIASM--G---IYVFRK--DVL
207  VPV--D--ESRAS--D--FGLVKTD--AR---GRIISFSEK--PKG---MDLKA--MQVDTTALGLSREE-AKKMP--YIASM--G---IYVFRK--DVL
208  VPM--D--DSRAS--D--FGLMKID--AN---GQILYFSEK--PKG---ADLKA--MQVDTTVLGLTPEE-AIEKP--YIASM--G---IYVFKK--DIL
209  VPM--D--DSRAS--D--FGLMKID--AN---GQILYFSEK--PKG---ADLKA--MQVDTTVLGLTPEE-AIEKP--YIASM--G---IYVFKK--DIL

               310       320       330       340       350       360       370       380       390       400
     ....|....|....|....|....|....|....|....|....|....|....|....|....|....|....|....|....|....|....|....|
1    LNLLRD--KFPGANDFGSE--VIPGATS---LGM----RVQAYLY---DGYW--EDI--GT--IEAFYNANLGI--TKKPVPDFSFYDRSAPIYTQPRYL
2    LNLLRD--KFPGANDFGSE--VIPGATS---LGM----RVQAYLY---DGYW--EDI--GT--IEAFYNANLGI--TKKPVPDFSFYDRSAPIYTQPRYL
3    LNLLRD--KFPGANDFGSE--VIPGATS---LGM----RVQAYLY---DGYW--EDI--GT--IEAFYNANLGI--TKKPVPDFSFYDRSAPIYTQPRYL
```

```
4    LNLLRD--KFPGANDFGSE--VIPGATS---LGM----RVQAYLY---DGYW--EDI--GT--IEAFYNANLGI--TKKPVPDFSFYDRSAPIYTQPRYL
5    LDLLRN--QFPGANDFGSE--VIPGATS---LGL----RVQAYLY---DGYW--EDI--GT--IEAFYNANLGI--TKKPVPDFSFYDRSAPIYTQPRYL
6    LELLRD--KFPGANDFGSE--VIPGATS---LGL----RVQAYLY---DGYW--EDI--GT--IEAFYNANLGI--TKKPVPDFSFYDRSAPIYTQPRYL
7    LDLLRD--QFPGANDFGSE--VIPGATS---IGK----RVQAYLY---DGYW--EDI--GT--IEAFYNANLGI--TKKPVPDFSFYDRSSPIYTQPRYL
8    LDLLRD--KFPGANDFGSE--VIPGATE---LGM----RVQAYLY---DGYW--EDI--GT--IEAFYNANLGI--TKKPVPDFSFYDRSSPIYTQPRYL
9    LDLLRE--KFPGANDFGSE--VIPEYVR--HVCVYS--QVQAYLY---DGYW--EDI--GT--IEAFYNANLGI--TKKPVPDFSFYDRSSPIYTQPRYL
10   LDLLRE--KFPGANDFGSE--VIPGATS---IGMR---NVQAYLY---DGYW--EDI--GT--IEAFYNANLGI--TKKPVPDFSFYDRSSPIYTQPRYL
11   LDLLRE--KFPGANDFGSE--VIPGATS---IGM----RVQAYLY---DGYW--EDI--GT--IEAFYNANLGI--TKKPVPDFSFYDRSSPIYTQPRYL
12   LDLLRD--KFPGANDFGSE--VIPGATS---IGL----RVQAYLY---DGYW--EDI--GT--IEAFYNANLGI--TKKPIPDFSFYDRSSPIYTQPRYL
13   LDLLRD--QFPGANDFGSE--VIPGATS---LGL----RVQAYLY---DGYW--EDI--GT--IEAFYNANLGI--TKKPVPDFSFYDRSSPIYTQPRYL
14   LDLLRD--QFPGANDFGSE--VIPGATD---LGL----RVQAYLY---DGYW--EDI--GT--IEAFYNANLGI--TKKPVPDFSFYGRSAPIYTQPRYL
15   LNLLRE--QFPGANDFGSE--VIPGATS---IGL----RVQAYLY---DGYW--EDI--GT--IEAFYNANLGI--TKKPVPDFSFYDRSSPIYTQPRYL
16   LDLLRD--KFPGANDFGSE--VIPGATE---LGL----RVQAYLY---DGYW--EDI--GT--IEAFYNANLGI--TKKPVPDFSFYDRSSPIYTQPRYL
17   LDLLRD--KFPGANDFGSE--VIPGATS---VGM----RVQAYLY---DGYW--EDI--GT--IEAFYNANLGI--TKKPVPDFSFYDRSSPIYTQPRYL
18   LDLLRE--KFPGANDFGSE--VIPGATN---IGM----RVQAYLY---DGYW--EDI--GT--IEAFYNANLGI--TKKPVPDFSFYDRSSPIYTQPRYL
19   LDLLSD--KFPGANDFGSE--VIPGATS---IGM----RVQAYLY---DGYW--EDI--GT--IEAFYNANLGI--TKKPVPDFSFYDRSSPIYTQPRYL
20   LSLLRD--KFPGANDFGSE--VIPGATS---IGM----RVQAYLY---DGYW--EDI--GT--IEAFYNANLGI--TKKPVPDFSFYDRSSPIYTQPRYL
21   LNLLRD--KFPGANDFGSE--VIPGATS---IGM----RVQAYLY---DGYW--EDI--GT--IEAFYNANLGI--TKKPVPDFTFYDRSSPIYTQPRYL
22   LSLLRD--KFPGANDFGSE--VIPGATS---IGM----RVQAYLY---DGYW--EDI--GT--IEAFYNANLGI--TKKPIPDFSFYDRSDPIYTQPRYL
23   LNLLRD--KFPGANDFGSE--VIPGATS---IGM----RVQAYLY---DGYW--EDI--GT--IEAFYNANLGI--TKKPIPDFSFYDRSAPIYTQPRYL
24   LDLLRE--KFPGANDFGSE--VIPGATS---IGL----RVQAYLY---DGYW--EDI--GT--IEAFYNANLGI--TKKPIPDFSFYDSSSPIYTQPRYL
25   VNLLRQ--KFPGANDFGSE--VIPGATS---IGL----RVQAYLF---DGYW--EDI--GT--IEAFYNANLGI--TKKPVPDFSFYDRSAPIYTQPRYL
26   LNLLRE--KFPGANDFGSE--VIPGATS---IGM----RVQAYLF---DGYW--EDI--GT--IEAFYNANLGI--TKKPVPDFSLYDRSAPIYTQPRYL
27   LNLLRD--KFPGANDFGSE--VIPGATS---VGL----RVQAYLY---DGYW--EDI--GT--IEAFYNANLGI--TKKPVPDFSFYDRSAPINTQPRYL
28   LNLLRD--EFPAANDFGSE--VIPGATA---MGL----RVQAYLF---DGYW--EDI--GT--IEAFYNANLGI--TKKPVPDFSFYDRSAPIYTQPRYL
29   LNLLRE--KFPAANDFGSE--VIPGATS---IGL----RVQAYLY---DGYW--EDI--GT--IEAFYNANLGI--TKKPVPDFSFYGRSSPIYTQPRYL
30   LDLLRD--KFPGANDFGSE--VIPGATE---LGM----RVQAYLY---DGYW--EDI--GT--IEAFYNANLGI--TKKPVPDFSFYDRSSPIYTQPRYL
31   LDLLRD--KFPGANDFGSE--VIPGATS---IGM----RVQAYLY---DGYW--EDI--GT--IEAFYNANLGI--TKKPVPDFSFYDRSSPIYTQPRYL
32   LRLLRE--NFPAANDFGSE--VIPGATE---IGL----RVQAYLY---DGYW--EDI--GT--IEAFYNANLGI--TKKPVPDFSFYDRSAPIYTQPRYL
33   LQLLRE--QFPEANDFGSE--VIPGATS---IGK----RVQAYLY---DGYW--EDI--GT--IAAFYNANLGI--TKKPIPDFSFYDRFAPIYTQPRHL
34   LQLLRE--QFPEANDFGSE--VIPGATS---IGK----RVQAYLY---DGYW--EDI--GT--IAAFYNANLGI--TKKPMPDFSFYDRFAPIYTQPRHL
35   LQLLRE--QFPEANDFGSE--VIPGATS---IGK----RVQAYLY---DGYW--EDI--GT--IAAFYNANLGI--TKKPMPDFSFYDRFAPIYTQPRHL
36   LQLLRE--QFPGANDFGSE--VIPGATS---IGK----RVQAYLY---DGYW--EDI--GT--IEAFYNANLGI--TKKPIPDFSFYDRSAPIYTQPRHL
37   LQLLRE--QFPGANDFGSE--VIPGATN---IGM----RVQAYLY---DGYW--EDI--GT--IEAFYNANLGI--TKKPVPDFSFYDRSAPIYTQPRHL
38   LKLLRQ--NFPAANDFGSE--VIPGATE---IGM----RVQAYLY---DGYW--EDI--GT--IEAFYNANLGI--TKKPVPDFSFYDRSAAIYTQPRYL
39   LQLLRE--QFPGANDFGSE--VIPGATN---IGM----RVQAYLY---DGYW--EDI--GT--IEAFYNANLGI--TKKPVPDFSFYDRSAPIYTQPRHL
41   LQLLRE--QFPGANDFGSE--VIPGATS---TGM----RVQAYLY---DGYW--EDI--GT--IEAFYNANLGI--TKKPIPDFSFYDRSAPIYTQPRHL
42   LQLLRE--QFPGANDFGSE--VIPGATS---TGM----RVQAYLY---DGYW--EDI--GT--IEAFYNANLGI--TKKPIPDFSFYDRSAPIYTQPRHL
43   LRLLRE--NFPAANDFGSE--VIPGATE---IGL----RVQAYLY---DGYW--EDI--GT--IEAFYNANLGI--TKKPVPDFSFYDRSAPIYTQPRYL
44   LRLLRD--NFPSANDFGSE--VIPGATE---IGM----RVQAYLY---DGYW--EDI--GT--IEAFYNANLGI--TKKPVPDFSFYDRSAPIYTQSRYL
45   LQLLRE--QFPGANDFGSE--VIPGATS---TGM----RVQAYLY---DGYW--EDI--GT--IEAFYNANLGI--TKKPIPDFSFYDRSAPIYTQPRHL
46   LQLLRE--QFPGANDFGSE--VIPGATS---TGM----RVQAYLY---DGYW--EDI--GT--IEAFYNANLGI--TKKPIPDFSFYDRSAPIYTQPRHL
47   LRLLRD--NFPSANDFGSE--VIPGATE---IGM----RVQAYLY---DGYW--EDI--GT--IEAFYNANLGI--TKKPVPDFSFYDRSAPIYTQSRYL
48   LQLLRE--QFPGANDFGSE--VIPGATS---TGM----RVQAYLY---DGYW--EDI--GT--IEAFYNANLGI--TKKPIPDFSFYDRSAPIYTQPRHL
49   LKLLKW--SYPTSNDFGSE--IIPAAID---D-Y----NVQAYIF---KDYW--EDI--GT--IKSFYNASLAL--TQEF-PEFQFYDPKTPFYTSPRFL
50   LNLLKS--AYPSCNDFGSE--IIPSAVK---D-H----NVQAYLF---NDYW--EDI--GT--VKSFFDANLAL--TKQP-PKFDFNDPKTPFYTSARFL
51   LNLLRW--RFPTANDFGSE--IIPASTK---E-F----CVKAYLF---NDYW--EDI--GT--IRSFFRANLAL--TEHP-PRFSFYDATKPIYTSRRNL
52   LNLLRW--RFPTANDFGSE--IIPFSAK---E-F----YVNAYLF---NDYW--EDI--GT--IRSFFEANLAL--TEHP-GAFSFYDAAKPIYTSRRNL
53   LKLLRS--SYPTSNDFGSE--IIPLAVG---E-H----NVQAFLF---NDYW--EDI--GT--IGSFFDANLAL--TEQP-PKFQFYDQKTPFFTSPRFL
54   LKLLTW--RYPSSNDFGSE--IIPAAIK---D-H----NVQGYIY---RDYW--EDI--GT--IKSFYEANIAL--VEEH-PKFEFYDQNTPFYTSPRFL
55   LNLLTR--QYPSSNDFGSE--VIPAAIR---D-H----DVQGYIF---RDYW--EDI--GT--IKTFYEANLAL--VEER-PKFEFYDPETPFYTSPRFL
56   LKLLKW--SYPTSNDFGSE--IIPAAID---D-Y----NVQAYIF---KDYW--EDI--GT--IKSFYNASLAL--TQEF-PEFQFYDPKTPFYTSPRFL
57   LKLLKS--AYPSCNDFGSE--IIPSAVK---D-H----NVQAYLF---NDYW--EDI--GT--VKSFFDANLAL--TKQP-PKFDFNDPKTPFYTSARFL
58   LNLLRW--RFPTVNDFGSE--IIPASTK---E-F----CVKAYYL--FNDYW--EDI--GT--IRSFFEANLAL--TEHP-PRFSFYDATKPIYTSRRNL
59   LKLLKW--SYPTSNDFGSE--IIPAAID---D-Y----NVQAYIF---KDYW--EDI--GT--IKSFYNASLAL--TQEF-PEFQFYDPKTPFYTSPRFL
60   LNLLKS--AYPSCNDFGSE--IIPSAVK---D-H----NVQAYLF---NDYW--EDI--GT--VKSFFDANLAL--TKQP-PKFDFNDPKTPFYTSARFL
61   LNLLRW--RFPTANDFGSE--IIPFSAK---E-F----YVNAYLF---NDYW--EDI--GT--IRSFFEANLAL--TEHP-GAFSFYDAAKPIYTSRRNL
62   LKLLRS--SYPTSNDFGSE--IIPLAVR---E-H----NVQAFLF---NDYW--EDI--GT--IGSFFDANLAL--TEQP-PKFQFYDPKTPFFTSPRFL
63   LKLLTW--RYPSSNDFGSE--IIPAAIR---D-H----NVQGYIY---RDYW--EDI--GT--IKSFYEANLAL--VEEH-PKFEFYDQNTPFYTSPRFL
64   LNLLTR--QYPSSNDFGSE--VIPAAIR---D-H----DVQGYIF---RDYW--EDI--GT--IKTFYEANLAL--VEER-PKFEFYDPDTPFYTSPRFL
66   LKVLRG--CYPNANDFGSE--VIPMAAK---D-F----NVQACLF---NGYW--EDI--GT--IKSFFDANLAL--MDQR-PKFQLYDQSKPIFTCPRFL
67   LNLLRW--RFPTANDFGSE--VIPASAR---E-F----YMKAYLF---NDYW--EDI--GT--IRSFFEANLAL--TEHP-PRFSFYDAAKPMYTSRRNL
68   LKLLRW--SCPSCNDFGSE--IIPSALR---D-H----KVQAYMF---RDYW--KDI--GT--IKSFFEANLEL--TKQS-PNFEFYDQETPFFTSPRFL
69   LKLLRW--SCPSCNDFGSE--IIPSALR---D-H----KVQAYMF---RDYW--KDI--GT--IKSFFEANLEL--TKQS-PNFEFYDQESPFFTSPRFL
70   LKLLKW--RYPTSNDFGSE--IIPAAVR---E-N----NVQAYFF---NDYW--EDI--GT--IKSFYDANLAL--TEEN-PMFKFYDPKTPIYTSPRFL
71   LRLLKW--RYPTSNDFGSE--IIPAAVR---E-N----NVQAYFF---IDYW--EDI--GT--IKSFYDANLAL--TEEN-PMFKFYDPKTPIYTSPRFL
72   LNLLRW--RFPTANDFGSE--VIPASAR---E-F----YMKAYLF---NDYW--EDI--GT--IRSFFEANLAL--TEHP-PRFSFYDAAKPMYTSRRNL
73   LQLLRW--KCSSCNDFGSE--IIPSAVN---E-H----NVQAYLF---NDYW--EDI--GT--IKSFFDANLAL--TEQP-PKFEFYDPKTPFFTSPRFL
74   LNLLKW--RYPTSNDFGSE--IIPAAVR---D-H----NVQSYFF---GDYW--EDI--GT--IKSFYNANLAL--TEES-HKFEFYDPKIPIYTSPGFL
75   LQLLRW--NGSSCNDFGSE--IIPSAVN---E-H----NVQAYLF---NDYW--EDI--GT--IKSFFDANLAL--TEQP-PKFEFYDPKTPFFTSPRFL
76   LNLLKW--RYPTSNDFGSE--IIPAAVR---D-H----DVQSYFF---EDYW--EDI--GT--IKSFYDANLAL--TEES-HKFEFYDPKIPIYTSPGFL
77   RKVLRG--CYPNANDFGSE--VIPMAAK---D-F----NVQACLF---NGYW--EDI--GT--IKSFFDANLAL--MDQR-PKFQLYDQSKPIFTCPRFL
80   LKLLKW--RYPTSNDFGSE--IIPASVK---E-Y----NVQAFFF---GDYW--EDI--GT--IKSFYDANMAL--TEES-PMFKFYDPKTPIFTSPGFL
81   LKLLRW--NYPTANDFGSE--IIPMSTK---E-Y----NVQAYLF---NGYW--EDI--GT--IKSFFDANLAL--TDQP-PNFHFFDPLKPIFTSPRFL
82   LKLLRW--RYPTANDFGSE--IIPAAVM---E-H----NVQAYIF---KDYW--EDI--GT--IKSFYEANLAL--AEEP-PKFEFYDPKTPFYTSPRFS
84   LNLLRW--RFPTANDFGSE--IIPASAK---E-F----YMKAYLF---NDYW--EDI--GT--IRSFFAANLAL--TEHP-PRFSFYDAAKPMYTSRRNL
87   LNLLRW--RFPTANDFGSE--IIPASAK---E-F----FIKAYLF---NDYW--EDI--GT--IQSFFAANLAL--TEHP-PRFSFYDAAKPMYTSRRNL
```

```
88   LKLLRW--HYPTANDFGSE--IIPLSAK---D-Y----NVRAYLF---NDYW--EDI--GT--IKSFFDSNLAL--TDQP-PEFQFFDPLKPIFTSPRFL
89   LKLLRW--RFPTSNDFGSE--IIPAAVM---E-H----NIQSYNF---RDYW--EDI--GT--IKSFYEANLAL--TEEP-PTFEFYDPKTPFYTSPRFL
90   CKLLRW--SYPSCIDFGSE--VIPYAVK---D-H----NVQAYLF---NDYW--EDI--GT--IKSFFDANLAL--TEQP-PKFEFYDPKTPFFTSPRFL
91   LKLLTR--KYLSCNDFGSE--IIPLAVK---D-H----NVQAYLF---NDYW--EDI--GT--IKSFFDANLAL--TEQP-PKFEFYDPKTPFYTSPRFL
92   LKLLRW--SYPTANDFGSE--VIPMAAE---E-C----NVQAYLF---NGYW--EDI--GT--IKSFFDANLAL--TDQP-PKFHFYDPLKPIFTSPRFL
94   LNLLRW--RFPTANDFGSE--IIPASAK---E-F----FIKAYLF---NDYW--EDI--GT--IRSFFEANLAL--TAHP-PRFSFYDATKPMYTSRRNL
95   LNLLRW--RYPTSNDFGSE--IIPLAVM---E-H----NVEAFLF---RDYW--EDI--GT--IKTFYEANMGL--TEEF-PKFEFYNPKTPIFTSPRFL
96   LKLLRW--SYPSCNDFGSE--IIPSAVR---D-H----NVQAYLF---NDYW--EDI--GT--VKSFFDANLGL--TKQP-PKFEFYDPQTPFFTSPRFL
97   LKLLRW--RFPTSNDFGSE--IIPAAVM---E-H----NVQAYIF---KDYW--EDI--GT--IKSFYEANLAL--AEEP-PKFEFYDPKTPFYTSPGFL
98   LKLLRC--SYPSCNDFGSE--IIPSAVK---E-H----NVQAYLF---NDYW--EDI--GT--IKSLFDANLAL--TEQP-PKFEFYDPKTPFFTSPRFL
99   LNLLRW--RFPTANDFGSE--IIPASAK---E-I----DVKAYLF---NDYW--EDI--GT--IKSFFEANLAL--AEQP-PRFSFYDADKPMYTSRRNL
100  LDLLKS--KYTQLHDFGSE--ILPRAVL---D-H----SVQACIF---TGYW--EDV--GT--IKSFFDANLAL--TEQP-SKFDFYDPKTPFFTAPRCL
101  LDLLKS--RYAELHDFGSE--ILPKALH---E-H----NVQAYVF---TDYW--EDI--GT--IRSFFDANMAL--CEQP-PKFEFYDPKTPFFTSPRYL
102  RKLLRG--HYPTANDFGLE--VIPMAAK---D-Y----DVQAYLF---DGYW--EDI--GT--IKSFFEANLAL--TDQS-PNFYFYDPVKPIFTSPRFL
103  LNLLKS--RYAELHDFGSE--ILPRALH---E-H----NVQAYVF---TDYW--EDI--GT--IRSFFDANMAL--CEQP-PKFEFYDPKTPFFTSPRYL
104  LRLLRG--HYPTANDFGSE--VIPMAAK---D-Y----NVQAYLF---DGYW--EDI--GT--IKSFFEANLAL--TDQS-PNFHFYEPVKPIFTSPRFL
105  LNLLRW--RFPTANDFGSE--IIPAAAK---E-I----NVKAYLF---NDYW--EDI--GT--IKSFFEANLAL--AEQP-PRFSFYDASKPMYTSRRNL
106  LNLLRW--RFPTANDFGSE--IIPASAK---E-I----NVKAYLF---NDYW--EDI--GT--IKSFFEANLSL--AEQP-PRFSFYDANKPMYTSRRNL
107  LNLLKS--RYAELHDFGSE--ILPRALH---E-H----NVQAYVF---ADYW--EDI--GT--IRSFFDANMAL--CEQP-PKFEFYDPKTPFFTSPRYL
108  LRLLRG--HYPTANDFGSE--VIPMAAK---D-Y----NVQAYLF---DGYW--EDI--GT--IKSFFEANLAL--TDQS-PNFYFYDPVKPIFTSPRFL
109  LDILKS--KYAHLQDFGSE--ILPRAVL---E-H----NVKACVF---TEYW--EDI--GT--IKSFFDANLAL--TEQP-PKFEFYDPKTPFFTSPRYL
110  LDLLKS--RYAELHDFGSE--ILPKALH---E-H----NVQAYVF---TDYW--EDI--GT--IRSFFDANMAL--CEQP-PKFEFYDPKTPFFTSPRYL
111  LDLLKS--KYTQLHDFGSE--ILPRAVL---E-H----NVQTCIF---MGYW--EDV--GT--IKSFFDANLAL--TEQP-SKFDFYDPKTPFFTAPRYL
112  LNLLRW--RFPTANDFGSE--IIPAAAK---E-I----NVKAYLF---NDYW--EDI--GT--IKSFFEANLAL--AEQP-PRFSFYDADKPMYTSRRNL
113  LNLLKS--RYAELHDFGSE--ILPRALH---D-H----NVQAYVF---TDYW--EDI--GT--IRSFFDANMAL--CEQP-PKFEFYDPKTPFFTSPRYL
114  LNLLRW--RFPTANDFGSE--IIPAAAR---E-I----NVKAYLF---NDYW--EDI--GT--IKSFFEANLAL--AEQP-SKFSFYDASKPMYTSRRNL
115  LNLLKS--RYAELHDFGSE--ILPRALH---D-H----NVQAYVF---TDYW--EDI--GT--IRSFFDANMAL--CEQP-PKFEFYDPKTPFFTSPRYL
116  LNLLRW--RFPTANDFGSE--IIPASAK---E-L----FVKAYLF---NDYW--EDI--GT--IKSFCEANLSL--TRHP-PNFSFYDATKPIYTSRRNL
117  LNLLKS--RYAELHDFGSE--ILPRALH---D-H----NVQAYVF---TDYW--EDI--GQ--SDPSFDANMAL--CEQP-PKFEFYDPKTPFFTSPRYL
118  LNLLRW--RFPTANDFGSE--IIPAAAR---E-I----NVKAYLF---NDYW--EDI--GT--IKSFFEANLAL--AEQP-SKFSFYDASKPMYTSRRNL
119  LNLLKS--RYAELHDFGSE--ILPRALH---D-H----NVQAYVF---TDYW--EDI--GT--IRSFFDANMAL--CEQP-PKFEFYDPKTPFFTSPRYL
120  LNLLKS--RYAELHDFGSE--ILPRALH---D-H----NVQAYVF---TDYW--EDI--GT--IRSFFDANRAL--CEQP-PKFEFYDPKTPFFTSPRYL
121  IDLLND---HPQHKDFGKE--VIPEALA---GGM----TLKSYVF---DDYW--EDI--GT--IGAFYEANLAL--TQQPSPPFSFYDEDFPIYTRPRYL
122  FKLLKD---APDQTDFGKE--VIPGAAK---D-H----NVQAYLF---NDYW--EDI--GT--IEAFFEANLAL--TQQPQPAFSFYDENAPIYTRSRYL
123  IKLLKE---SLQSTDFGKE--IIPDASK---D-Y----NVQAYLF---DDYW--EDI--GT--IEAFYHANLAL--TKQPLPPFSFYDEKAPIYTRPRYL
124  NDLLKN---NPEQTDFGKE--IIPGAAK---D-Y----NLQAYLF---KGYW--EDI--GT--IEAFYEANLAL--NRQPRPRFSFYNEKAPIYTRARNL
125  IDLLKQ---QPDCTDFGKE--IIPNAIK---D-L----NIQAYLF---NDYW--EDI--GT--IEAFFNANLAL--AKQPNPSFSFYDKAAPIYTRARYL
126  FDLLAK---NPTATDFGKE--IIPEALS---RGD----NLQSFLF---DDYW--EDI--GT--IGAFYEANLAL--TDQPNPAFSFYDEQFPIYTRPRYL
127  FDLLAK---NPSATDFGKE--LIPASLE---RGD----HIQSYLF---DDYW--EDI--GT--IGAFYEANLAL--TDQPNPAFSFYEESFPIYTRPRYL
128  FDLLAQ---NPGSTDFGKE--IIPTALG---QGD----NLRAYLF---DDYW--EDI--GT--IGAFYEANLAL--TDQPRPAFSFYDEKFPIYTRPRYL
129  IKLLTE----TEQTDFGKE--ILPNAAP---D-Y----NLQAYLF---NDYW--EDI--GT--IEAFYNANLAL--TQQPQPPFSFYDEKAPIYTRSRYL
130  FDLLND---HPTYKDFGKE--VIPEALS---KGM----SLKSYVF---DDYW--EDI--GT--IGAFYEANLAL--TQQPKPPFSFYDEDFPIYTRPRYL
131  FDLLDK---HPGHKDFGKE--IIPEALK---RGD----KLQSYVF---DDYW--EDI--GT--IGAFYEANLAL--TQQPTPPFSFYDEKFPIYTRPRYL
132  FDLLHQ---NPTHKDFGKE--IIPEALA---RGD----RLKSYVF---DDYW--EDI--GT--IGAFYEANLAL--TQQPTPPFSFYDAEFPIYTRPRYL
133  LKLLRD---DPSRTDFGKE--ILPACLD---D-Y----NVQAYLF---DDYW--EDI--GT--IEAFYKANLAL--TSQNAPPFSFYHP-APIYTRPRYL
134  FDLLNK---FPSYTDFGKD--IIPEALS---RGD----TLKSYVF---DDYW--EDI--GT--IGAFFESNLAL--TQQPKPPFSFYDEKFPIYTRPRYL
135  FDLLNK---YPSYKDFGKE--VIPEALS---RGD----ALKSYVF---DAYW--EDI--GT--IGAFYESNLAL--TQQPTPPFSFYDEKFPIYTRARYL
136  IDLLQQ---MADATDFGKE--IIPAAAR---S-H----LVQTYLF---NGYW--EDI--GT--IGSFYEANLAL--TQQPQPPFSFYDENAPIYTRPRYL
137  AQLLEE---NPDQTDFGKE--IIPFSAK---D-Y----NLQAYLF---KGYW--EDI--GT--IKAFYEANLAL--NRQPSPRFSFYNEEYPIYTRSRYL
138  FKLLRE---SVERTDFGKE--IIPDASK---D-Y----NVQAYLF---DDYW--EDI--GT--IEAFYHANLAL--TQQPQPPFSFYDEHAPIYTRARYL
139  FDLLHK---NPSHKDFGKE--VIPEALA---RGD----RLQSYVF---DEYW--EDI--GT--IGAFYEANLAL--TQQPNPPFSFYDEKFPIYTRPRYL
140  IEMLKV----KEHTDFGKE--VLPAAIG---K-Y----HLQAYLF---KGYW--EDI--GT--IEAFYRANLAL--VQQPNPPFSFFDSEMPIYTRPRFL
141  IEMLKV----KEHTDFGKE--VLPSAIG---K-Y----HLQAYPF---KGYW--EDI--GT--IEAFYRANLAL--VQQPNPPFSFFDSEMPIYTRPRFL
142  FDLLHK---NPSHKDFGKE--VIPEALA---RGD----RLQSYVF---DEYW--EDI--GT--IGAFYEANLAL--TQQPNPPFSFYDEKFPIYTRPRYL
143  FDLLDS---NPGYKDFGKE--VIPEALK---RGD----KLKSYVF---DDYW--EDI--GT--IGAFYEANLAL--TQQPTPPFSFYDEKFPIYTRPRYL
144  IKLLKE---ALERTDFGKE--IIPDAAK---D-H----NVQAYLF---DDYW--EDI--GT--IEAFYNANLAL--TQQPMPPFSFYDEEAPIYTRARYL
145  FDLLNK---HPNYKDFGKE--VIPEALN---RGD----VLKSYVF---DDYW--EDI--GT--IGAFFESNLAL--TQQPKPPFSFYDEKFPIYTRARYL
146  FDLLNK---FPNYTDFGKD--IIPEALN---RGD----TLKSYVF---DDYW--EDI--GT--IGAFFESNLAL--TEQPKPPFSFYDEKFPIYTRPRFL
147  FDLLNK---FPSYTDFGKE--IIPEALG---RGD----KLKSYVF---NDYW--EDI--GT--IGAFFESNLAL--TQQPTPPFSFYDEKFPIYTRPRYL
148  FDLLNK---FPNYTDFGKD--IIPEALN---RGD----TLKSYVF---DDYW--EDI--GT--IGAFFESNLAL--TEQPKPPFSFYDEKFPIYTRPRFL
149  FDLLNK---FPNYTDFGKD--IIPEALG---RGD----NLKSYVF---DDYW--EDI--GT--IGAFFESNLAL--TRQPKPPFSFYDEKFPIYTRPRYL
150  FDLLDA---NPGHKDFGKE--VIPEALS---RGD----NLKSYVF---DDYW--EDI--GT--IGAFYEANLAL--TQQPTPPFSFYDEAFPIYTRPRYL
151  GKLLRQ---NPEQTDFGKE--IIPGAKT---D-Y----NLQAYLY---KGYW--EDI--GT--IEAFYESNLAL--TQQPQPPFSFYDEKAPIYTRPRYL
152  FDLLNK---FPNYTDFGKD--IIPEALK---RGD----TLKSYVF---DDYW--EDI--GT--IGAFFESNLAL--TEQPKPPFSFYDEKFPIYTRPRFL
153  HNLLEK---YEGATDFGKE--IIPDSAS---D-H----NLQAYLF---DDYW--EDI--GT--IEAFYEANLAL--TKQPSPDFSFYNEKAPIYTRGRYL
154  AQLLEE---NPDQTDFGKE--IIPFSAK---D-Y----NLQAYLF---KGYW--EDI--GT--IKAFYEANLAL--NRQPSPRFSFYNEEYPIYTRSRYL
155  IDLLSH---HPEQTDFGKE--VIPAAAT---R-Y----NTQAFLF---NDYW--EDI--GT--IASFYEANLAL--TQQPSPPFSFYDEQAPIYTRARYL
156  IDLLKQ---SPQSTDFGKE--IIPMAAT---D-H----NVQTYLF---NDYW--EDI--GT--ISSFYEANLAL--TRQPQPPFSFYDEKAPIYTRPRYL
157  IDLLSH---HPEQTDFGKE--VIPAAAT---R-Y----NTQAFLF---NDYW--EDI--GT--IASFYEANLAL--TQQPSPPFSFYDEQAPIYTRARYL
158  FDLLDK---HPGHKDFGKE--IIPEALA---RGD----KLQSYVF---DDYW--EDI--GT--IGAFYEANLAL--TQQPTPPFSFYDEKFPIYTRPRYL
159  FDLLDK---HPGHKDFGKE--IIPEALK---RGD----KLQSYVF---DDYW--EDI--GT--IGAFYEANLAL--TQQPTPPFSFYDEKFPIYTRPRYL
160  IDLLES---NIAHTDFGKE--VIPTAAA---N-H----NIQAFLF---DDYW--EDI--GT--IEAFYEANLAL--AQQPKPKFSFYDEQAPIYTRARYL
161  IDLLKN---SPDSTDFGKE--IIPSSAK---D-Y----NVQAYLF---NDYW--EDI--GT--IEAFYEANLAL--TRQPQPPFSFYDEKSPIYTRSRYL
162  IKLIED----SEDTDFGKE--ILPKSAQ---S-Y----NLQAYPF---QGYW--EDI--GT--IKSFYEANLAL--TQQPQPPFSFYDEQAPIYTRSRYL
163  IKLLKE---SLERTDFGKE--IIPDASK---D-H----NVQAYLF---DDYW--EDI--GT--IEAFYNANLAL--TQQPMPPFSFYDEEAPIYTRARYL
164  IDLLKQ---QPDCTDFGKE--IIPNAIK---D-L----NIQAYLF---NDYW--EDI--GT--IEAFFNANLAL--AKQPNPSFSFYDKAAPIYTRARYL
165  NDLLKN---NPEQTDFGKE--IIPGAAK---D-Y----NLQAYLF---KGYW--EDI--GT--IEAFYEANLAL--NRQPRPSFSFYNEKAPIYTRARNL
```

```
166   LDLLKE---GKDKTDFGKE--IIPDAAK---D-Y----NVQAYLF---DDYW--ADI--GT--IEAFYEANLGL--TKQPIPPFSFYDEKAPIYTRARYL
167   FDLLNK---FPSYTDFGKD--IIPEALN---RGD----KLKSYVF---DDYW--EDI--GT--IGAFFESNLAL--TEQPKPPFSFYDEKFPIYTRPRFL
168   FDLLNK---FPSYTDFGKE--IIPEALG---RGD----KLKSYVF---NDYW--EDI--GT--IGAFFESNLAL--TQQPTPPFSFYDEKFPIYTRPRYL
169   FRLLAE---NPGATDFGKE--IIPKALD---DGF----KLRSYLF---DDYW--EDI--GT--IRAFYEANLAL--TTQPRPPFSFYDKRFPIYTRHRYL
170   FDLLHQ---NPTHKDFGKE--VIPEALQ---RGD----RLKSYVF---DDYW--EDI--GT--IGAFYEANLAL--TQQPTPPFSFYDAEFPIYTRPRYL
171   IDLLDA---NKEQTDFGKE--IIPSAAK---D-Y----NLQAYLF---KGYW--EDI--GT--IEAFYESNLAL--TQQPNPAFSFYDEKAPIYTRSRYL
172   FDLLNK---FPSYTDFGKD--IIPEALN---RGD----SLKSYVF---DDYW--EDI--GT--IGAFFESNLAL--TEQPKPPFSFYDEKFPIYTRPRFL
173   FDLLNS---NPTATDFGKE--IIPASLA---RGD----QLRSYLF---DDYW--EDI--GT--IGAFYEANLAL--TQQPNPPFSFYDEKFPIYTRPRYL
174   GKLLRA---NLEQTDFGKE--IIPAASA---D-H----NVQAYLF---KGYW--EDI--GT--IEAFYESNLAL--TQQPYPAFSFYDEKAPIYTRARYL
175   IKLLKE---SLERTDFGKE--IIPDAAQ---D-H----NVQAYLF---DDYW--EDI--GT--IEAFYNANLAL--TQQPMPPFSFYDEAAPIYTRARYL
176   KEVLEA---NSEHTDFGNE--VIPASMP---K-Y----NIQAYLF---NDYW--QDI--GT--IEAFYNANLSL--TRQPSPSFSFYQEDAPIYTRARYL
177   IKLLKE---ASERTDFGKE--IIPDAAN---D-Y----NVQAYLF---NDYW--EDI--GT--IEAFYNANLTL--TQQPRPPFSFYDEQAPIYTRARYL
178   NDLLKN---NPEQTDFGKE--IIPGAAK---D-Y----NLQAYLF---KGYW--EDI--GT--IEAFYEANLAL--NRQPRPSFSFYNEKAPIYTRARNL
179   IDLLKQ---QPDCTDFGKE--IIPNAIK---D-L----NIQAYLF---NDYW--EDI--GT--IEAFFNANLAL--AKQPNPSFSFYDKAAPIYTRARYL
180   FDLLDA---NPGHKDFGKE--VIPEALS---RGD----VLKSYVF---DDYW--EDI--GT--IGAFYEANLAL--TQQPTPPFSFYDEAFPIYTRPRYL
181   TQLLEN---NPEQTDFGKE--VIPNAAV---Q-Y----NLQAYLF---DGYW--EDI--GT--VQAFYEANLAL--NHQPNPAFSFYNEQSPIYTHARYL
182   IKLLKE---SPNQTDFGKE--IIPASAK---D-H----NVQAYLF---DDYW--EDI--GT--IEAFYDANMAL--TKQPQPPFSFYDENAPIYTRPRFL
183   MKLLKE---SPEQTDFGKE--IIPNSAK---D-Y----NVQAYLF---DGYW--EDI--GT--IEAFYDSNLAL--TKQPHPPFSFYDEQAPIYTRQRYL
184   IKLLRE---SLEKTDFGKE--IIPDAAK---D-H----NVQAYLF---DGYW--EDI--GT--IEAFYNANLAL--TQQPVPPFSFYDEEAPIYTRARYL
185   IKLLRE---SLEKTDFGKE--IIPDAAK---D-H----NVQAYLF---DGYW--EDI--GT--IEAFYNANLAL--TQQPVPPFSFYDEEAPIYTRARYL
186   FKLLKD---APDQTDFGKE--VIPGAAK---D-H----NVQAYLF---NDYW--EDI--GT--IEAFFEANLAL--TQQPQPAFSFYDENAPIYTRSRYL
187   TDLLRN---NPEQTDFGKE--IIPGSAK---D-Y----NLQAYLF---KGYW--EDI--GT--IEAFYEANLAL--NRQPLPRFSFYNEKAPIYTRARNL
188   FDLLDK---HPGHKDFGKE--IIPEALA---RGD----KLQSYVF---DDYW--EDI--GT--IGAFYEANLAL--TQQPTPPFSFYDEKFPIYTRPRYL
189   ISFLNS--EYPKDNDFGGE--IIPKAAA---DGY----HVQAYLF---KDYW--EDI--GT--IKSFFEANLAL--AKHP-PQFEFYDARAPIYTSPRFL
190   DELLTE--KFPDCHDFGGE--IIPKANE---LGK----HVQAFLY---KGYW--EDI--GT--IEAFYNANLQC--NDPDAPKFSFYESGSPIYTQSRFL
191   LNFLNA--EYPKDNDFGGE--IIPKAAA---DGY----HVQAYLF---NDYW--EDI--GT--IKSFFEANLAL--AKNP-PQFEFYDARAPIYTSPRFL
192   EKLLME--DFPTCHDFGGE--IIPNAKD---LGM----HVQAFLY---DGYW--EDI--GT--IKAFFDANLAC--NDPEKAKFSFYQTGAPIYTQSRFL
193   LQLLND--SYAKANDFGGE--IIPSAAK---D-H----NVVAYPF---YGYW--EDI--GT--IKSFFEENLKL--CRHP-ATFEFYDPQSPIYTSPRVL
194   RELLLN--RMPGANDFGNE--VIPGAKD---AGF----KVQAFAF---DGYW--EDI--GT--VEAFYNANLAL--TDPEKAQFSFYDKDAPIYTMSRFL
195   VKFLEK--DYPEDNDFGGE--IIPRAAA---DGA----KVQAYLF---NDYW--EDI--GT--MKSFFEANLNL--AKDP-PNFEFYNAEAPIYTSPRFL
196   RECLLE--NFKEADDFGGE--IIPMAAQ---MGL----KVQAFLY---EGYW--EDI--GT--VDAFFHANLSC--NDPN-PAFNFHEMNAPIYTQSRFL
197   RELLLN--RMPGANDFGNE--VIPGAKD---AGY----KVQAYAF---KGYW--EDI--GT--VEAFYNANLAL--ADPSKAQFSFYDKDAPIYTMSRFL
198   CKLLNE--TYAKANDFGGE--IIPEAAK---N-H----NVVAYPF---YGYW--EDI--GT--IKSFFEENLKL--CRHP-ATFEFYDPQSPIYTSPRVL
199   VKFLDD--DYPEDNDFGGE--IIPKASA---DGA----RVQAYLF---NDYW--EDI--GT--MKSFFEANLAL--AKDP-PNFEFYNAEAPIYTSPRFL
200   EQVLQD--DFPEANDFGGE--IIPMAAQ---KGM----KVVAHLY---DGYW--EDI--GT--VDAFFHANLEC--NDPN-PKFSFYDRNAPIYTQSRFL
201   LKLLRW--RYPTANDFGSE--ILPASAK---E-Y----NVQAYLF---NDYW--EDI--GT--IKSFYEANLAL--TCQP-PKFRFYDAAKPIYTSPRYL
203   SRLLRD--EFPNCNDFGSE--VIPGATQ---LGM----KVQAYLY---DGYW--EDI--GT--IEAFYHANLGF--TKKPVPNFSFYDRSAPIYTQARFL
204   SRLLRD--EFPNCNDFGSE--VIPGATQ---LGM----KVQAYLY---DGYW--EDI--GT--IEAFYHANLGF--TKKPVPNFSFYDRSAPIYTQARFL
206   LKLLRW--RYPTSNDFGSE--IIPAAAS---E-Y----NVQAYLF---NDYW--EDI--GT--IKSFFDANLAL--TAQP-PKFSFYDASNPIFTSPRFL
207   LKLLRW--RYPTSNDFGSE--IIPAAAN---E-Y----NVQAYLF---NDYW--EDI--GT--IKSFFDANLAL--TAQP-PKFSFYDASNPIFTSPRFL
208   LKLLRW--RYPTANDFGSE--ILPASAK---E-Y----NVQAYLF---NDYW--EDI--GT--IKSFYEANLAL--TCQP-PKFRFYDAAKPIYTSPRYL
209   LKLLRW--RYPTANDFGSE--ILPASAK---E-Y----NVQAYLF---NDYW--EDI--GT--IKSFYEANLAL--TCQP-PKFRFYDAAKPIYTSPRYL

               410       420       430       440       450       460       470       480       490       500
      ....|....|....|....|....|....|....|....|....|....|....|....|....|....|....|....|....|....|....|....|
1     PP--SKMLDADVTDSVIG--EG--CVIK--N---CKIHHSVVG--LR--SCI--SEG--AIIEDSLLM--GADYYET--DADRKLLAAK--GSVPI--GI
2     PP--SKMLDADVTDSVIG--EG--CVIK--N---CKIHHSVVG--LR--SCI--SEG--AIIEDSLLM--GADYYET--DADRKLLAAK--GSVPI--GI
3     PP--SKMLDADVTDSVIG--EG--CVIK--N---CKIHHSVVG--LR--SCI--SEG--AIIEDSLLM--GADYYET--DVDRKLLAAK--GSVPI--GI
4     PP--SKMLDADVTDSVIG--EG--CVIK--N---CKIHHSVVG--LR--SCI--SEG--AIIEDSLLM--GADYYET--DADRKLLAAK--GSVPI--GI
5     PP--SKMLDADVTDSVIG--EG--CVIK--N---CKIHHSVVG--LR--SCI--SEG--AIIEDSLLM--GADYYET--ATEKSLLSAK--GSVPI--GI
6     PP--SKMLDADVTDSVIG--EG--CVIK--N---CKIHHSVVG--LR--SCI--SEG--AIIEDSLLM--GADYYET--ASEKSLLSAK--GSVPI--GI
7     PP--SKMLDADITDSVIG--EG--CVIK--N---CKIFHSVVG--LR--SCI--SEG--AIIEDTLLM--GADYYET--EADKSFLAAK--GSVPI--GI
8     PP--SKMLDADITDSVIG--EG--CVIK--N---CKIHHSVVG--LR--SCI--SEG--AIIEDTLLM--GADYYET--DADRRFLAAK--GSVPI--GI
9     PP--SKMLDADVTDSVIG--EG--CVIK--N---CKIHHSVVG--LR--SCI--SEG--AIIEDTLLM--GADYYET--EADKRFLAAK--GSVPI--GI
10    PP--SKMLDADVTDSVIG--EG--CVIK--N---CKIHHSVVG--LR--SCI--SEG--AIIEDTLLM--GADYYET--EADKRFLAAK--GSVPI--GI
11    PP--SKMLDADVTDSVIG--EG--CVIK--N---CKIHHSVIG--LR--SCI--SEG--AIIEDTLLM--GADYYET--DADRRFLAAK--GSVPI--GI
12    PP--SKMLDADVTDSVIG--EG--CVIK--N---CKIHHSVVG--LR--SCI--SEG--AIIEDTLLM--GADYYET--DADRRFLAAK--GSVPI--GI
13    PP--SKMLDADVTDSVIG--EG--CVIK--N---CKIHHSVVG--LR--SCI--SEG--AIIEDTLLM--GADYYET--DADRRFLMAK--GSVPI--GI
14    PP--SKMLDADVTDSVIG--EG--CVIK--N---CKIHHSVIG--LR--SCI--SEG--AIIEDTLLM--GADYYET--DADRTLLAAK--GRVPI--GI
15    PP--SKMLDADITDSVIG--EG--CVIK--N---CKIHHSVIG--LR--SCI--SEG--AIIEDTLLM--GADYYET--DADRKFLAAK--GSVPI--GI
16    PP--SKMLDADITDSVIG--EG--CVIK--N---CKIHHSVVG--LR--SCI--SEG--AIIEDTLLM--GADYYET--DADRRFLAAK--GGVPI--GI
17    PP--SKMLDADITDSVIG--EG--CVIK--N---CKIFHSVVG--LR--SCI--SEG--AIIEDTLLM--GADYYET--EADKRFLAAK--GSVPI--GI
18    PP--SKMLDADITDSVIG--EG--CVIK--N---CKIHHSVVG--LR--SCI--SEG--AIIEDTLLM--GADYYET--DADRRFLAAK--GSVPI--GI
19    PP--SKMLDADITDSVIG--EG--CVIK--N---CKIFHSVIG--LR--SCI--SEG--AIIEDTLLM--GADYYET--DADKRFLAAK--GSVPI--GI
20    PP--SKMLDADITDSVIG--EG--CVIK--N---CKIHHSVVG--IR--TCI--SEG--AIIEDTLLM--GADYYET--DADRRLLAAK--GSVPI--GI
21    PP--SKMLDADITDSVIG--EG--CVIK--N---CKIHHSVVG--IR--TCI--SEG--AIIEDTLLM--GADYYET--DADRRLLAAK--GSVPI--GI
22    PP--SKMLDADVTDSVIG--EF--CVIK--N---CKIHHSVVG--LR--SCI--SEG--AIIEDTLLM--GADYYET--DADRRFLAAK--GSVPI--GI
23    PP--SKMLDADVTDSVIG--EG--CVIK--N---CKIHHSVVG--LR--SCI--SEG--AIIEDTLLM--GADYYET--DADRRFLAAK--GSVPI--GI
24    PP--SKMLDADITDSVIG--EG--CVIK--N---CKIHHSVIG--LR--SCI--SEG--AVIEDTLLM--GADYYET--DVDRRLMAKK--GSVPI--GI
25    PP--SKMLDADVTDSVIG--EG--CVIK--N---CKIHHSVVG--LR--SCI--SEG--AIIEDTLLM--GADYYET--DADRRLLAAK--GSVPI--GI
26    PP--SKMLDADVTDSVIG--EG--CVIK--N---CKIHHSVVG--VR--SCI--SEG--AIIEDSLLM--GADYYET--DADRRLLAAK--GSIPI--GI
27    PP--SKMLNADVTDSVIG--EG--CVIK--N---CKIHHSVIG--LR--SCI--SEG--AIIEDTLLM--GADYYET--DSDRRILAAK--GGIPI--GI
28    PP--SKMLDADVTDSVIG--EG--CVIK--N---CKIHHSVVG--LR--SCI--AEG--AIIEDTLLM--GADYYET--DADRRFLAAK--GGVPI--GI
29    PP--SKMLDADVTDSVIG--EG--CVIK--N---CKIHHSVVG--LR--SCI--SEG--AIIEDTLLM--GADYYET--DADKRFLAAK--GSVPI--GI
30    PP--SKMLDADITDSVIG--EG--CVIK--N---CKIHHSVVG--LR--SCI--SEG--AIIEDTLLM--GADYYET--DADRRFLAAK--GGVPI--GI
31    PP--SKMLDADITDSVIG--EG--CVIK--N---CKIFHSVVG--LR--SCI--SEG--AIIEDTLLM--GADYYET--EADKRFLAAK--GSVPI--GI
32    PP--SKVLDADVTDSVIG--EG--CVIK--H---CTINHSVVG--LR--SCI--SEG--AVIEDSLLM--GADYYET--ENDKNVLSET--GGIPI--GI
```

```
33   PP--SKVLDADVTDSVIG--EG--CVIK--N---CKINHSVVG--LR--SCI--SEG--AIIEDSLLM--GADYYET--EADKKLLAEK--GGIPI--GI
34   PP--SKVLDADVTDSVIG--EG--CVIK--N---CKINHSVVG--LR--SCI--SEG--AIIEDSLLM--GADYYET--EADKKLLAEK--GGIPI--GI
35   PP--SKVLDADVTDSVIG--EG--CVIK--N---CKINHSVVG--LR--SCI--SEG--AIIEDSLLM--GADYYET--EADKKLLAEK--GGIPI--GI
36   PP--SKVLDADVTDSVIG--EG--CVIK--N---CKIHHSVVG--LR--SCI--SEG--AIIEDTLLM--GADYYET--EADKKLLAEN--GGIPI--GI
37   PP--SKVLDADVTDSVIG--EG--CVIK--N---CKIHHSVVG--LR--SCI--SEG--AIIEDSLLM--GADYYET--EADKKLLGEK--GGIPI--GI
38   PP--SKVLDADVTDSVIG--EG--CVIR--H---CTINHSVVG--LR--SCI--SEG--AVIEDSLLM--GADYYET--ETDKKALSET--GGIPI--GI
39   PP--SKVLDADVTDSVIG--EG--CVIK--N---CKIHHSVVG--LR--SCI--SEG--AIIEDSLLM--GADYYET--EADKKLLGEK--GGIPI--GI
41   PP--SKVLDADVTDSVIG--EG--CVIK--N---CKIHHSVVG--LR--SCI--SEG--AIIEDTLLM--GADYYET--EADKQLLAEK--GGIPI--GI
42   PP--SKVLDADVTDSVIG--EG--CVIK--N---CKIHHSVVG--LR--SCI--SEG--AIIEDTLLM--GADYYET--EADKQLLAEK--GGIPI--GI
43   PP--SKVLDADVTDSVIG--EG--CVIK--H---CTINHSVVG--LR--SCI--SEG--AVIEDSLLM--GADYYET--EDDKKVLSEN--GGIPI--GI
44   PP--SRVLDADVTDSVIG--EG--CVIN--H---CKINHSVVG--LR--SCI--SEG--AVIEDSLLM--GADYYET--ENDKKVLSET--GGIPI--GI
45   PP--SKVLDADVTDSVIG--EG--CVIK--N---CKIHHSVVG--LR--SCI--SEG--AIIEDTLLM--GADYYET--EADKKLLAEK--GGIPI--GI
46   PP--SKVLDADVTDSVIG--EG--CVIK--N---CKIHHSVVG--LR--SCI--SEG--AIIEDTLLM--GADYYET--EADKKLLAEK--GGIPI--GI
47   PP--SKVLNADVTDSVIG--EG--CVIN--H---CTINHSVVG--LR--SCI--SEG--AVIEDSLLM--GADYYET--ENDKKVLSES--GGIPI--GI
48   PP--SKVLDADVTDSVIG--EG--CVIK--N---CKIHHSVVG--LR--SCI--SEG--AIIEDTLLM--GADYYET--EADKKLLAEK--GGIPI--GI
49   PP--TKIDNCKIKDAIIS--HG--CFLR--D---CSVEHSIVG--ER--SRL--DCG--VELKDTFMM--GADYYQT--ESEIASLLAE--GKVPI--GI
50   PP--TKVDKSRIVDAIIS--HG--CFLR--E---CNIQHSIVG--VR--SRL--DYG--VEFKDTMMM--GADYYQT--ECEIASLLAE--GKVPI--GV
51   PP--SAIDNSKIVDSIVS--HG--IFLT--N---CFVEHSVVG--IR--SRI--GTN--VHLKDTVML--GADYYET--DAEIRSQLAE--GKVPL--GI
52   PP--SKIDNSKLIDSIIS--HG--SFLT--N---CLIEHSIVG--IR--SRV--GSN--VQLKDTVML--GADYYET--EAEVAALLAE--GNVPI--GI
53   PP--TKVDKCRILDSIVS--HG--CFLR--E---CSVQHSIVG--IR--SRL--ESG--VELQDTMMM--GADFYQT--EAEIASLLAE--GKVPV--GV
54   PP--TKTEKCRIVNSVIS--HG--CFLG--E---CSIQRSIIG--ER--SRL--DYG--VELQDTLML--GADSYQT--ESEIASLLAE--GNVPI--GI
55   PP--TKAEKCRMVDSIIS--HG--CFLR--E---CSVQRSIIG--ER--SRL--DYG--VELQDTLML--GADYYQT--ESEIASLLAE--GKVPI--GI
56   PP--TKIDNCKIKDAIIS--HG--CFLR--D---CTVEHSIVG--ER--SRL--DCG--VELKDTFMM--GADYYQT--ESEIASLLAE--GKVPI--GI
57   PP--TKVDKSRIVDAIIS--HG--GFLR--E---CNIQHSIVG--VR--SRL--DYG--VEFKDTMMM--GADYYQT--ESEIASLLAE--GKVPI--GV
58   PP--SAIDNSKIVDSIVS--HG--SFLT--N---CFVEHSVVG--IR--SRI--GTN--VHLKDTVML--GADYYET--DAEIASQLAE--GKVPL--GI
59   PP--TKIDNCKIKDAIIS--HG--CFLR--D---CTVEHSIVG--ER--SRL--DCG--VELKDTFMM--GADYYQT--ESEIASLLAE--GKVPI--GI
60   PP--TKVDKSRIVDAIIS--HG--CFLR--E---CNIQHSIVG--VR--SRL--DYG--VEFKDTMMM--GADYYQT--ESEIASLLAE--GKVPI--GV
61   PP--SKIDNSKLVDSIIS--HG--SFLT--N---CLIEHSIVG--IR--SRV--GSN--VQLKDTVML--GADYYET--EAEVASLLAE--GKVPI--GI
62   PP--TKVDKCRILDSIVS--HG--CFLR--E---CSVQHSIVG--IR--SRI--ESG--VELQDTMMM--GADFYQT--EAEIASLLAE--GKVPV--GV
63   PP--TKTEKCRIVNSIIS--HG--CFLG--E---CSIQRSIIG--ER--SRL--DYG--VELQDTLML--GADSYQT--ESEIASLLAE--GNVPI--GI
64   PP--TKAEKCRMVDSIIS--HG--CFLR--E---CSIQRSIIG--ER--SRL--DYG--VELQDTLML--GADYYQT--ESEIASLLAE--GKVPI--GI
66   PP--TKMEKCEVINSLIS--DG--CFLK--E---CTVEHSIVG--IR--SRL--DSG--VQLKDTMIM--GADYYQT--EAEIASLLAA--GNVPI--GI
67   PP--SKIDNSKIVDSIIS--HG--SFLN--N---SFIEHSVVG--IR--SRI--NSN--VHLKDTVML--GADYYET--DAEVVALLAE--GRVPI--GI
68   PP--TKAIKCKIMDAIIS--HG--CFLS--E---SRVQHSIVG--VR--SRL--ESG--SELQDTMMM--GADYYQT--DSEIATLLEE--GKVPI--GV
69   PP--TKAIKCKIVDAIIS--HG--CFLS--E---CRVQHSIVG--VR--SRL--ESG--SELQDTMMM--GADYYQT--DSEIATLLKE--GKVPI--GV
70   PP--TKIDKCRIVDAIIS--HG--CFLR--E---CTVQHSIVG--ER--SRL--DYG--VELQDTVMM--GADYYQT--ESEIASLLAE--GKVPI--GI
71   PP--TKIDKCRIVDAIIS--HG--CFLR--E---CTVQHSIVG--ER--SRL--DYG--VELQDTVMM--GADYYQT--ESEIASLLAE--GKVPI--GI
72   PP--SKIDNSKIVDSIIS--HG--SFLN--N---SFIEHSVVG--IR--SRI--NSN--IHLKDTVML--GADYYET--DAEVAALLAE--GRVPI--GI
73   PP--TKVEKCKIVDAIIS--HG--CFLR--E---CSVQHSIVG--VR--SRL--ESG--VELQDTMMM--GADYYQT--EYEIASLVAE--GKVPI--GV
74   PP--TKIDKCRIVDAIIS--HG--CFLR--E---CTVQHSIVG--ER--SRL--DYG--VEVLDTVMM--GADYYQT--ESEIASLLAE--GKVPI--GI
75   PP--TKVEKCKIVDAIIS--HG--CFLR--E---CSIQHSIVG--VR--SRL--ESG--VELQDTMMM--GADYYQT--EYEIASLLAE--GKVPI--GV
76   PP--TKIDKCQIVDAIIS--HG--CFLR--E---CTVQHSIVG--ER--SRL--DYG--VELLDTVMM--GADYYQT--ESEIASLLAE--GKVPI--GI
77   PP--TKMEKCEVINSLIS--DG--CFLK--E---CTVEHSIVG--IR--SRL--DSG--VQLKDTMIM--GADYYQT--EAEIASLLAA--GNVPI--GI
80   PP--TKIDKCRIVDAIIS--HG--CFLR--E---CSVQHSIVG--ER--SRL--DYG--VELQDTVMM--GADYYQT--ESEIASELAE--GKVPI--GI
81   PP--TKIEKCRVKDSIVS--HG--CFLR--E---CSVERSIVG--VR--SRL--EYG--VELKDTMMI--GADYYQT--EAEIAASLAE--GRVPV--GV
82   PP--TKFDKCRIVNAIIS--HG--CFLR--E---CTVQHSVVG--ER--SRL--DYG--VELKDTVML--GADCYQT--EVEIASLLAE--GEVPI--GV
84   PP--SKIDSSKIVDSIIS--HG--SFLN--N---CFIEHSVIG--IR--SRI--NSN--AHLQDTVML--GADFYET--EAEVASVVAE--GSVPV--GI
87   PP--SKIENCKIVDSIIS--HG--SFLT--N---SFIEHSVVG--IR--SRI--NSN--VHLKDTVML--GADFYET--DDEVAALLAE--GRVPI--GI
88   PP--TKIERCQVKDSIIS--HG--CFLR--E---CSVEHSIVG--VR--SRL--EYG--VELKDTMMM--GADYYQT--EAEVAASLAG--GKVPI--GV
89   PP--TKIDKCRIVDAIIS--HG--CFLR--E---CTVRHSVVG--ER--SRL--DYG--VELKDTVML--GADYYQT--ETEIASLLAE--GKVPI--GV
90   PP--TKVDQCRIVDAIIS--HG--CFLQ--E---CSIKHSIVG--VR--SRL--ESA--VELMDTMMM--GADYYQT--ESEIASLQAE--GKVPI--GV
91   PP--TKVEECRILDAIIS--HG--CFLR--E---CSVQRSIVG--VR--SRL--EYG--VELKDTMMM--GADYYQT--ESEIASLLAE--GKVPI--GV
92   PP--TKIEKCRVMDSIIS--HG--CFLR--E---CSVEHSIVG--IR--SRL--DYG--VEMKDTMMM--GADYYQT--EEEIAAFLAE--GKVPI--GV
94   PP--SKIDDSKIVDSIIS--HG--SFLN--N---CFIEHSVVG--IR--SRV--NSN--VHLKDTVML--GADYYET--DSEVASLLAE--GRVPI--GI
95   PP--TKIEQCQVVDAIIS--HG--CFLR--E---CSVKHSIVG--ER--SRL--DYG--VELKDTLMM--GADFYQT--ESEIASLLAE--GNVPI--GI
96   PP--TKVDRCRIVDAIIS--HG--CFLR--E---CSVQHSIVG--VR--SRL--ESG--VELTDTMMM--GADYYQT--ESEIASLLAE--GKVPI--GV
97   PP--TKIDKCRIVDAIIS--HG--CFLR--E---CTVQHSVVG--ER--SRL--DYG--VELKDTVML--GADHYQT--EAEIASLLAE--GKVPI--GV
98   PP--TKVDKCRIVDAIIS--HG--CFLR--E---CSVQHSIVG--VR--SRL--ESG--VELTDTMMM--GADYYQT--ESEIASVLAE--GKVPI--GV
99   PP--SMVNNSKITDSIIS--HG--CFLD--N---CRIEHSVVG--VR--SRI--GSN--VHLKDTVML--GADYYET--AVERGELLAE--GKVPI--GI
100  PP--TQLDKCKMKYAFIS--DG--CLLR--E---CNIEHSVIG--VC--SRV--SSG--CELKDSVMM--GADTYET--EEEASKLLLA--GKVPV--GI
101  PP--TKSDKCRIKDAIIS--HG--CFLR--E---CAIEHSIVG--VP--SRL--NSG--CELKNTMMM--GADLYET--EDEISRLLAE--GKVPI--GV
102  PP--TKVENCKVLNSIVS--HG--CFLT--E---CSVEHSVIG--IR--SRL--EPG--VQLKDTMMM--GADYYQT--EAERLSELSV--GKVPV--GV
103  PP--TKSDKCRIKEAIIS--HG--CFLR--E---CTIEHSIVG--VR--SRL--NSG--CELKNAMMM--GADLYET--EDEISRLLSE--GKVPI--GV
104  PP--TKVEDCKVLNSIVS--HG--CFLT--E---CSVEHSVIG--IR--SRL--QPG--VQLKDTMMM--GADYYQT--EAERFSELSD--GKVPV--GV
105  PP--SMISSSKITDSIIS--HG--CFLD--N---CRVEHSVVG--VR--SRV--GSN--VHLKDTVML--GADFYET--DVERSDQLAE--GKVPI--GI
106  PP--SMINNSKITDSIIS--HG--CFLD--S---CRIEHSVVG--IR--SRI--GSN--VHLKDTVML--GADFYET--DLERGELLAE--GKVPI--GI
107  PP--TKSDKCRIKDAIIS--HG--CFLR--E---CTIEHSIVG--VR--SRL--NSA--CELKNTMMM--GADLYET--EDEISRLLSE--GKVPI--GV
108  PP--TKVENCKVLNSIVS--HG--CFLT--E---CSVDRSVIG--VR--SRL--EPG--VQLKDTMMM--GADYYQT--EAERFSELSD--GKVPV--GV
109  PP--ARLEKCKIKDAIIS--DG--CSFS--E---CTIEHSVIG--IS--SRV--SIG--CELKDTMMM--GADQYET--EEETSKLLFE--GKVPI--GI
110  PP--TKSDKCRIKDAIIS--HG--CFLR--E---CAIEHSIVG--VR--SRL--NSG--CELKNTMMM--GADLYET--EDEISRLLSE--GKVPI--GV
111  PP--TQLDKCKIKDASIS--DG--CLLR--E---CSIEHSVIG--VC--SRV--SYG--CELKDCVMM--GADIYET--EEEASKLLLA--GEVPV--GI
112  PP--SMVNNSKITDSIIS--HG--CFLD--N---CRIEHSVVG--VR--SRI--GSN--VHLKDTVML--GADYYET--DAERRELLAE--GNVPI--GI
113  PP--TKSDKCRIKEAIIS--HG--CFLR--E---CKIEHSIIG--VR--SRL--NSG--SELKNAMMM--GADSYET--EDEISRLMSE--GKVPI--GV
114  PP--SMISGSKITDSIIS--HG--CFLD--K---CRVEHSVVG--IR--SRI--GSN--VHLKDTVML--GADFYET--DAERGDQLAE--GKVPI--GI
115  PP--TKSDKCRIKEAIIS--HG--CFLR--E---CKIEHSIIG--VR--SRL--NSG--SELKNAMMM--GADSYET--EDEISRLMSE--GKVPI--GV
116  PP--TAINNSKIVDSIIS--HG--SLLS--N---CLIEHSVVG--IR--SRI--NDN--VHLKDTVML--GADLYET--DAEIAALLAE--GRVPV--GI
117  PP--TKSDKCRIKEAIIS--HG--CFLR--E---CKIEHSIIG--VR--SRL--NSG--SELKNAMMM--GADSYET--EDEISRLMSE--GKVPI--GV
```

```
118  PP--SMISGSKITDSIIS--HG--CFLD--K---CRVEHSVVG--IR--SRI--GSN--VHLKDTVML--GADFYET--DMERGDQLAE--GKVPI--GI
119  PP--TKSDKCRIKEAIIS--HG--CFLR--E---CKIEHSIIG--VR--SRL--NSG--SELKNAMMM--GADSYET--EDEISRLMSE--GKVPI--GV
120  PP--TKSDKCRIKEAIIL--HG--CFLR--E---CKIEHTAF-------SRL--NSG--SELKNAMMM--GADSYET--EDEMSRLMSE--GKVPI--GV
121  PP--SKLVDAQITESIIG--EG--TILK--S---CSIHHCVLG--VR--SRV--END--VVLQDSLLM--GADFFES--STERSVLRER--GGIPV--GV
122  PP--SKMLDCQITESIIA--EG--CILK--E---CRIDHSVLG--LR--SRV--ESG--SLVEDTMLM--GSDFYQP--FAERQYGLEK--GSVPI--GI
123  PP--SKLLSCHVTESIIG--EG--CILK--D---CRIQHSVLG--VR--SRI--EAG--CVIEESLLM--GADFYQP--FVERQCNLEK--GDIPV--GI
124  PP--TKVLNSNITESMIS--EG--CMIK--D---CRIHNSVLG--IR--SRI--ETD--CVVEDSLLM--GADYYES--LDDRQSLLDQ--GKIPI--GI
125  PP--TKQQKCQVIESMIS--EG--CILK--E---CYIENSVIG--IR--SRI--DSG--CTIKNVLLM--GADYYQS--DFENEGDCSL--ENIPI--GI
126  PP--SKMLDAQVTQSIIG--EG--SMLK--A---CSIHHCVLG--VR--TRV--EDE--AVLQDTLVM--GSDFFES--SEERAVLRER--GGIPL--GV
127  PP--SKLLDSQVTQSIIG--EG--SILK--A---CSIHHCVLG--VR--SRV--EED--AVLQDTLVM--GNDFFES--SAERNALRHR--GGTPV--GV
128  PP--SKLLDAQVTQSIIG--EG--SLLQ--D---CSIHHCVLG--VR--SRI--ESE--VVLQDTLVM--GADFFES--SEERAVLRER--GGIPV--GV
129  PP--SKILDCQITESIIS--EG--SILK--Q---CRIGHSVLG--LR--SRI--EAG--CVIEDTLVM--GSDYYEP--FAERQSNIQQ--GKIPM--GI
130  PP--SKVGDSQIIDSIIG--EG--SIIK--S---CSVNHCVLG--IR--SRI--ENS--VVVQDSLVM--GSDFYES--TQEREELRRN--GGIPL--GV
131  PP--SKLVDAQIVNSIIG--EG--SILK--S---CSINHCVLG--VR--SRV--ETD--VVLQDTLVM--GADFFES--NDEREAIRQK--GGIPV--GV
132  PP--SKLVDSQITDSIIG--EG--SILK--S---CSIHHSVLG--VR--SRV--EDD--VVLQDSLLM--GSDFFES--SSERAVLKER--GGIPL--GV
133  PP--SKLIDCQIAESIIT--EG--CIIK--Q---ARIFHSVLG--LR--SRI--ESG--VRIEDSLLM--GADFYET--PIQREESLRR--GLPPV--GI
134  PP--SKLVDAQITDSIVC--EG--TILK--S---CSILHCVLG--VR--SRI--ESD--SVIEDTLVM--GSDFFES--LEERIELRKG--GGTPL--GV
135  PP--SKLVDAQITDSIVG--EG--SILK--S---CSIHHCVLG--VR--SRI--ESD--VVLEDSLVM--GSDFYES--AEERIALRKG--GGIPL--GV
136  PP--SKILSSTITESIIS--EG--CILK--E---CQVHRSVLG--VR--SRV--ESG--CVIDHSLLM--GADYYQD--SAQRSQLRLQ--HKIPI--GI
137  PP--TKALNCTITESMVS--EG--CILK--D---CRIHNSILG--IR--TRI--EAN--CTIEDTMLM--GADYYES--PSLRESKAQE--GKIPM--GI
138  PP--TKLLDCQITESIIG--EG--CILK--N---CRIQHSVLG--VR--SRI--ESG--CVIEESLLM--GADFYQA--SVERQCSLIE--NDIPV--GI
139  PP--TKLVDAQITESIIG--EG--SILK--S---CSIHHCVLG--VR--SRV--ESD--VVLQDSLVM--GSDFYES--SEERTLLRQG--GGIPL--GV
140  PP--NKILDSQIVNSMIA--DG--CIIK--N---AQIRNSIIG--IR--SRL--EAN--TIIENTLVM--GADYYES--AEERQARLEE--GIPPV--GI
141  PP--NKILDSQIVNSMIA--DG--CIIK--N---AQIRNSIIG--IR--SRL--EAN--TIVENTLVM--GADYYES--AEERQAKLEA--GIPPV--GI
142  PP--TKLVDAQITESIIG--EG--SILK--S---CSIHHCVLG--VR--SRV--ESD--VVLQDSLVM--GSDFYES--SEERTLLRQG--GGIPL--GV
143  PP--SKLVDAQITNSIVG--EG--SILK--S---CSIHHCVLG--VR--SRI--ETD--VVLQDTLVM--GADFFES--SDERAVLRER--GGIPV--GV
144  PP--TKLLDCHVTESIIG--EG--CILK--N---CRIQHSVLG--VR--SRI--ETG--CMIEESLLM--GADFYQA--SVERQCSIDK--GDIPV--GI
145  PP--SKLVDAQITDSIVG--EG--SILK--A---CSIHHCVLG--VR--SRI--ESD--VVLQDTLVM--GSDFYES--GEERIALRSG--GGIPL--GV
146  PP--SKLVDAQITDSIVC--EG--TILK--S---CSILHCVLG--VR--SRI--ESD--SVLEDTLVM--GADFFES--PEERFELRKG--GGTPL--GV
147  PP--SKIVDTQITDSIVS--EG--SILK--S---CSIHHCVLG--VR--SRI--ESD--VVLNETLVM--GSDFYES--YEERIALRNG--GGIPL--GV
148  PP--SKLVDAQITDSIVC--EG--TILK--S---CSILHCVLG--VR--SRI--ESD--SVLEDTLVM--GADFFES--PEERIELRKG--GGTPL--GV
149  PP--SKLVDAQITDSIVC--EG--TILK--S---CSILHCVLG--VR--SRI--ESD--SVIEDALVM--GADFFES--QEERVELRKG--GGTPL--GV
150  PP--SKFVDSQITDSIIS--EG--SIIK--A---CSIHHSVLG--VR--SRV--ENN--VVLQDSLLM--GADFFES--QGERETLRAR--GGIPV--GV
151  PP--TKVLNCTITESMIS--EG--CILK--D---CRIHHSVLG--IR--SRV--ESD--CTIEDSMLM--GADYYES--STKRKAVLEA--GKVPQ--GI
152  PP--SKLVDAQITDSIVC--EG--TILK--S---CSILHCVLG--VR--SRI--ESD--SILEDTLVM--GADFFES--PEERIELRKG--GGTPL--GV
153  PP--TKMLNSTVTESMIG--EG--CMIK--Q---CRIHHSVLG--IR--SRI--ESD--CTIEDTLVM--GNDFYES--SSERDTLKAR--GEIAA--GI
154  PP--TKALNCTITESMVS--EG--CILK--D---CRIHNSILG--IR--TRI--EAN--CTIEDTMLM--GADYYES--PSLRESKAQE--GKIPM--GI
155  PP--TKLLDCQVTQSIIG--EG--CILK--Q---CTVQNSVLG--IR--SRI--EAD--CVIQDALLM--GADFYET--SELRHQNRAN--GKVPM--GI
156  PP--SKLLDCQVTESIIG--EG--CILK--N---CQIQHSVLG--VR--SRI--ESG--CVIDNALLM--GADFYQP--FAERDHKIKN--NSVPL--GI
157  PP--TKLLDCQVTQSIIG--EG--CILK--Q---CTVQNSVLG--IR--SRI--EAD--CVIQDALLM--GADFYET--SELRHQNRAN--GKVPM--GI
158  PP--SKLVDAQITNSIVG--EG--SILK--S---CSIHHCVLG--VR--SRI--ESD--CVLQDTLVM--GADFFES--PDERAVLKER--GGIPL--GV
159  PP--SKLVDAQIVNSIIG--EG--SILK--S---CSINHCVLG--VR--SRV--ETD--VVLQDTLVM--GADFFES--NEERETIRQQ--GGIPV--GV
160  PP--SKILDCRVTESIVG--EG--CIVK--K---SQIHHSVLG--VR--SYV--DDH--CTLDNVLWL--GSDYYQS--LSERQADLDQ--GRVPL--GI
161  PP--TKQLDCHVTESMIA--EG--CIIK--N---CQINRSVLG--VR--SRV--ESG--CTLDNALVM--GADYYQP--FAERASGMGD--TSIPI--GI
162  PP--SKLLDCEITESIVG--EG--CILK--K---CRIDHCVLG--VR--SRI--EAN--CIIQDSLLM--GSDFYES--PTERRYGLKK--GSVPL--GI
163  PP--TKLLDCHVTESIIG--EG--CILK--N---CRIQHSVLG--VR--SRI--ETG--CVIEESLLM--GADFYQA--SVERQCSIDK--GDIPV--GI
164  PP--TKQLKCEVIQSMIS--EG--CVLK--D---CYIENSVIG--IR--SRI--DSG--CTIKNVLLM--GADYYQS--DFENEGDCSL--ENIPI--GI
165  PP--TKVLNCNITESMIS--EG--CMIK--D---CRIHNSVLG--IR--SRI--ETD--CVVEDSLLM--GADYYES--LETRQSLLDQ--GKIPV--GI
166  PP--TKVLNADVTESMIS--EG--CIIK--N---CRIHHSVLG--IR--TRV--EAD--CTIEDTMIM--GADYYQP--YEKRQDCLRR--GKPPI--GI
167  PP--SKLVDAQITDSIVC--EG--TILK--S---CSILHCVLG--VR--TRI--ESD--SVLEDTLVM--GADFFES--PEERIELRRG--GGTPL--GV
168  PP--SKIVDTQITDSIVS--EG--SILK--S---CSIHHCVLG--VR--SRI--ESD--VVLNETLVM--GSDFYES--YEERIALRNG--GGIPL--GV
169  PP--SKLQDAQVTDSIVG--EG--SILK--A---CSIHHCVLG--VR--SRI--EDE--VALQDTLVM--GNDFYES--GEERAILRER--GGIPM--GV
170  PP--SKLVDSQITDSIIG--EG--SILK--S---CSIHHSVLG--VR--SRV--EDE--VVLQDSLLM--GSDFFES--SSERAVLRER--GGIPL--GV
171  PP--TKMLNCTVTESMIS--EG--CILK--E---CRIHHSILG--IR--SRV--GKD--CTIEDTMLM--GADFYES--FPERESLIGN--AKIPV--GI
172  PP--SKLVDAQITDSIVC--EG--TILK--S---CSILHCVLG--VR--TRI--ESD--SVLEDTLVM--GADFFES--PEERIELRRG--GGTPL--GV
173  PP--SKLQDAQVTESIIG--EG--SLLK--A---CSIHHCVLG--VR--SRV--EDR--VVLQDTLVM--GSDYFES--SEERATLRQR--GGIPL--GV
174  PP--TKMVDCTITESMIS--EG--CILK--E---CRIHHSVLG--IR--ARV--EAG--CTIEDSLLM--GLDFYES--SAQRKAASQA--GKVPQ--GI
175  PP--SKLLNCDITESMIG--EG--CILK--N---CRIQHSVLG--VR--SRI--ESG--CVIEESLLM--GADYYQP--SVERQCSLEQ--GDIPV--GI
176  PP--SKLLDCRVTESIIG--EG--CILK--D---CRINNSVLG--LR--SRV--EAG--TVIEDTLIM--GADYYQS--LTERLSAQEQ--GQVTL--GI
177  PP--SKLLDCHVTQSIIG--EG--CILK--N---CRIENSVLG--VR--SRI--ESG--CIIQDSMIM--GADMYQP--FAERQSDCDH--RSVPL--GI
178  PP--TKVLNCNITESMIS--EG--CMIK--D---CRIHNSVLG--IR--SRI--ETD--CVVEDSLLM--GADYYES--LETRQSLLDQ--GKIPV--GI
179  PP--TKQLKCEVIQSMIS--EG--CVLK--D---CYIENSVIG--IR--SRI--DSG--CTIKNVLLM--GADYYQS--DFENEGDCSL--ENIPI--GI
180  PP--SKFVDSQITDSIIS--EG--SIIK--A---CSIHHSVLG--VR--SRV--ENN--VVLQDSLLM--GADFFES--QSERETLRAR--GGIPV--GV
181  PP--TKVFDSHITKSMIS--EG--CIIK--K---CRIHNSILG--IR--SRI--EMN--CHIEDTMIM--GADFYES--STVNSSYSSP--KEIPI--GI
182  PP--TKLLDTHVTESIIA--EG--CILK--Q---CRIDHSVLG--VR--SRI--EAG--CTIQDTLVM--GADFYEP--DAERHSSLGT--GGVAL--GI
183  PP--SKLLDCQVTESIIA--EG--CILK--E---CRIDHSVLG--VR--SRI--EAG--CNIEDSLIM--GSDFYEP--FAERQSGSDK--GGVPV--GI
184  PP--SKLLDCDIKESMIG--EG--CILK--N---CRIQHSVLG--VR--SRV--ESG--SIVEESLIM--GSDFYQP--SVERVCNLDK--GDIPL--GI
185  PP--SKLLDCDIKESMIG--EG--CILK--N---CRIQHSVLG--VR--SRV--ESG--SIVEESLIM--GSDFYQP--SVERVCNLDK--GDIPL--GI
186  PP--SKMLDCQITESIIA--EG--CILK--E---CRIDHSVLG--LR--SRV--ESG--SLVEDTMLM--GSDFYQP--FAERQYGLEK--GSVPI--GI
187  PP--TKVLNCNITESMIS--EG--CMIK--D---CRINNSVLG--IR--SRI--ESD--CVVEDSLLM--GADFYES--LDTRQSLLDQ--GKIPV--GI
188  PP--SKLVDAQITNSIVG--EG--SILK--S---CSIHHCVLG--VR--SRI--ESD--CVLQDTLVM--GADFFES--PDERAVLKER--GGIPL--GV
189  PP--AKIEKCHVKDAIIS--HG--CSLA--D---CCVENAIVG--LR--SQV--GKG--CKIERAMII--GADFYES--EDQKAKVIAS--GGVPV--GI
190  PP--SKLLDVQVSRSTIG--DG--CFIK--K---STISNSMIG--LR--TSI--SEG--CVIEDSMIM--GADYYEE--THECEDLP----DCTPI--GI
191  PP--AKVEKCHVKDAIIS--HG--CSLA--D---CSVEDAIIG--LR--SQI--GKG--CTIKHAMII--GADYYET--DEQKMALVEA--GGVPV--GI
192  PP--SKLLDAEVSKCTIG--DG--CFIK--K---SKLTNAMIG--LR--TNI--QED--CVIEDVMIM--GADYYEE--THECEDLP----GCTPI--GI
193  PP--ATVRNCKVTDAIIA--QG--SFVS--D---CTINNAVIG--IR--SII--GQN--CTIQDALVM--GADYYES--DDQRATLLKK--GGVPV--GI
194  PP--SKVMDCDVNMSIIG--DG--CVIK--AG--SKIHNSIIG--IR--SLI--GSD--CIIDSAMMM--GSDYYET--LEECEYVP----GCLPM--GV
```

```
195   PP--AKVERCHVKESIIS--HG--ASLA--D---CQVEESIIG--LR--SVV--NKG--CRIKRAMII--GADFYES--DEKKASLLAS--GEVPV--GI
196   PP--SKVQDCEIERSTIG--DG--CFIT--K---AKLKNVMVG--LR--STV--NAN--CDLEDTLVM--GADYYET--YDEAKTSALP--GGVPI--GI
197   PP--SKVLDADVSMSIIG--DG--CVIK--AG--SKIHNSIIG--IR--SLV--GSD--CIIDSAMMM--GADYYET--LEECEYVP----GCLPM--GV
198   PP--ATVRNCKVSDAIIA--QG--SFVA--D---SSISNAVIG--IR--SII--GSG--CTVQDALIM--GADYYQS--DEQRAALLAA--GDVPV--GI
199   PP--AKIERCHVKDSIIS--HG--AALA--D---CSVEESIVG--LR--SRV--EAG--TKIKRTMII--GADFYES--EEKRKAILAA--GGVPV--GI
200   PP--SKVQDCEIERSTIG--DG--CTIK--Q---AKLKNVMVG--LR--STV--NEG--CDLEDTLVM--GADYYES--LEECDPASLP--GCTPI--GI
201   PP--TKIEKCRVLDSIVS--HG--CFLQ--E---CSVTHSVIG--IR--SRV--EAG--AEIQDTMML--GADFYET--EAEIASMVAE--GKVPV--GV
203   PP--SKLIDADVTDSVIG--EG--CLIK--S---CKIHHSVIG--LR--SWI--AED--ALVEDALLM--GADFYET--DEERDALLLK--GGVPV--GI
204   PP--SKLIDADVTDSVIG--EG--CLIK--S---CKIHHSVIG--LR--SWI--AED--ALVEDALLM--GADFYET--DEERDALLLK--GGVPV--GI
206   PP--TKMEKCRIIDSIVS--HG--CFLK--S---CSVEHSLIG--VR--SRL--ESG--VELKDTIIM--GADSYET--EAEIAALRAQ--GKVPL--GV
207   PP--TKMEKCRIIDSIVS--HG--CFLK--S---CSVEHSLIG--VR--SRL--ESG--VELKDTIIM--GADSYET--EAEIAALRAQ--GKVPL--GV
208   PP--TKIEKCRVLDSIVS--HG--CFLQ--E---CSVTHSVIG--IR--SRV--EAG--AEIQDTMML--GADFYET--EAEIASMVAE--GKVPV--GV
209   PP--TKIEKCRVLDSIVS--HG--CFLQ--E---CSVTHSVIG--IR--SRV--EAG--AEIQDTMML--GADFYET--EAEIASMVAE--GKVPV--GV

               510       520       530       540       550       560       570       580       590
      ....|....|....|....|....|....|....|....|....|....|....|....|....|....|....|....|....|....|....
1     G--KN--CHIKRAIID--KN--ARIG--DN--VKI--INKDNV---QEAARE--TD--GYFIKS--G--IVTVI--KD--ALI---PSGIII--
2     G--KN--CHIKRAIID--KN--ARIG--DN--VEI--INKDNV---QEAARE--TD--GYFIKS--G--IVTVI--KD--ALI---PSGIII--
3     G--KN--CHIKRAIID--KN--ARIG--DN--VKI--INKDDV---QEAARE--TD--GYFIKS--G--IVTVI--KD--ALI---PSGIII--
4     G--KN--CHIKRAIID--KN--ARIG--DN--VKI--INKDNV---QEAARE--TD--GYFIKS--G--IVTVI--KD--ALI---PSGIVI--
5     G--KN--SHIKRAIID--KN--ARIG--DN--VKI--INSDNV---QEAARE--TD--GYFIKS--G--IVTVI--KD--ALI---PTGTVI--
6     G--KN--SHIKRAIID--KN--ARIG--DN--VKI--INSDNV---QEAARE--TD--GYFIKS--G--IVTVI--KD--ALI---PTGTLI--
7     G--RN--SHIKRAIVD--KN--ARIG--EN--VKI--INSDNV---QEAARE--TE--GYFIKS--G--IVTII--KD--ALI---PSGTVI--
8     G--KN--SHIKRAIID--KN--ARIG--DD--VKI--INSDNV---QEAARE--TE--GYFIKS--G--IVTVI--KD--ALI---PSGTVI--
9     G--RN--SHIKRAIID--KN--ARIG--EN--VKI--INSDNV---QEAARE--TD--GYFIKS--G--IVTVI--KD--ALI---PSGTVI--
10    G--RN--SHIKRAIID--KN--ARIG--EN--VKI--INSDNV---QEAARE--TD--GYFIKS--G--IVTVI--KD--ALI---PSGTVI--
11    G--KN--SHIKRAIID--KN--ARIG--DN--VKI--INGDNV---QEAARE--TD--GYFIKS--G--IVTVI--KD--ALI---PSGTVI--
12    G--RN--SHIKRAIID--KN--ARIG--DN--VKI--INSDNV---QEAARE--TD--GYFIKS--G--IVTVI--KD--ALI---PSGTVI--
13    G--KN--SHIKRAIID--KN--ARIG--DN--VKI--INSDNV---QEAARE--TD--GYFIKS--G--IVTVI--KD--ALL---PSGTII--
14    G--EN--SHIKRAIID--KN--ARIG--DN--VKI--INTDNV---QEAARE--TD--GYFIKS--G--IVTVI--KD--ALI---PSGTVI--
15    G-----------------N--ARIG--DD--VKI--INSDNV---QEAARE--TD--GYFIKS--G--IVTII--KD--AMI---PSGTVI--
16    G--KN--SHIKRAIID--KN--ARIG--DD--VKI--INSDNV---QEAARE--TE--GYFIKS--G--IVTVI--KD--ALI---PSGTVI--
17    G--KN--SHIKRAIVD--KN--ARIG--EN--VKI--INSDNV---QEAARE--TE--GYFIKS--G--IVTII--KD--ALI---PSGTVI--
18    G--KN--SHIRRAIID--KN--ARIG--DN--VKI--INSDNV---QEAARE--TE--GYFIKS--G--IVTVI--KD--ALI---PSGTVI--
19    G--RN--SHIKRAIVD--KN--ARIG--EN--VKI--INSDNV---QEAARE--TD--GYFIKS--G--IVTVI--KD--ALI---PSGTVI--
20    G--RN--SHIKRAIID--KN--ARIG--ED--VKI--VNGDNV---QEAARE--TD--GYFIKS--G--IVTVI--KD--ALI---PSGTVI--
21    G--RN--SHIKRAIID--KN--ARIG--EN--VKI--VNGDNV---QEAARE--TD--GYFIKS--G--IVTVI--KD--ALI---PSGTII--
22    G--KN--SHIKRAIID--KN--ARIG--NN--VKI--VNRDSV---QEAARE--TD--GYFIKS--G--IDTII--KD--ALI---PSGTII--
23    G--KN--SHIKRAIID--KD--ARIG--DN--VKI--VNSDSV---QEAARE--TD--GYFIKS--G--IVTII--KD--ALI---PSGTII--
24    G--KN--SHIKRAIID--KN--ARIG--DN--VKI--IKSDNV---QETARE--TD--GYFIKS--G--IVTVI--KD--AWI---PSGTVI--
25    G--RN--SHIKRAIID--KN--ARIG--ND--VKI--INNDNV---QEAARE--TE--GYFIKS--G--IVTII--KD--ALI---PSGTII--
26    G--RN--SHIKRAIID--KN--ARIG--DN--VKI--INSDDV---QEAARE--TD--GYFIKS--G--IVTVI--KD--ALI---PSGTVI--
27    G--KN--SHIKRAIID--KN--VRIG--EN--VKI--INSDNV---QEAARE--TD--GYFIKS--G--IVTVI--KD--ALI---PSSTII--
28    G--KN--THIKRAIID--KN--ARIG--EN--VKI--VNGDNV---QEAARE--TD--GYFIKS--G--IVTVI--KD--ALI---PSGTMI--
29    G--RN--SHVKRAIID--KN--ARIG--EN--VKI--LNSDNV---QEAARE--TD--GYFIKS--G--IVTVI--KD--ALI---PSGTVI--
30    G--KN--SHIRRAIID--KN--ARIG--DD--VKI--INSDNV---QEAARE--TE--GYFIKS--G--IVTVI--KD--ALI---PSGTVI--
31    G--KN--SHIKRAIVD--KN--ARIG--EN--VKI--INSDNV---QEAARE--TE--GYFIKS--G--IVTII--KD--ALI---PSGTVL--
32    G--KN--SHIRKAIID--KN--ARIG--EN--VKI--INFDNV---QEAVRE--TE--GYFIKS--G--IVTVI--KD--ALI---PSGTII--
33    G--KN--SCIRRAIID--KN--ARIG--DN--VKI--LNADNV---QEAAME--TD--GYFIKG--G--IVTVI--KD--ALL---PSGTVI--
34    G--KN--SCIRRAIID--KN--ARIG--DN--VKI--LNADNV---QEAARE--TD--GYFIKG--G--IVTVI--KD--ALL---PSGTVI--
35    G--KN--SCIRRAIID--KN--ARIG--DN--VKV--FQTDVP---TVKNIN--LD---FSL----G--YYIIL--DM--PFC---PLIFNIYF
36    G--KN--SHIRKAIID--KN--ARIG--DN--VKI--LNADNV---QEAARE--TD--GYFIKG--G--IVTVI--KD--ALL---PSGTVI--
37    G--KN--CHIRRAIID--KN--ARIG--DN--VKI--INVDNV---QEAARE--TD--GYFIKS--G--IVTVI--KD--ALL---PSGTVI--
38    G--KN--AHIRKAIID--KN--ARIG--EN--VKI--INVDNI---QEASRE--TD--GYFIKS--G--IVTVI--KD--ALI---PSGTVI--
39    G--KN--CHIRRAIID--KN--ARIG--DN--VKI--INVDNV---QEAARE--TD--GYFIKS--G--IVTVI--KD--ALL---PSGTVI--
41    G--KN--SHIKRAIID--KN--ARIG--DN--VKI--INVDNV---QEAARE--TD--GYFIKS--G--IVTVI--KD--ALL---PSGTVI--
42    G--KN--SHIKRAIID--KN--ARIG--DN--VKI--INVDNV---QEAARE--TD--GYFIKS--G--IVTVI--KD--ALL---PSGTVI--
43    G--KN--AHIRKAIID--KN--ARIG--EN--VKI--INFDNV---QEAVRE--TE--GYFIKS--G--IVTVI--KD--ALI---PSGTII--
44    G--KN--THIKKAIID--KN--ARIG--EN--VKI--INVDDI---QEASRE--SD--GYFIKS--G--IVTVI--KD--ALI---PSGTVI--
45    G--KN--SHIKRAIID--KN--ARIG--DN--VMI--INVDNV---QEAARE--TD--GYFIKS--G--IVTVI--KD--ALL---PSGTVI--
46    G--KN--SHIKRAIID--KN--ARIG--DN--VMI--INVDNV---QEAARE--TD--GYFIKS--G--IVTVI--KD--ALL---PSGTVI--
47    G--KN--AHIRKAIID--KN--ARIG--EN--VKI--INVDDI---QEASRE--SD--GYFIKS--G--IVTVI--KD--ALI---PSGTVI--
48    G--KN--SHIKRAIID--KN--ARIG--DN--VMI--INVDNV---QEAARE--TD--GYFIKS--G--IVTVI--KD--ALL---PSGTVI--
49    G--EN--TKIRKCIID--KN--AKIG--KN--VSI--INKDGV---QEADRP--EE--GFYIRS--G--IIIIL--EK--ATI---RDGTVI--
50    G--PN--TKIQNCIID--KN--AKIG--KD--VVI--LNKEGV---EEADRS--AE--GFYIRS--G--ITVIM--KN--ATI---KDGTVI--
51    G--EN--TRIKDCIID--KN--ARIG--KN--VVI--ANSEGV---QEADRS--SE--GFYMAS--G--ITVIS--KN--STI---PDGTVI--
52    G--EN--TKIQECIID--KN--ARVG--KN--VII--ANSEGI---QEADRS--SD--GFYIRS--G--ITVIL--KN--SVI---KDGVVI--
53    G--QN--TKIKNCIID--KN--AKIG--KN--VVI--ANADGV---EEGDRP--EE--GFHIRS--G--ITVVL--KN--ATI---RDGLHI--
54    G--RD--TKIRKCIID--KN--AKIG--KN--VVI--MNKDDV---KEADRP--EE--GFYIRS--G--ITVVV--EK--ATI---KDGTVI--
55    G--KD--TKIRKCIID--KN--AKIG--KN--VII--MNKGDV---QEADRP--EE--GFYIRS--G--ITVIV--EK--ATI---QDGTVI--
56    G--EN--TKIRKCIID--KN--AKIG--KN--VSI--INKDGV---QEADRP--EE--GFYIRS--G--IIIIA--EK--ATI---RDGTVI--
57    G--PN--TKIQKCIID--KN--AKIG--KD--VVI--LNKQGV---EEADRS--AE--GFYIRS--G--ITVIM--KN--ATI---KDGTVI--
58    G--EN--TRIKECIID--KN--ARIG--KN--VVI--ANSEGV---QEADRS--SE--GFYIRS--G--ITVIL--KN--STI---PDGTVI--
59    G--EN--TKIRKCIID--KN--AKIG--KN--VSI--INKDGV---QEADRP--EE--GFYIRS--G--IIIIS--EK--ATI---RDGTVI--
60    G--PN--TKIQKCIID--KN--AKIG--KD--VVI--LNKQGV---EEADRS--AE--GFYIRS--G--ITVIM--KN--ATI---KDGTVI--
61    G--EN--TKIKECIID--KN--ARVG--KN--VII--ANSEGI---QEADRS--SD--GFYIRS--G--ITVIL--KN--SII---KDGVVI--
62    G--QN--TKIRNCIID--KN--AKIG--KN--VVI--ANAEGV---EEGDRP--EE--GFHIRS--G--ITVVL--KN--ATI---RDGLHI--
```

63 G--RD--TKIRKCIID--KN--AKIG--KN--VMI--LNKDDV---KEADRP--EE--GFYIRS--G--ITVVV--EK--ATI---KDSTVI--
64 G--RD--TKVRKCIID--KN--AKIG--KN--VII--MNKGDV---QEADRP--EE--GFYIRL--G--ITVIV--EK--ATI---QDGTVI--
66 G--KN--TKIVNCIID--KN--ARIG--NN--VII--ANKDNV---QEADRP--SE--GFYIRS--G--ITVVL--KE--SVI---SNGTII--
67 G--EN--TKIKDCIID--KN--ARIG--KN--VVI--ANSEGI---QEADRS--SE--GFYIRS--G--VTIVL--KN--SVI---EDGFII--
68 G--EN--TRIRNCIID--KN--ARIG--RN--VII--ANTDGV---QEADRP--AE--GFYIRS--G--IVVVV--KN--ATI---EDGTVI--
69 G--EN--TKIRNCIID--KN--ARIG--RN--VII--ANTDGV---QEADRP--ME--GFYIRS--G--IVVVA--NN--ATI---EDGTVI--
70 G--RN--TKIRNCIID--KN--AKIG--KD--VII--MNKDGV---QEADRP--ED--GFYIRS--G--ITVIL--EK--ATI---EDGTVI--
71 G--RN--TKIRNCIID--KN--AKIG--KD--VII--MNKDGV---QEADRP--ED--GFYIRS--G--ITVIL--EK--ATI---EDGTVI--
72 G--EN--TKIKDCIID--KN--ARIG--KN--VVI--ANSEGI---QEADRS--SE--GFYIRS--G--VTIVL--KN--SVI---EDGFII--
73 G--AN--TKIRNCIID--KN--AKIG--RN--VII--ANTDGV---QEADRA--KE--GFYIRS--G--ITVTL--KN--ATI---KDGTVI--
74 G--RN--TKIRNCIID--KN--AKIG--KD--VII--ANKDGV---QEADRP--ED--GFYIRS--G--ITIIM--EK--ATI---EDGTVI--
75 G--EN--TKIRNCIID--KN--AKIG--RN--VVI--ENIDGV---QEADRA--KE--GFYIRS--G--ITITL--KN--ATI---KDGTVI--
76 G--RN--TKIRNCIID--KN--AKIG--KD--VII--ANKDGV---QEADRP--ED--GFYIRS--G--ITIIM--EK--ATI---EDGTIV--
77 G--KN--TKIVNCIID--KN--ARIG--NS--VII--ANKDNV---QEADKP--TD--GFYIRS--G--ITVVL--KD--SVI---SNDTII--
80 G--SN--SKVRKCIID--KN--ARIG--KD--VII--MNKDGV---QEADRP--ED--GFYIRS--G--ITIVM--EK--ATI---EDGTVI--
81 G--KD--TKIMNCIID--KN--ARIG--KN--VII--ANKEGV---QEAERP--SE--GFYIRS--G--ITVVL--KN--SVI---KDGTII--
82 G--RN--TKIRNCIID--KN--AKIG--KD--VII--MNKDGV---QEADRE--EE--GFYIRS--G--ITIIS--EK--ATI---EDGTVI--
84 G--EN--TKIKECIID--KN--ARIG--KN--VVI--ANSEGI---QEADRS--ME--GFYIRS--G--VTVIL--KN--SVI---QDGTVI--
87 G--EN--TKIRECIID--KN--ARIG--KN--VVI--ANSEGI---QEADRS--SE--GFYIRS--G--VTIIL--KN--SVI---QDGFVI--
88 G--QE--TKIMNCIID--KN--ARIG--KN--VVI--ANKDHV---EEADRP--SE--GFYIRS--G--ITVVL--KN--SEI---KDGTII--
89 G--RN--TKIKNCIID--KN--AKIG--KD--VVI--VNKDGV---QEADRP--EE--GFYIRS--G--ITIIM--EK--ATI---EDGTVI--
90 G--QN--TKIRNCIID--KN--AKIG--RG--VVI--TNADGV---QEAERP--EE--GFYIRS--G--ITVIM--EN--ATI---NDGTII--
91 G--QN--TRIRNCIID--KN--AKIG--RD--VVI--ANADGV---QEADRP--SE--GFYIRS--G--ITVIL--KN--ATI---NDGTII--
92 G--KG--TKIMNCIID--KN--ARIG--KN--VVI--TNKDKV---EEADRP--SE--GFYIRS--G--ITVVL--KN--SVI---MDETII--
94 G--EN--TRIKDCIID--KN--ARIG--KN--VVI--SNSEGI---QEADRS--LE--GFYIRS--G--ITIIL--KN--FTI---KDGFVI--
95 G--RN--TKIRNCIID--KN--AKIG--KD--AVI--VNKDGV---QEADRP--DD--GFYIRS--G--ITIIL--EK--ATI---KDGTVI--
96 G--QN--TKIRNCIID--KN--AKIG--KD--VII--TNADGV---QEADRP--SE--GFYIRS--G--ITAVL--KN--AAI---KDGTLI--
97 G--RN--TKIRNCIID--KN--AKIG--KD--VII--TNKDGV---QEADRE--EK--GFYIRS--G--ITIIL--EK--ATI---EDGTVI--
98 G--QN--TKIRNCIID--KN--AKIG--KD--VII--TNADGV---QEADRP--SE--GFYIRS--G--ITAVL--KN--ATI---KDGTII--
99 G--EN--TTIQKCIID--KN--ARIG--KK--VVI--SNSEGV---DEADRT--SE--GFYIRS--G--ITVVL--KN--AII---ADGLVI--
100 G--RN--TKIRNCIID--MN--ARIG--KN--VVI--TNSKGI---QEADHP--EE--GYYIRS--G--IVVIL--KN--ATI---NDGSVI--
101 G--EN--TKISNCIID--MN--CQGW--KE--RLH--NKQRGRS--KSPDRP--GR--RILIRS--G--IVVVL--KN--ATI---KDGTVI--
102 G--EN--TKIRNCIID--KN--ARIG--KN--VVI--MISENV---QEADRP--AE--GYYIRS--G--ITVVL--KN--AVI---LNGTKI--
103 G--EN--AKISNCIID--MN--ARIG--RD--VII--ANSEGV---EEADRA--EE--GYYIRS--G--IVVIL--KN--ATI---KDGTVV--
104 G--EN--TKIRNCIID--KN--ARIG--KN--VVI--MNSENV---QEADRP--SE--GYYIRS--G--ITVVL--KN--AVI---PDDTII--
105 G--EN--TTIQNCIID--KN--ARIG--KN--VTI--ANSEGV---QEADRT--SE--GFHIRS--G--ITVVL--KN--SVI---ADGLVI--
106 G--EN--TKIQNCIID--KN--ARIG--KN--VTI--SNSEGV---QEADRT--SE--GFYIRS--G--ITIVL--KN--SII---ADGLVI--
107 G--EN--TKINNCIID--MN--ARVG--RN--VVI--TNSEGV---QESDRP--EE--GYYIRS--G--IVVIL--KN--ATI---KDGKVI--
108 G--EN--TIIRNCIID--KN--ARIG--KN--VMI--MNSQNV---QEAERP--LE--GFYIRS--G--ITVVL--KN--AVI---PDGTVI--
109 G--EN--TKIRNCIID--MN--ARIG--RN--VII--ANTQGV---QESDHP--EE--GYYIRS--G--IVVIL--KN--ATI---KDGTVI--
110 G--EN--TKISNCIID--MN--ARVG--RN--VSI--TNTEGV---QEADRP--EL--GYYIRS--G--IVVIL--KN--ATI---KDGTVI--
111 G--GN--TKIRNCIID--IN--ARIG--KN--VVI--TNSKGI---QEADHP--EE--GYYIKS--G--IVVIL--KN--ATI---KDGSVI--
112 G--EN--TTIQKCIID--KN--ARIG--KN--VII--SNSEGV---VEADRT--SE--GFYIRT--G--VTVVL--KN--SII---ADGLVI--
113 G--EN--TKISNCIID--MN--ARIG--RD--VVI--SNKEGV---QEADRP--EE--GYYIRS--G--IVVIQ--KN--ATI---KDGTVV--
114 G--EN--TSIQNCIID--KN--ARIG--KN--VTI--ANTEGV---QESDRT--SE--GFHIRS--G--ITVVL--KN--SVI---ADGLVI--
115 G--EN--TKISNCIID--MN--ARIG--RD--VVI--SNKEGV---QEADRP--EE--GYYIRS--G--IVVIQ--KN--ATI---KDGTVV--
116 G--EN--TKIKDCIID--KN--ARIG--KN--ATI--SNVDGV---QEADRS--AE--GFYTRS--G--ITVIL--KN--STI---PDGFAI--
117 G--EN--TKISNCIID--MN--ARIG--RD--VVI--SNKEGV---QEADRP--EE--GYYIRS--G--IVVIQ--KN--ATI---KDGTVV--
118 G--EN--TSIQNCIID--KN--ARIG--KN--VTI--ANAEGV---QESDRA--SE--GFHIRS--G--ITVVL--KN--SVI---ADGLVI--
119 G--EN--TKISNCIID--MN--ARIG--RD--VVI--SNKEGV---QEADRP--EE--GYYIRS--G--IVVIQ--KN--ATI---KDGTVV--
120 G--EN--TKISNCIID--MN--ARIG--RD--VVI--SNKEGV---QEADRP--EE--GYYIRS--G--IVVIQ--KN--ATI---KDGTVV--
121 G--QG--TTVKRAILD--KN--TRIG--SN--VTI--VNKDHV---EEADRP--EL--GFYIRN--G--IVVVV--KN--ASI---PDGTVI--
122 G--NN--TTIRRAIVD--KN--ARIG--RH--VQI--INKDHV---QEAERE--ED--GFYIRG--G--ITVIL--KN--AVI---QDGTII--
123 G--TD--TIIRRAIID--KN--ACIG--HD--VKI--INKDNV---QEAERE--NQ--GFYIRS--G--IVVVL--KG--AVI---ADGTII--
124 G--KG--STIRRAIID--KN--ARIG--RN--VTI--VNKENI---EESNRE--DE--GFYIRN--G--IVVAI--KN--AII---PDGTVI--
125 G--SN--TTIDHAIID--KN--ARIG--CN--VKI--INKDNV---SEAEKE--DQ--GFYIRS--N--IITIL--KD--AVI---PHDTVI--
126 G--RG--TTVKRAILD--KN--VRIG--RD--VTI--VNKDRV---EEADRP--EL--GFYIRN--G--IVVVV--KN--ATI---ADGTVI--
127 G--RG--TTVKRAILD--KN--ARIG--DN--VTI--VNKDNV---EEADRP--EL--GFYIRN--G--IVVVV--KN--ASI---PDHSVI--
128 G--RG--TTVRRAILD--KN--VRIG--RN--VTI--VNKDGI---EEADRP--EL--GFYIRN--G--IVVVE--KN--ATI---ADGTVI--
129 G--AD--TTIRRAIVD--KN--ARIG--SN--VTI--TNKEDV---EQAERE--EL--GFYIRS--G--IVTIL--KN--AVI---PDGTVI--
130 G--EG--STVKRAILD--KN--TRIG--RN--VTI--VNKDRV---EEADRP--EL--GFYIRN--G--IVVVC--KN--ATI---PDGMVI--
131 G--PG--TTVKRAILD--KN--TRIG--SN--VSI--INKDHV---EEADRS--DL--GFYIRN--G--IVVVQ--KN--ATI---QDGTVI--
132 G--KG--TTVKRAILD--KN--ARIG--SN--VTI--VNKDHV---EEADRP--EH--GFYIRN--G--IVVVV--KN--ASI---PDGTVI--
133 G--ER--CVLQKAIID--KN--ARIG--ND--VRI--LNKERP---DSADHP--ER--GFYIRH--G--IVIVP--KD--TVI---PDGTVI--
134 G--EG--STIKRAILD--KN--ARIG--DN--VVI--VNKDRV---EEADKP--DV--GFYIRN--G--IVVVV--KN--ATI---ANGTII--
135 G--QG--TTVKRAILD--KN--TRIG--EN--VTI--INKDRI---EEADRA--DQ--GFYIRN--G--IVVVV--KN--ASI---LDGTII--
136 G--AN--SVIRRAIVD--KN--ACIG--RD--VKI--INKDNV---EESNRE--DQ--GFYIRS--G--VVVII--KN--AVI---PDGTII--
137 G--EG--STIRRAIVD--KN--ARIG--RN--VTI--VNKENI---DESNQE--ES--GFYIRN--G--IVVIL--KN--ATI---ADGTVI--
138 G--TD--TIIRGAIID--KN--ARIG--HD--VKI--VNKDNV---QEAERE--NQ--GFYIRS--G--IVVVL--KN--AVI---PDGTII--
139 G--EG--TTVKGAILD--KN--TRIG--NN--VTI--VNKDHV---EEADRA--DE--GFYIRN--G--IVVVV--KN--ATI---SDGTVI--
140 G--AN--SHIVNAIVD--KN--ARIG--RN--VRI--LNKDHV---TEAQRE--EE--GIWISN--G--IVTII--KD--SVI---PDNTII--
141 G--AN--SHIVNAIVD--KN--ARIG--RN--VRI--LNKDHV---SEAQRE--EE--GIWISN--G--IVTII--KD--SVI---PDNTVI--
142 G--QG--TTVKGAILD--KN--TRIG--NN--VTI--VNKDHV---EEADRA--DE--GFYIRN--G--IVVVV--KN--ATI---SDGTVI--
143 G--QG--TTVKRAILD--KN--ARIG--SN--VTI--VNKDHV---EEADRS--DQ--GFYIRN--G--IVVVV--KN--ATI---QDGTVI--
144 G--PD--TIIRRAIID--KN--ARIG--HD--VKI--INKDNV---QEADRE--SQ--GFYIRS--G--IVVVL--KN--AVI---TDGTII--
145 G--QG--TTVKRAILD--KN--ARIG--EN--VAI--VNKDNV---EEADRP--EE--GFYIRN--G--IVVVV--KN--ATI---SDGTII--
146 G--EG--TTVKRAILD--KN--TRIG--DN--VVI--INKDRV---EEADKP--EL--GFYIRN--G--IVVVV--KN--ATI---ANGTVI--

```
147  G--QG--TTVKRAILD--KN--ARIG--DN--VTI--VNKDNV---EEADRA--DQ--GFYIRN--G--IVVIV--KN--ATI---PDGTII--
148  G--EG--TTVKRAILD--KN--TRIG--DN--VVI--INKDRV---EEADKP--EL--GFYIRN--G--IVVVV--KN--ATI---ANGTVI--
149  G--VG--STIKRAILD--KN--ARIG--DN--VVI--VNKDRV---EEADKP--EL--GFYIRN--G--IVVVV--KN--ATI---ANGTII--
150  G--EG--TTVKGAILD--KN--ARIG--KN--VTI--VNKDRV---EEADRP--DQ--GFYIRN--G--IIVVV--KN--ASI---ADDTVI--
151  G--AG--TTIRRAIID--KN--ARIG--RN--VLI--INKDRI---EEAERE--DE--GFLIRS--G--IVVVI--KN--ATI---PDGTVI--
152  G--EG--TTVKRAILD--KN--TRIG--DN--VVI--INKDRV---EEADKP--EL--GFYIRN--G--IVVVV--KN--ATI---ANGTVI--
153  G--SG--TTIRRAIID--KN--ARIG--KN--VMI--VNKENV---QEANRE--EL--GFYIRN--G--IVVVI--KN--VTI---ADGTVI--
154  G--EG--STIRRAIVD--KN--ARIG--RN--VTI--VNKENI---DESNQE--ES--GFYIRN--G--IVVIL--KN--ATI---ADGTVI--
155  G--SG--STIRRAIVD--KN--AHIG--QN--VQI--VNKDHV---EEADRE--DL--GFMIRS--G--IVVVV--KG--AVI---PDNTVI--
156  G--AD--TIVRRAIVD--KN--ACIG--RN--VKI--VNKDHV---EEANRE--SE--GFYIRN--G--IVVVL--KN--AVI---PDNTVI--
157  G--SG--STIRRAIVD--KN--AHIG--QN--VQI--VNKDHV---EEADRE--DL--GFMIRS--G--IVVVV--KG--AVI---PDNTVI--
158  G--KG--TTVKRAILD--KN--TRIG--SG--VSI--INKDNV---EEADRS--DQ--GFYIRN--G--IVVVQ--KN--ATI---ADGTVI--
159  G--PG--TTVKRAILD--KN--TRIG--SN--VSI--INKDHV---EEADRS--DL--GFYIRN--G--IVVVQ--KN--ATI---QDGTVI--
160  G--EN--TVIRKAIVD--KN--ARIG--KN--VKI--VNKAQV---EEANHE--DE--GFYIRS--G--IVVIL--KN--AII---PDGTEI--
161  G--EN--TKISRAIID--KN--ARIG--RN--VKI--VNKDNV---EESNQE--EH--GFYIRS--G--IVVVL--KN--AEI---PDNTII--
162  G--AE--TKIRGAIID--KN--ARIG--CN--VQI--INKDNV---EEAQRE--EE--GFIIRS--G--IVVVL--KN--ATI---PDGTVI--
163  G--PD--TIIRRAIID--KN--ARIG--HD--VKI--INKDNV---QEADRE--SQ--GFYIRS--G--IVVVL--KN--AVI---TDGTII--
164  G--SN--TTIDHAIID--KN--ARIG--CN--VKI--INKDNV---SEAEKE--DQ--GFYIRS--N--IITVV--KD--AVI---PHDTVI--
165  G--KG--STIRRAIVD--KN--ARIG--QN--VTI--VNKENI---EESNRE--DD--GFYIRN--G--IVVVI--KN--AVI---PDGTVI--
166  G--EG--TTIRRAIID--KN--ARIG--KN--VMI--VNKENV---EESNRE--EL--GYYIRS--G--ITVVL--KN--AVI---PDGTVI--
167  G--EG--TTVKRAILD--KN--TRIG--DN--VVI--INKDRV---EEADKP--EL--GFYIRN--G--IVVVV--KN--ATI---ANGTVI--
168  G--QG--TTVKRAILD--KN--ARIG--DN--VTI--VNKDNV---EEADRA--DQ--GFYIRN--G--IVVIV--KN--ATI---PDGTII--
169  G--RG--TTVKKAILD--KN--VRIG--SN--VSI--INKDNV---EEADRA--EQ--GFYIRG--G--IVVIT--KN--ASI---PDGMVI--
170  G--KG--TTVKRAILD--KN--ARIG--SN--VTI--VNKDHV---EEADRP--EH--GFYIRN--G--IVVVV--KN--ASI---PDGTVI--
171  G--SG--STIRRAIVD--KN--ARIG--SN--VLI--VNKDRV---EEANRE--DL--GFYVRS--G--IVVIF--KN--ATI---PDGTVI--
172  G--EG--TTVKRAILD--KN--TRIG--DN--VVI--INKDRV---DEADKP--EL--GFYIRN--G--IVVVV--KN--ATI---ANGTVI--
173  G--SG--TTVRGAILD--KN--VRIG--RD--VTI--VNKDRV---EEADRP--EL--NFYIRN--G--IVVVV--KN--GTI---ADGTVI--
174  G--AG--TTIRRAIID--KN--AHIG--KN--VLI--INKDRI---EEADRE--DQ--GFLIRN--G--IVVVM--KN--ATI---PDGTVI--
175  G--TN--TIIRRAIID--KN--ARIG--HD--VKI--INKDNV---QEAERE--KQ--GFFIRS--G--IVVVL--KN--AVI---PDGTII--
176  G--KD--TVIRRAIID--KN--ACIG--NN--VKI--FNKDRV---EEANCE--SE--GFYIRN--G--IVVVL--KN--AVI---PHGAVI--
177  G--SN--TIIRRAIID--KN--THIG--CD--VQI--VNKDNV---QEAERE--SQ--GFYIRS--G--IVVVL--KN--AVI---PDGTII--
178  G--KG--STIRRAIVD--KN--ARIG--QN--VTI--VNKENI---EESNRE--DD--GFYIRN--G--IVVVI--KN--AVI---PDGTVI--
179  G--SN--TTIDHAIID--KN--ARIG--CN--VKI--INKDNV---SEAEKE--DQ--GFYIRS--N--IITVV--KD--AVI---PHDTVI--
180  G--EG--TTVKRAILD--KN--ARIG--KN--VTI--VNKDHV---EEADRP--EH--GFYIRN--G--IVVVV--KN--ASI---ADDTVI--
181  G--KN--SLIKHAIID--KN--ARIG--EN--VII--LNKNDI---QESSRE--DE--GFYICD--G--IVVII--KN--AVI---QSGTVI--
182  G--AD--TTIRRAIVD--KN--ARIG--RN--VQI--INKDRV---EEANRE--NQ--GFYIRS--G--IIVVL--KN--ATI---PDGTII--
183  G--AQ--TRIRRAIVD--KN--ARIG--RH--VQI--INKDRV---EEAERE--DQ--GFYIRS--G--IVVVL--KN--AII---SDGTII--
184  G--TD--TIIRRAIID--KN--ARIG--HN--VRI--INKDNV---QEAERE--KQ--GFYIRS--G--IVVVL--KN--AVI---PDGTII--
185  G--TD--TIIRRAIID--KN--ARIG--HN--VRI--INKDNV---QEAERE--KQ--GFYIRS--G--IVVVL--KN--AVI---PDGTII--
186  G--NN--TTIRRAIVD--KN--ARIG--RH--VQI--INKDHV---QEAERE--ED--GFYIRG--G--ITVIL--KN--AVI---PDGTII--
187  G--KG--STIRRAIVD--KN--ARIG--TN--VNI--VNKENI---EESNRE--DD--GFYIRN--G--IVVVI--KN--AVI---PDGTVI--
188  G--KG--TTVRRAILD--KN--TRIG--SG--VSI--INKDNV---EEADRS--DQ--GFYIRN--G--IVVVQ--KN--ATI---ADGTVI--
189  G--EG--CTITNAIID--KN--ARIG--KN--CII--TNASGI---DDLEDE--EN--GVYIRS--G--IVTIL--RN--ATI---PDGTVI--
190  G--AG--TVIRRAIVD--KN--ARIG--MD--CQL--INKDNV---QEANEE--EK--GYIIKD--G--IIVIV--KD--SYI---PNGTII--
191  G--EG--CSISNAIID--KN--ARIG--KN--CII--TNAAGV---EDLEDE--EN--GIYIRS--G--IVTIL--RN--ATI---PDGTVI--
192  G--AG--TTIKRAIID--KN--ARIG--MD--CQI--INKDNV---QEANHE--DK--GYIIKD--G--IVVIC--KD--AII---PNGTVI--
193  G--AN--SVITNAIID--KN--ARVG--KN--VKI--VNKEGV---TEGTRE--AE--GIYIRS--G--IVVID--KG--ALV---PDNTTI--
194  G--DG--SIIRRAIVD--KN--ARIG--PK--CQI--INKDGV---KEANRE--DQ--GFVIKD--G--IVVVI--KD--SHI---PAGTII--
195  G--EG--TIIENAIID--KN--ARVG--KN--CVI--TNAAGV---EDLADE--ER--GVFIRN--G--IITIL--RN--CTI---PDGTII--
196  G--AG--TKIRKAIID--KN--ARIG--EN--CQI--LNEAGV---MDKDCE--NE--GYIIRD--G--IIVVI--KD--AVI---KPGTVI--
197  G--DG--SVVRKAIID--KN--ARIG--PK--CQI--INKDGV---KEANRE--EQ--GFVIKD--G--IVVVI--KD--SCI---PAGTII--
198  G--AN--SIISNAIID--KN--ARVG--KN--VRI--VNKDGV---SEGTRE--SE--GIYIRS--G--IVVID--KG--AKV---PDNATI--
199  G--EN--TIIENAIID--KN--ARVG--KN--CVI--TNKDNI---EDLADE--ER--GVFIRN--G--IVTIL--RN--CTI---PDGTVI--
200  G--AG--TKIRKAIID--KN--ARIG--EN--CQI--LNEAGV---MDKDCE--SE--GYIIRD--G--IIVVI--KD--AVI---KAGTVI--
201  G--QN--AKIRNCILD--KN--VRIG--KN--VVI--ANKDNV---QEAEKP--SE--GYYIRS--G--ITVIL--KN--ATI---ADGTVI--
203  G--KG--SVVRRAIVD--KN--ARIG--QN----I--INKDGV---QEAARE--TD--GFFINC--G--IVTVI--KD--AVI---PNSVVI--
204  G--KG--SVVRRAIVD--KN--ARIG--QN----I--INKDGV---QEAARE--TD--GFFINC--G--IVTVI--KD--AVI---PNSVVI--
206  G--EH--TTMRNCLVD--KN--ARIG--SH--VII--TNTDGV---QEAERP--SE--GIYIRS--G--ITVVV--KN--SIV---KDGTVI--
207  G--EH--TTMRNCLVD--KN--ARIG--SH--VII--TNTDGV---QEAERP--SE--GIYIRS--G--ITVVV--KN--SIL---KDGTVI--
208  G--QN--AKIRNCILD--KN--VRIG--KN--VVI--ANKDNV---QEAEKP--SE--GYYIRS--G--ITVIL--KN--ATI---ADGTVI--
209  G--QN--AKIRNCILD--KN--VRIG--KN--VVI--ANKDNV---QEAEKP--SE--GYYIRS--G--ITVIL--KN--ATI---ADGTVI--
```
