# Supplementary material for: The ancestral activation promiscuity of ADP-glucose pyrophosphorylases from oxygenic photosynthetic organisms
Source: BMC Evol Biol. 2013 Feb 21;13:51. doi: 10.1186/1471-2148-13-51 (PMC3585822; doi:10.1186/1471-2148-13-51)
Supplement: Additional file 2: Table S1 — Data of sequences used for the phylogenetic tree. The table contains the number used for each sequence, the corresponding NCBI accession number and annotation, the name of the organism, and the taxonomic group. Colors used are the same as in the phylogenetic tree (Figure 1). [file 1471-2148-13-51-S2.pdf]

Table S1

Number	Accession	Annotation	Organism	Taxonomy
1	62738704	Chain A, Crystal Structure Of Potato Tuber Adp-Glucose Pyrophosphorylase	*Solanum tuberosum*	dicot
2	27819107	ADP-glucose pyrophosphorylase small subunit	Solanum tuberosum	dicot
3	77416911	ADP-glucose pyrophosphorylase small subunit-like	Solanum tuberosum	dicot
4	556351	ADP-glucose pyrophosphorylase small subunit	Solanum tuberosum	dicot
5	15238933	glucose-1-phosphate adenylyltransferase small subunit	*Arabidopsis thaliana*	dicot
6	297791999	hypothetical protein ARALYDRAFT_494882 agps	*Arabidopsis lyrata* subsp. lyrata	dicot
7	357495273	Glucose-1-phosphate adenylyltransferase	*Medicago truncatula*	dicot
8	357462397	Glucose-1-phosphate adenylyltransferase	*Medicago truncatula*	dicot
9	356552274	PREDICTED: glucose-1-phosphate adenylyltransferase small subunit, isoform 1	*Glycine max*	dicot
10	356501687	PREDICTED: glucose-1-phosphate adenylyltransferase small subunit, isoform 2	*Glycine max*	dicot
11	224131934	predicted protein	*Populus trichocarpa*	dicot
12	255567204	glucose-1-phosphate adenylyltransferase, putative	*Ricinus communis*	dicot
13	225447450	PREDICTED: hypothetical protein	*Vitis vinifera*	dicot
14	13487709	ADP-glucose pyrophosphorylase small subunit	*Brassica rapa* subsp. pekinensis	dicot
15	556622	ADP-glucose pyrophosphorylase	*Beta vulgaris* subsp. vulgaris	dicot
16	1237080	ADP-glucose pyrophosphorylase	*Pisum sativum*	dicot
17	1237082	ADP-glucose pyrophosphorylase	*Pisum sativum*	dicot
18	13487787	ADP-glucose pyrophosphorylase small subunit CagpS1	*Cicer arietinum*	dicot
19	16950559	ADP-glucose pyrophosphorylase small subunit CagpS2	*Cicer arietinum*	dicot
20	2642636	ADP-glucose pyrophosphorylase small subunit	*Citrullus lanatus* subsp. vulgaris	dicot
21	2625084	ADP-glucose pyrophosphorylase small subunit	*Cucumis melo*	dicot
22	5917789	ADP-glucose pyrophosphorylase small subunit	*Citrus unshiu*	dicot
23	111660950	ADP-glucose pyrophosphorylase small subunit	*Citrus sinensis*	dicot
24	41350641	ADP-glucose pyrophosphorylase small subunit	*Fragaria* x ananassa	dicot

25	45505207	ADP-glucose pyrophosphorylase small subunit	*Ipomoea batatas*	dicot
26	45505205	ADP-glucose pyrophosphorylase small subunit	*Ipomoea batatas*	dicot
27	7671232	ADP-glucose pyrophosphorylase	*Perilla frutescens*	dicot
28	7671230	ADP-glucose pyrophosphorylase catalytic subunit	*Perilla frutescens*	dicot
29	29421116	ADP-glucose pyrophosphorylase small subunit PvAGPS1	*Phaseolus vulgaris*	dicot
30	440595	ADP-glucose pyrophosphorylase	*Vicia faba* var. minor	dicot
31	440593	ADP-glucose pyrophosphorylase	*Vicia faba* var. minor	dicot
32	162462257	ADP-glucose pyrophosphorylase small subunit	*Zea mays*	monocot
33	162461970	LOC541902	*Zea mays*	monocot
34	73747074	ADP-glucose pyrophosphorylase small subunit	*Zea mays*	monocot
35	23664353	Brittle 2	*Zea mays* subsp. mays	monocot
36	14582768	ADP-glucose pyrophosphorylase small subunit	*Zea mays*	monocot
37	115476014	Os08g0345800	*Oryza sativa* Japonica Group	monocot
38	115478426	Os09g0298200	*Oryza sativa* Japonica Group	monocot
39	125987830	GLGS_ORYSJ	*Oryza sativa* Japonica Group	monocot
40	357157910	PREDICTED: glucose-1-phosphate adenylyltransferase small subunit	*Brachypodium distachyon*	monocot
41	357145854	PREDICTED: glucose-1-phosphate adenylyltransferase small subunit, isoform 2	*Brachypodium distachyon*	monocot
42	357145851	PREDICTED: glucose-1-phosphate adenylyltransferase small subunit, isoform 1	*Brachypodium distachyon*	monocot
43	242048788	hypothetical protein SORBIDRAFT_02g020410	*Sorghum bicolor*	monocot
44	27464770	ADP-glucose pyrophosphorylase small subunit	*Hordeum vulgare* subsp. vulgare	monocot
45	51556842	ADP-glucose pyrophosphorylase small subunit a	*Hordeum vulgare*	monocot
46	1707940	GLGS_HORVU	*Hordeum vulgare*	monocot
47	52430025	ADP-glucose pyrophosphorylase small subunit	*Triticum aestivum*	monocot
48	21687	ADP-glucose pyrophosphorylase	*Triticum aestivum*	monocot
49	232166	GLGL1_SOLTU	*Solanum tuberosum*	dicot
50	1707929	GLGL2_SOLTU	*Solanum tuberosum*	dicot
51	1707932	GLGL3_SOLTU	*Solanum tuberosum*	dicot

52	14916987	GLGL1_ARATH	*Arabidopsis thaliana*	dicot
53	12644324	GLGL2_ARATH	*Arabidopsis thaliana*	dicot
54	17433716	GLGL3_ARATH	*Arabidopsis thaliana*	dicot
55	11386853	GLGL4_ARATH	*Arabidopsis thaliana*	dicot
56	1947084	ADP-glucose pyrophosphorylase large subunit agpl1	*Solanum lycopersicum*	dicot
57	1840114	ADP-glucose pyrophosphorylase large subunit agpl2	*Solanum lycopersicum*	dicot
58	1840116	ADP-glucose pyrophosphorylase large subunit agpl3	*Solanum lycopersicum*	dicot
59	1778434	ADP-glucose pyrophosphorylase large subunit agp-s1	*Solanum lycopersicum*	dicot
60	1778436	ADP-glucose pyrophosphorylase large subunit agp-s2	*Solanum lycopersicum*	dicot
61	297812109	hypothetical protein ARALYDRAFT_488807 agpl1	*Arabidopsis lyrata* subsp. lyrata	dicot
62	297845724	hypothetical protein ARALYDRAFT_472972 agpl2	*Arabidopsis lyrata* subsp. lyrata	dicot
63	297797902	hypothetical protein ARALYDRAFT_490693 agpl3	*Arabidopsis lyrata* subsp. lyrata	dicot
64	297821353	predicted protein agpl4	*Arabidopsis lyrata* subsp. lyrata	dicot
65	357467317	Glucose-1-phosphate adenylyltransferase large subunit	*Medicago truncatula*	dicot
66	356571037	PREDICTED: glucose-1-phosphate adenylyltransferase large subunit 2	*Glycine max*	dicot
67	356563435	PREDICTED: glucose-1-phosphate adenylyltransferase large subunit 1	*Glycine max*	dicot
68	356562361	PREDICTED: glucose-1-phosphate adenylyltransferase large subunit	*Glycine max*	dicot
69	356553863	PREDICTED: glucose-1-phosphate adenylyltransferase large subunit	*Glycine max*	dicot
70	356545193	PREDICTED: glucose-1-phosphate adenylyltransferase large subunit 1-like	*Glycine max*	dicot
71	356538761	PREDICTED: glucose-1-phosphate adenylyltransferase large subunit 1-like	*Glycine max*	dicot
72	356521967	PREDICTED: glucose-1-phosphate adenylyltransferase large subunit 1	*Glycine max*	dicot
73	356518710	PREDICTED: glucose-1-phosphate adenylyltransferase large subunit	*Glycine max*	dicot
74	356517038	PREDICTED: glucose-1-phosphate adenylyltransferase large subunit 1-like	*Glycine max*	dicot
75	356509672	PREDICTED: glucose-1-phosphate adenylyltransferase large subunit	*Glycine max*	dicot
76	356508352	PREDICTED: glucose-1-phosphate adenylyltransferase large subunit 1-like	*Glycine max*	dicot
77	356503982	PREDICTED: glucose-1-phosphate adenylyltransferase large subunit 2	*Glycine max*	dicot
78	357511621	Glucose-1-phosphate adenylyltransferase	*Medicago truncatula*	dicot

79	357480219	Glucose-1-phosphate adenylyltransferase	*Medicago truncatula*	dicot
80	357473317	Glucose-1-phosphate adenylyltransferase	*Medicago truncatula*	dicot
81	224128113	predicted protein	*Populus trichocarpa*	dicot
82	224103389	predicted protein	*Populus trichocarpa*	dicot
83	224117842	predicted protein	*Populus trichocarpa*	dicot
84	224100249	predicted protein	*Populus trichocarpa*	dicot
85	224095317	predicted protein	*Populus trichocarpa*	dicot
86	255585297	glucose-1-phosphate adenylyltransferase, putative	*Ricinus communis*	dicot
87	255552303	glucose-1-phosphate adenylyltransferase, putative	*Ricinus communis*	dicot
88	255548169	glucose-1-phosphate adenylyltransferase, putative	*Ricinus communis*	dicot
89	255543725	glucose-1-phosphate adenylyltransferase, putative	*Ricinus communis*	dicot
90	255538708	glucose-1-phosphate adenylyltransferase, putative	*Ricinus communis*	dicot
91	225458219	PREDICTED: hypothetical protein	*Vitis vinifera*	dicot
92	225437808	PREDICTED: hypothetical protein	*Vitis vinifera*	dicot
93	225434249	PREDICTED: hypothetical protein	*Vitis vinifera*	dicot
94	225432564	PREDICTED: hypothetical protein	*Vitis vinifera*	dicot
95	225428422	PREDICTED: hypothetical protein	*Vitis vinifera*	dicot
96	224085694	predicted protein	*Populus trichocarpa*	dicot
97	224080375	predicted protein	*Populus trichocarpa*	dicot
98	224062107	predicted protein	*Populus trichocarpa*	dicot
99	162460455	plastid ADP-glucose pyrophosphorylase large subunit	*Zea mays*	monocot
100	189027076	glucose-1-phosphate adenylyltransferase large subunit 1	*Zea mays*	monocot
101	162458350	glucose-1-phosphate adenylyltransferase large subunit 2	*Zea mays*	monocot
102	162463875	putative glucose-1-phosphate adenylyltransferase large subunit 3 precursor	*Zea mays*	monocot
103	357132398	PREDICTED: glucose-1-phosphate adenylyltransferase large subunit	*Brachypodium distachyon*	monocot
104	357119087	PREDICTED: glucose-1-phosphate adenylyltransferase large subunit 2	*Brachypodium distachyon*	monocot
105	357116651	PREDICTED: glucose-1-phosphate adenylyltransferase large subunit 1	*Brachypodium distachyon*	monocot

106	115455167	Os03g0735000	*Oryza sativa* Japonica Group	monocot
107	297604962	Os05g0580000	*Oryza sativa* Japonica Group	monocot
108	115471355	Os07g0243200	*Oryza sativa* Japonica Group	monocot
109	115438749	Os01g0633100	*Oryza sativa* Japonica Group	monocot
110	242088961	hypothetical protein SORBIDRAFT_09g029610	*Sorghum bicolor*	monocot
111	242053733	hypothetical protein SORBIDRAFT_03g028850	*Sorghum bicolor*	monocot
112	242033053	hypothetical protein SORBIDRAFT_01g008940	*Sorghum bicolor*	monocot
113	1279513	glucose-1-phosphate adenylyltransferase	*Hordeum vulgare* subsp. vulgare	monocot
114	2105137	ADP-glucose pyrophosphorylase large subunit	*Hordeum vulgare* subsp. vulgare	monocot
115	445623	ADP glucose pyrophosphorylase : SUBUNIT=L	*Hordeum vulgare*	monocot
116	22347636	ADP-glucose pyrophosphorylase large subunit	*Oncidium Goldiana*	monocot
117	32812836	ADP-glucose pyrophosphorylase large subunit	*Triticum aestivum*	monocot
118	89277026	plastid ADP-glucose pyrophosphorylase large subunit	*Triticum aestivum*	monocot
119	1707930	GLGL2_WHEAT	*Triticum aestivum*	monocot
120	121293	GLGL3_WHEAT	*Triticum aestivum*	monocot
121	87124328	ADP-glucose pyrophosphorylase	*Synechococcus* sp. RS9917	cyanobacteria
122	284051940	glucose-1-phosphate adenylyltransferase	*Arthrospira platensis* str. Paraca	cyanobacteria
123	119509469	glucose-1-phosphate adenylyltransferase	*Nodularia spumigena* CCY9414	cyanobacteria
124	126660345	glucose-1-phosphate adenylyltransferase	*Cyanothece* sp. CCY0110	cyanobacteria
125	126658160	glucose-1-phosphate adenylyltransferase	*Cyanothece* sp. CCY0110	cyanobacteria
126	318041355	glucose-1-phosphate adenylyltransferase	*Synechococcus* sp. CB0101	cyanobacteria
127	317969822	glucose-1-phosphate adenylyltransferase	*Synechococcus* sp. CB0205	cyanobacteria
128	87302823	glucose-1-phosphate adenylyltransferase	*Synechococcus* sp. WH 5701	cyanobacteria
129	119490200	glucose-1-phosphate adenylyltransferase	*Lyngbya* sp. PCC 8106	cyanobacteria
130	116074714	glucose-1-phosphate adenylyltransferase	*Synechococcus* sp. RS9916	cyanobacteria
131	116070673	glucose-1-phosphate adenylyltransferase	*Synechococcus* sp. BL107	cyanobacteria
132	88808518	glucose-1-phosphate adenylyltransferase	*Synechococcus* sp. WH 7805	cyanobacteria

133	37523829	glucose-1-phosphate adenylyltransferase	*Gloeobacter violaceus* PCC 7421	cyanobacteria
134	33861326	glucose-1-phosphate adenylyltransferase	*Prochlorococcus marinus* str. CCMP1986	cyanobacteria
135	33240292	glucose-1-phosphate adenylyltransferase	*Prochlorococcus marinus* str. CCMP1375	cyanobacteria
136	22298830	glucose-1-phosphate adenylyltransferase	*Thermosynechococcus elongatus* BP-1	cyanobacteria
137	218248785	glucose-1-phosphate adenylyltransferase	*Cyanothece* sp. PCC 8801	cyanobacteria
138	186686123	glucose-1-phosphate adenylyltransferase	*Nostoc punctiforme* PCC 73102	cyanobacteria
139	124023387	glucose-1-phosphate adenylyltransferase	*Prochlorococcus marinus* str. MIT 9303	cyanobacteria
140	86608545	glucose-1-phosphate adenylyltransferase	*Synechococcus* sp. JA-2-3B'a(2-13)	cyanobacteria
141	86606226	glucose-1-phosphate adenylyltransferase	*Synechococcus* sp. JA-3-3Ab	cyanobacteria
142	33862839	glucose-1-phosphate adenylyltransferase	*Prochlorococcus marinus* str. MIT 9313	cyanobacteria
143	33865652	glucose-1-phosphate adenylyltransferase	*Synechococcus* sp. WH 8102	cyanobacteria
144	17232137	glucose-1-phosphate adenylyltransferase	*Nostoc* sp. PCC 7120	cyanobacteria
145	159903534	glucose-1-phosphate adenylyltransferase	*Prochlorococcus marinus* str. MIT 9211	cyanobacteria
146	126696167	glucose-1-phosphate adenylyltransferase	*Prochlorococcus marinus* str. MIT 9301	cyanobacteria
147	124025514	glucose-1-phosphate adenylyltransferase	*Prochlorococcus marinus* str. NATL1A	cyanobacteria
148	123968364	glucose-1-phosphate adenylyltransferase	*Prochlorococcus marinus* str. AS9601	cyanobacteria
149	123966049	glucose-1-phosphate adenylyltransferase	*Prochlorococcus marinus* str. MIT 9515	cyanobacteria
150	113954397	glucose-1-phosphate adenylyltransferase	*Synechococcus* sp. CC9311	cyanobacteria
151	218437477	glucose-1-phosphate adenylyltransferase	*Cyanothece* sp. PCC 7424	cyanobacteria
152	78779161	glucose-1-phosphate adenylyltransferase	*Prochlorococcus marinus* str. MIT 9312	cyanobacteria
153	16332282	glucose-1-phosphate adenylyltransferase	*Synechocystis* sp. PCC 6803	cyanobacteria
154	257061844	glucose-1-phosphate adenylyltransferase	*Cyanothece* sp. PCC 8802	cyanobacteria
155	56750930	glucose-1-phosphate adenylyltransferase	*Synechococcus elongatus* PCC 6301	cyanobacteria
156	220910118	glucose-1-phosphate adenylyltransferase	*Cyanothece* sp. PCC 7425	cyanobacteria
157	81299414	glucose-1-phosphate adenylyltransferase	*Synechococcus elongatus* PCC 7942	cyanobacteria
158	78212786	glucose-1-phosphate adenylyltransferase	*Synechococcus* sp. CC9605	cyanobacteria
159	78184800	glucose-1-phosphate adenylyltransferase	*Synechococcus* sp. CC9902	cyanobacteria

160	158335435	glucose-1-phosphate adenylyltransferase	*Acaryochloris marina* MBIC11017	cyanobacteria
161	158335089	glucose-1-phosphate adenylyltransferase	*Acaryochloris marina* MBIC11017	cyanobacteria
162	113477795	glucose-1-phosphate adenylyltransferase	*Trichodesmium erythraeum* IMS101	cyanobacteria
163	75908241	glucose-1-phosphate adenylyltransferase	*Anabaena variabilis* ATCC 29413	cyanobacteria
164	172037571	glucose-1-phosphate adenylyltransferase	*Cyanothece* sp. ATCC 51142	cyanobacteria
165	172035903	glucose-1-phosphate adenylyltransferase	*Cyanothece* sp. ATCC 51142	cyanobacteria
166	170076729	glucose-1-phosphate adenylyltransferase	*Synechococcus* sp. PCC 7002	cyanobacteria
167	157413198	glucose-1-phosphate adenylyltransferase	*Prochlorococcus marinus* str. MIT 9215	cyanobacteria
168	72382015	glucose-1-phosphate adenylyltransferase	*Prochlorococcus marinus* str. NATL2A	cyanobacteria
169	148242352	glucose-1-phosphate adenylyltransferase	*Synechococcus* sp. RCC307	cyanobacteria
170	148239634	glucose-1-phosphate adenylyltransferase	*Synechococcus* sp. WH 7803	cyanobacteria
171	166365546	glucose-1-phosphate adenylyltransferase	*Microcystis aeruginosa* NIES-843	cyanobacteria
172	254526910	glucose-1-phosphate adenylyltransferase	*Prochlorococcus marinus* str. MIT 9202	cyanobacteria
173	254432095	glucose-1-phosphate adenylyltransferase	*Cyanobium* sp. PCC 7001	cyanobacteria
174	307151922	glucose-1-phosphate adenylyltransferase	*Cyanothece* sp. PCC 7822	cyanobacteria
175	298492804	glucose-1-phosphate adenylyltransferase	*Nostoc azollae* 0708	cyanobacteria
176	332709240	glucose-1-phosphate adenylyltransferase	*Lyngbya majuscula* 3L	cyanobacteria
177	354565610	glucose-1-phosphate adenylyltransferase	*Fischerella* sp. JSC-11	cyanobacteria
178	354556049	glucose-1-phosphate adenylyltransferase	*Cyanothece* sp. ATCC 51472	cyanobacteria
179	354553549	glucose-1-phosphate adenylyltransferase	*Cyanothece* sp. ATCC 51472	cyanobacteria
180	352094384	glucose-1-phosphate adenylyltransferase	*Synechococcus* sp. WH 8016	cyanobacteria
181	284929352	glucose-1-phosphate adenylyltransferase	cyanobacterium UCYN-A	cyanobacteria
182	334120832	glucose-1-phosphate adenylyltransferase	*Microcoleus vaginatus* FGP-2	cyanobacteria
183	300865383	glucose-1-phosphate adenylyltransferase	*Oscillatoria* sp. PCC 6506	cyanobacteria
184	282899378	glucose-1-phosphate adenylyltransferase	*Cylindrospermopsis raciborskii* CS-505	cyanobacteria
185	282895605	glucose-1-phosphate adenylyltransferase	*Raphidiopsis brookii* D9	cyanobacteria
186	209527099	glucose-1-phosphate adenylyltransferase	*Arthrospira maxima* CS-328	cyanobacteria

187	67924676	glucose-1-phosphate adenylyltransferase	*Crocosphaera watsonii* WH 8501	cyanobacteria
188	260436638	glucose-1-phosphate adenylyltransferase	*Synechococcus* sp. WH 8109	cyanobacteria
189	303273364	adp-glucose pyrophosphorylase	*Micromonas pusilla* CCMP1545	green alga
190	303271247	adp-glucose pyrophosphorylase	*Micromonas pusilla* CCMP1545	green alga
191	255070935	adp-glucose pyrophosphorylase	*Micromonas* sp. RCC299	green alga
192	255080070	adp-glucose pyrophosphorylase	*Micromonas* sp. RCC299	green alga
193	159470605	ADP-glucose pyrophosphorylase large subunit	*Chlamydomonas reinhardtii*	green alga
194	159467349	ADP-glucose pyrophosphorylase small subunit	*Chlamydomonas reinhardtii*	green alga
195	308814250	AGPLU2 (ISS)	*Ostreococcus tauri*	green alga
196	308806175	AGPSU1 (ISS)	*Ostreococcus tauri*	green alga
197	302849075	hypothetical protein VOLCADRAFT_76956	*Volvox carteri* f. nagariensis	green alga
198	302840808	hypothetical protein VOLCADRAFT_75183	*Volvox carteri* f. nagariensis	green alga
199	145356323	predicted protein	*Ostreococcus lucimarinus* CCE9901	green alga
200	145349062	predicted protein	*Ostreococcus lucimarinus* CCE9901	green alga
201	302825850	hypothetical protein SELMODRAFT_138695	*Selaginella moellendorffii*	moss
202	302815217	hypothetical protein SELMODRAFT_129625	*Selaginella moellendorffii*	moss
203	302802313	hypothetical protein SELMODRAFT_117069	*Selaginella moellendorffii*	moss
204	302800351	hypothetical protein SELMODRAFT_115472	*Selaginella moellendorffii*	moss
205	302798196	hypothetical protein SELMODRAFT_233627	*Selaginella moellendorffii*	moss
206	302788037	hypothetical protein SELMODRAFT_267891	*Selaginella moellendorffii*	moss
207	302783933	hypothetical protein SELMODRAFT_149205	*Selaginella moellendorffii*	moss
208	302773934	hypothetical protein SELMODRAFT_231637	*Selaginella moellendorffii*	moss
209	302769466	hypothetical protein SELMODRAFT_169778	*Selaginella moellendorffii*	moss
